# Engineering of the Recombinant Expression and PEGylation Efficiency of the Therapeutic Enzyme Human Thymidine Phosphorylase

**DOI:** 10.3389/fbioe.2021.793985

**Published:** 2021-12-17

**Authors:** Christos S. Karamitros, Catrina M. Somody, Giulia Agnello, Scott Rowlinson

**Affiliations:** Aeglea Biotherapeutics, Austin, TX, United States

**Keywords:** enzyme engineering, catalytic activity, recombinant expression, PEGylation, thymidine phosphorylase

## Abstract

Human thymidine phosphorylase (HsTP) is an enzyme with important implications in the field of rare metabolic diseases. Defective mutations of HsTP lead to mitochondrial neurogastrointestinal encephalomyopathy (MNGIE), a disease with a high unmet medical need that is associated with severe neurological and gastrointestinal complications. Current efforts focus on the development of an enzyme replacement therapy (ERT) using the *Escherichia coli* ortholog (EcTP). However, bacterial enzymes are counter-indicated for human therapeutic applications because they are recognized as foreign by the human immune system, thereby eliciting adverse immune responses and raising significant safety and efficacy risks. Thus, it is critical to utilize the HsTP enzyme as starting scaffold for pre-clinical drug development, thus de-risking the safety concerns associated with the use of bacterial enzymes. However, HsTP expresses very poorly in *E. coli*, whereas its PEGylation, a crucial chemical modification for achieving long serum persistence of therapeutic enzymes, is highly inefficient and negatively affects its catalytic activity. Here we focused on the engineering of the recombinant expression profile of HsTP in *E. coli* cells, as well as on the optimization of its PEGylation efficiency aiming at the development of an alternative therapeutic approach for MNGIE. We show that phylogenetic and structural analysis of proteins can provide important insights for the rational design of N’-terminus-truncation constructs which exhibit significantly improved recombinant expression levels. In addition, we developed and implemented a criteria-driven rational surface engineering strategy for the substitution of arginine-to-lysine and lysine-to-arginine residues to achieve more efficient, homogeneous and reproducible PEGylation without negatively affecting the enzymatic catalytic activity upon PEGylation. Collectively, our proposed strategies provide an effective way to optimize enzyme PEGylation and *E. coli* recombinant expression and are likely applicable for other proteins and enzymes.

## Introduction

Thymidine phosphorylases (E.C. 2.4.2.4.) are enzymes that play a pivotal role in the salvage pathway of pyrimidine nucleoside metabolism in both lower and higher organisms (Desgranges et al., 1981; Okuyama et al., 1996; Pugmire and Ealick, 2002). They catalyze the reversible phosphorolysis of deoxythymidine (dThd) and deoxyuridine (dUrd) to 2-deoxyribose-1-phosphate and to their respective bases, with the latter being fluxed towards nucleotide synthesis. Humans have three cytosolic pyrimidine nucleoside phosphorylases, namely a thymidine phosphorylase (HsTP; encoded by *TYMP* gene) and two uridine phosphorylases (HsUP1 & HsUP2 encoded by *UPP1* and *UPP2* genes respectively) that have totally distinct structural and biochemical features (Johansson, 2003; Norman et al., 2004). HsTP does not show any significant amino acid sequence similarity against any of the HsUP enzymes whereas HsUP1 and HsUP2 share 66% sequence identity. HsTP is a highly specific N’-ribosyl phosphorylase displaying *k*_cat_/K_M_ values against dThd and dUrd in the range of 10^5^ M^−1^ s^−1^ and 10^4^ M^−1^ s^−1^ respectively (Deves et al., 2014; Schwartz et al., 2010), whereas HsUP enzymes exhibit higher specificity against uridine (Urd) (*k*_cat_/K_M_ ∼10^5^ M^−1^ s^−1^) and to a lesser extent towards dUrd and dThd (*k*_cat_/K_M_ ∼10^3^ M^−1^ s^−1^) (Liu et al., 1998; Renck et al., 2010). Thymidine phosphorylases from lower organisms like *Bacillus stearothermophilus* (BsTP) and *Escherichia coli* (EcTP) exhibit similar fold and belong to the same family (family II) of nucleoside phosphorylases with HsTP, while their amino acid sequence identity is ∼40%. Particularly, EcTP is highly active against dThd showing a *k*_cat_/K_M_ ∼10^6^ M^−1^ s^−1^ (Panova et al., 2008; Gbaj et al., 2006).

HsTP has attracted the interest of intense research during the last decades primarily due to its implication in tumor cell growth (Matsushita et al., 1999; Moghaddam et al., 1995). Importantly, it has been shown that the platelet-derived endothelial cell growth factor (PD-ECGF) is identical to HsTP and is involved in angiogenic and endothelial cell chemotactic activities (Sumizawa et al., 1993). Overexpression and elevated HsTP activity have been detected and measured in plasma of cancer patients and have been associated with invasive and metastatic profiles of various cancer types including bladder, colorectal, ovarian and pancreatic tumors (Pauly et al., 1977; Takebayashi et al., 1996a; Takebayashi et al., 1996b; Reynolds et al., 1994). HsTP has been shown to degrade the anti-viral drug trifluorothymidine (TFT) as well as other fluoropyrimidines and chemotherapeutic agents (Patterson et al., 1995). These observations prompted the in-depth study of HsTP’s kinetic properties and the elucidation of its catalytic mechanism (Schwartz et al., 2010; Birck and Schramm, 2004; Oh and el Kouni, 2018) to aid the development of potent inhibitors (de Moura Sperotto et al., 2019; Pérez-Pérez et al., 2005). Indeed, the inhibitor 5-chloro-6-[1-(2-iminopyrrolidinyl) methyl] uracil hydrochloride (TPI) is one of the most potent HsTP inhibitors (Ki ∼ 20 nM), capable of inducing considerable reduction in tumor growth in mice (Matsushita et al., 1999).

Beyond its role and implications in tumor biology, HsTP has also been studied within the context of rare metabolic diseases. Mutations in the HsTP gene (*TYMP*) which result in either partial or complete loss of enzymatic activity, cause mitochondrial neurogastrointestinal encephalomyopathy (MNGIE) which is an autosomal recessive disease (Slama et al., 2005). HsTP deficiency leads to elevated systemic levels of dThd and dUrd and patients with MNGIE suffer from neurological and gastrointestinal symptoms (Hirano, 1993; Teitelbaum et al., 2002). It has been well-documented that the underlying mechanism for all these symptoms and manifestations is associated with a disorder known as mitochondrial DNA (mtDNA) depletion syndrome (Pacitti et al., 2018; El-Hattab and Scaglia, 2013). That is, build-up of dThd and dUrd leads to an imbalance of nucleotide levels which, in turn, negatively affects the mitochondrial DNA replication that relies on the cytoplasmic pool of nucleotides for proper function. Current management of MNGIE has been primarily supportive with the main goal being to ease the disease manifestations. Thus, MNGIE therapy represents an unmet medical need that requires further attention for the development of effective treatments.

Recent efforts for the development of MNGIE therapies have centered on the allogeneic hematopoietic stem cell transplantation (HSCT) (Halter et al., 2015), on AAV- and gene-editing-based gene therapy approaches (Torres-Torronteras et al., 2018; (Torres-Torronteras et al., 2014; Torres-Torronteras et al., 2016; Parés et al., 2021), as well as on an enzyme replacement therapy (ERT) utilizing EcTP which is encapsulated inside erythrocytes (EE-EcTP) for long plasma persistence and immunogenicity minimization (Bax et al., 2019). However, the HSCT has exhibited a high mortality rate of ∼63% (Yadak et al., 2018; Filosto et al., 2012) and the gene therapy approaches, while highly promising, have been explored only in preclinical settings thus far. In addition, the erythrocyte encapsulation strategy that utilizes the bacterial EcTP is associated with a high treatment burden because it requires hospital visits for the administration of the drug. Importantly, bacterial enzymes as human therapeutics are often encumbered with significant safety and efficacy risks because they are recognized as foreign species by the human immune system and, in turn, elicit adverse immune responses and clearing anti-drug antibodies (Pokrovsky et al., 2016; Lipsky et al., 2014). Thus, the use of HsTP for the development of an ERT could represent a more advantageous and potentially safer approach relative to the aforementioned strategies. Unlike in rare diseases where patients completely lack the endogenous protein (e.g., large deletions and intron inversions in haemophilia A) (Levinson et al., 1992) and administration of its functional form can elicit immune responses, in case of MNGIE the vast majority of the mutations result in poor catalytic activity and not in pre-mature termination of the translation. Therefore, most patients would be considered to have natural, cross-reactive immunogenic HsTP. As a result, they are tolerized against HsTP and unlikely to develop significant antibody response to ERT.

In an attempt to form the basis for an alternative therapeutic approach for MNGIE, in the present study we report on the engineering of recombinant expression properties of HsTP in *E. coli*, as well as its PEGylation optimization. Expression of the mature wild-type HsTP in *E. coli* yielded low levels of total active protein. Analysis of the HsTP crystal structure and direct comparison to its *E. coli* ortholog combined with phylogenetic analysis allowed the design of N’-terminal truncations that did not only improve the recombinant expression levels by several fold, but also improved the enzyme’s catalytic activity against its secondary substrate dUrd. In addition, random lysine PEGylation of wild-type HsTP resulted in poor PEGylation efficiency, as evidenced by the presence of multiple, distinct PEG-mer molecule species while it negatively affected its catalytic activity against its natural substrates. By developing and implementing a rational surface engineering strategy, we identified suitable arginine residues which were substituted with lysine for more efficient, homogeneous and reproducible PEGylation. Conversely, lysine residues whose conjugation with PEG could negatively impact catalytic activity were substituted with arginine to prevent the cross-linking chemical modification, thereby maintaining equal levels of catalytic activity prior and post PEGylation. While ameliorating the biological symptoms is the ultimate goal of therapeutics, an essential component, particularly for protein therapeutics, is the ability to efficiently and reproducibly manufacture them. Our present study proposes two engineering strategies, for the PEGylation and recombinant expression optimization of HsTP, that could be applied towards the development of a parenteral enzyme therapy for MNGIE.

## Materials and Methods

### Cloning of HsTP and EcTP Genes

Amino acid sequences for wild-type HsTP and EcTP were codon-optimized for *E. coli* using the online tool provided by Integrated DNA Technologies (https://www.idtdna.com/pages/tools/codon-optimization-tool), and gene blocks with the resultant sequences were used as a template for polymerase chain reaction (PCR) amplification. Primers were designed to amplify the HsTP and EcTP gene fragments as follows: the 5′ primer introduced a His^6^-tag at the N-terminus and an NcoI restriction site; the 3′ primer introduced an EcoRI restriction site. PCR amplification was carried out following the Kapa HiFi protocol (Roche) with an annealing temperature of 60°C and a 2-min extension at 72°C for a total of 25 cycles. The amplicons were digested with NcoI and EcoRI, gel-purified, ligated into pET28a (Novagen) with T4 DNA ligase, and transformed into MC1061 cells (Lucigen). Primers used to generate the variants in this study are provided in Supplementary Table S2. Positive clones were screened by colony PCR using as the forward primer, one specific primer for the insert and as the reverse primer, one specific primer for the plasmid backbone.

### Expression and Purification of HsTP and EcTP Constructs

#### Expression

HsTP and EcTP constructs either in BL21 (DE3) or C41 (DE3) cells were cultured in terrific broth (TB) media supplemented with 50 μg/ml of kanamycin at 37°C until OD at 600 nm reached between and 0.8 and 1 units when standard shake flasks were used, or OD of 5 when ultra-yield flasks (Thompson) were used. Then, for HsTP^199^ and EcTP, 0.5 mM IPTG was added, temperature was reduced to 30°C, and expression was allowed to proceed for 20–22 h. Expression-optimized variants, including HsTP^218^, were induced with 0.1 mM IPTG and expressed at 16°C for 40 h.

#### Purification by Immobilized Metal Affinity Chromatography

Cells were pelleted by centrifugation at 4°C and resuspended in lysis buffer consisting of 50 mM Na_2_HPO_4_, 300 mM NaCl, 10 mM imidazole, 1 mM PMSF protease inhibitors, and 1 μl of 1 mg/ml DNase I for each ml of cell suspension, at pH 8. Resuspended samples were kept on ice and lysed by sonication. After sonication, the samples were centrifuged at 12,000 ×g for 1 h at 4°C, the supernatant was decanted, filtered through a 0.2 μm filter, and the pellet discarded. Lysate was mixed with Ni-NTA resin (Qiagen) (Ni-NTA resin volume was in the range of 4–8 ml of slurry mixture depending on the expected protein amount in the cell lysate) which had been previously equilibrated with 20 bed volumes of lysis buffer (50 mM Na_2_HPO_4_, 300 mM NaCl, 10 mM Imidazole, pH 8). The lysate-resin mixture was incubated at 4°C for 2 h (batch purification) and subsequently was applied by gravity flow to a polypropylene column. The column was washed with 20 bed volumes of washing buffer (50 mM Na_2_HPO_4_, 300 mM NaCl, 20 mM Imidazole, pH 8) followed by elution with three bed volumes of elution buffer (50 mM Na_2_HPO_4_, 300 mM NaCl, 300 mM Imidazole, pH 8). The eluted protein was buffer-exchanged against 20 mM Tris-Cl and 20 mM NaCl, pH 7.5 using 10 kDa MWCO protein concentrator tubes (Amicon) and was subjected to ion-exchange chromatography described below.

#### Q-Sepharose Purification

Following buffer exchange, the IMAC-eluted protein was applied by gravity flow to a polypropylene column loaded with 2 ml of Q-FF resin which had been previously equilibrated with 20 bed volumes of binding and washing buffer (20 mM Tris-Cl, 20 mM NaCl, pH 7.5). The column was washed with 20 bed volumes of binding and washing buffer, then eluted with a 50–500 mM NaCl gradient. Purity of the protein elutions was assessed by SDS-PAGE, and the fractions with the highest concentration and purity were pooled. HsTP was observed to elute between 100 and 200 mM NaCl. Purified HsTP was aliquoted, mixed with 15% (v/v) final glycerol concentration, flash-frozen with liquid nitrogen, and stored at −80°C for future use.

#### Immunoblotting

Immunoblotting for the detection of expressed HsTP was carried out according to standard protocols (Slieman and Leheste, 2020). Briefly, protein concentration was calculated using NanoDrop (One microvolume UV-Vis spectrophotometer from Thermo Scientific) by recording the A^280^. For crude extracts, A^280^ of 1 was assumed to be equivalent to 1 mg/ml whereas for HsTP and EcTP, the respective theoretical molar extinction coefficient values (ε^HsTP^ = 23,490 M^−1^ cm^−1^and ε^EcTP^ = 24,410 M^−1^ cm^−1^ as calculated from Expasy’s protparam online tool based on the primary amino acid sequence of the proteins) were used for the conversion of absorbance to molar concentration. Typical protein amount loaded per well was in the range of 10–15 μg. The protein samples were analyzed by SDS-PAGE and subsequently, the separated proteins were transferred onto a nitrocellulose membrane in transfer buffer (25 mM Tris-Cl, 190 mM glycine, 0.1% SDS) at a fixed current of 10 mA overnight at 4°C. Membranes were blocked with 5% skim milk dissolved in TBST buffer for 2 h at room temperature, followed by incubation with monoclonal anti-His^6^ antibodies (Sigma-Aldrich, SAB2702218). Incubation with secondary goat anti-mouse HRP-linked antibodies (ThermoFisher Scientific, G-21040) was carried out for 1 h at room temperature. Upon immunoblotting, bands were detected using an enhanced chemiluminescence (ECL) kit (SuperSignal West Pico PLUS from ThermoFisher Scientific, 34580).

#### Steady-State Kinetic Analysis of HsTP and EcTP

Steady-state kinetic characterization of HsTP and EcTP against dThd and dUrd was performed by continuously monitoring the decrease in absorbance of dThd and dUrd upon depyrimidination at 290 and 282 nm, respectively. Enzyme concentration in the range of 10–20 nM was used for all the steady-state kinetic measurements. Reactions took place in assay buffer consisting of 20 mM Hepes and 50 mM KH_2_PO_4_, pH 7.5 in a final volume of 1 ml placed in UV cuvettes with a pathlength of 1 cm. Prior to the addition of the enzyme, cuvettes were equilibrated at 37°C for 20 min using a heating block (VWR). The reaction progress was typically monitored for 2 min using a Jasco V750 spectrophotometer with a temperature-controlled cuvette holder and the absorbance was converted to concentration using the extinction coefficient of dThd and dUrd (Δε^290^ = 1,000 M^−1^ cm^−1^ and Δε^282^ = 1,370 M^−1^ cm^−1^) (Krenitsky et al., 1976). The obtained v/[E] (initial velocity/total enzyme concentration) values from the linear region of the reaction progress curves with <10% of substrate conversion were plotted against the respective substrate concentrations, and the steady-state kinetic parameters *k*_cat_ and *k*_cat_/K_M_ were calculated by nonlinear regression using the Michaelis−Menten model (Eq. 1 below) analyzed by the SoftZymics software (Igor Pro, Wavemetrics).

$$v=\left(kcat\;\times \left[S\right]\right)/\left(K_{M}+S\right)$$ (1)

#### Analytical Size-Exclusion Chromatography of HsTP

The purified protein samples were diluted in PBS buffer to a final concentration of approximately 2 mg/ml. A total of 2 µl of diluted samples was injected on a size exclusion column (TSKgel G4000SWxl, 7.8 × 300 mm, 8 µ particle size, from Tosoh). Isocratic elution was performed at a flow rate of 0.3 ml/min, using a Thermo ultimate HPLC system. The mobile phase contained 90% of phosphate buffer (50 mM sodium phosphate, 200 mM sodium chloride, adjusted to pH 7.2) and 10% ethanol. The column eluent was monitored by UV detection at 214 nm. The BioRad gel filtration standard was used as molecular weight markers.

#### Phylogenetic Analysis of Thymidine Phosphorylase Enzymes

Phylogenetic analysis was performed by using the program Geneious prime. The full-length amino acid sequences of HsTP and EcTP deposited in the Uniprot database (P19971 and P07650) were used as query sequences to perform massive BLAST alignments and thus, determine conserved residues. Geneious is directly connected to the NCBI database and searches for homologous sequences to the query entry. For restricting the search exclusively for either eukaryotic or prokaryotic thymidine phosphorylase enzymes, the commands “Eukaryotes [organism]” or “Prokaryotes [organism]” were used in the “entrez query” option respectively. The maximum number of thymidine phosphorylase hits to be searched for from the NCBI database was set to 1,000.

#### Translation Initiation Rate *In-Silico* Analysis

Translation initiation rates (TIR) were calculated using the online webserver De Novo DNA at the following URL: https://salislab.net/software/. The mRNA of each construct starting from the transcriptional start site and ending at the transcriptional terminator sequence was subjected to analysis based on the ribosome binding site (RBS) predict-mode algorithm using *E. coli* as the host organism for recombinant expression. The RBS calculator in the predict-mode identifies all the starting codons in the mRNA sequence and calculates a translation initiation rate (TIR) in arbitrary units (a.u.; the larger the value, the higher the TIR) for each of them according to its statistical thermodynamics algorithm as described elsewhere (Salis et al., 2009). The TIR calculations are accompanied by ribosome binding Gibbs free energy calculations that represent the quantification of the interaction between the ribosome and the mRNA that, in turn, affects the TIR. The reported ΔG_total_ is defined as follows: ΔG_total_ = ΔG_mRNA-rRNA_ + ΔG_spacing_ + ΔG_start_ − ΔG_standby_ − ΔG_mRNA,_ where ΔG_mRNA-rRNA_ is the energy released when the last nine nucleotides of the *E. coli* 16S rRNA hybridizes and co-folds to the mRNA sub-sequence. ΔG_start_ is the energy released when the start codon hybridizes to the initiating tRNA anticodon loop. ΔG_spacing_ is the free energy cost caused by a non-optimal physical distance between the 16S rRNA binding site and the start codon. ΔG_mRNA_ is the work required to unfold the mRNA sub-sequence when it folds to its most stable secondary structure and ΔG_standby_ is the work required to unfold any secondary structures sequestering the standby site after the 30S complex assembly.

#### Site-Directed Mutagenesis and Generation of PEGylation Variants

For the generation of the PEGylation variants HsTP^240^ and HsTP^241^ site-directed mutagenesis was performed following the overlap extension PCR methodology (Nelson and Fitch, 2011). Briefly, this method comprises three successive amplification steps and involves four primers as follows: two external ones that cover the 5′- and 3′- ends of the parental sequence and two additional ones which carry the desired mutations to be incorporated in the final sequence. Two independent PCR reactions (PCR1: forward external primer covering the 5′ combined with the reverse carrying the mutations and PCR2: reverse external at the 3′ combined with forward carrying the mutations) were performed to amplify two fragments which overlap at the regions flanking the mismatches. The two amplified fragments were agarose gel-purified and in a final third step, they were combined in equal molar quantities with the initial external primers and were subjected to the last PCR reaction resulting in the final amplicon that carries the desired point mutations. The PCR amplicons along with the pET28a plasmid were digested overnight at 37°C with NcoI/EcoRI and ultimately were cloned using T4 DNA ligase. The incorporated mutations were sequence-verified by Sanger sequencing.

#### Preparation of PEGylated HsTP and EcTP

Conjugation reactions with methoxy-5-kDa-PEG-succinimidyl-succinate (mPEG^5 kDa^, NOF Corporation) were performed in 100 mM Na_2_HPO_4_, pH 8.5 using purified HsTP^199^ and EcTP as described above in the respective section. Final enzyme concentration in the reaction was ∼115 μM and depending on the protein: PEG ratio, the respective concentration of PEG was adjusted accordingly: 1,150, 2,300, and 3,450 μM for 1:10, 1:20, and 1:30 M ratio, respectively. The lyophilized PEG was weighed and was added directly into a 2 ml tube containing either purified HsTP^199^ or EcTP at a volume of 1 ml. Immediately after the addition of the PEG, the tube was vortexed continuously for 30 s, followed by incubation at room temperature for 30 min under rotating conditions. Subsequently, the mixture was exhaustively buffer-exchanged to remove excess of unreactive PEG using protein concentrators with a 50-kDa MWCO (Thermo Scientific). HsTP^240^ and HsTP^241^ PEGylation variants and HsTP^218^ were conjugated following the same process except for the buffer, which was 50 mM Na_2_HPO_4_ and 50 mM NaCl, pH 8.0, and the final enzyme concentration was 200 μM. Following the final buffer exchange step, PEGylation homogeneity was assessed by SDS-PAGE and steady-state kinetics was performed using dThd as the substrate. PEGylated enzymes were buffer-exchanged against 50 mM Na_2_HPO_4_ and 50 mM NaCl, pH 7.0, mixed with a 15% final concentration of glycerol, flash-frozen in liquid nitrogen, and stored at −80°C.

## Results

### HsTP Is Poorly Expressed in *E. Coli*

**FIGURE 1:**
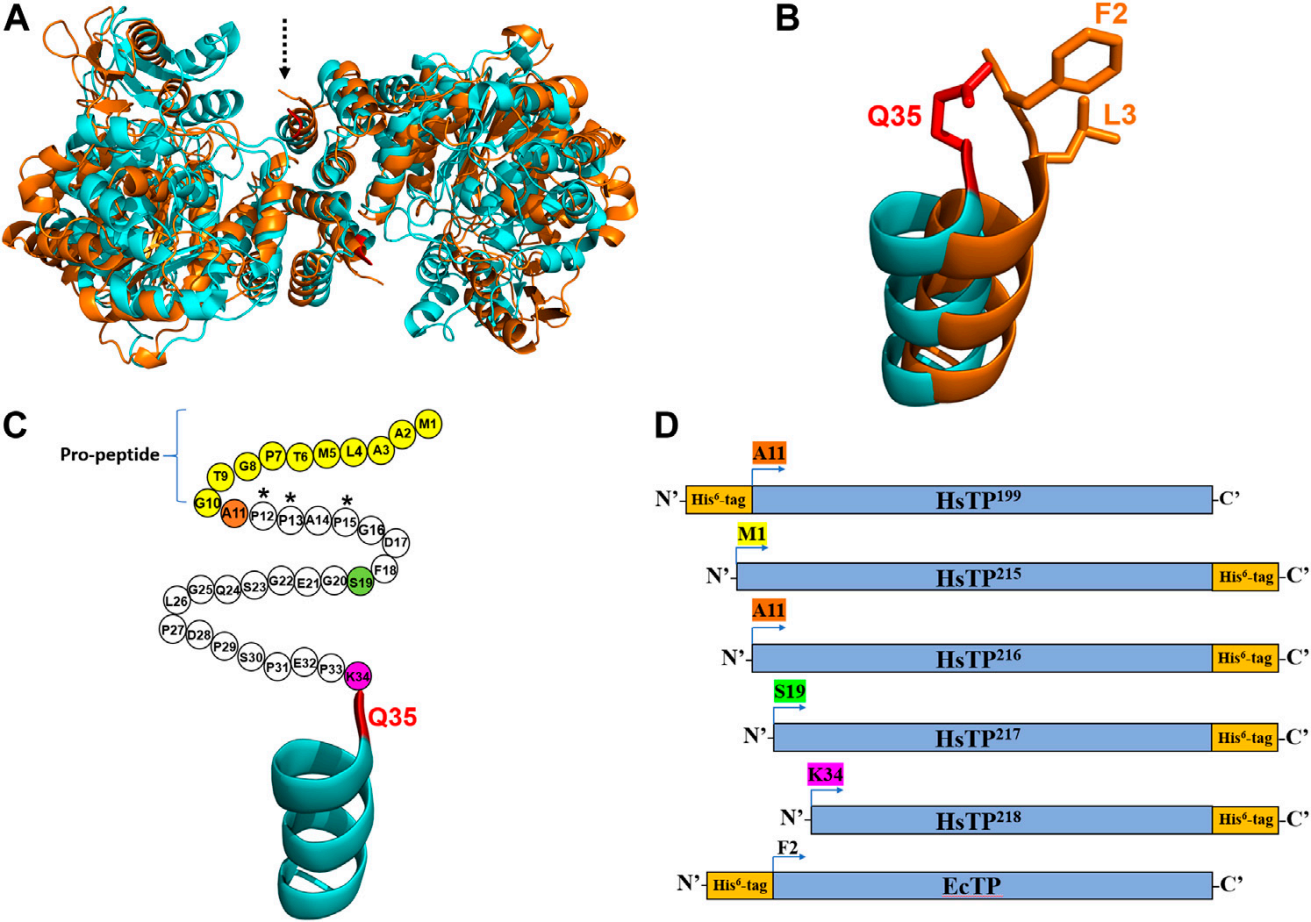
The crystal structures and the constructs of HsTP and EcTP. **(A)** Overlaid crystal structures of HsTP (PDB: 2WK6) and EcTP (PDB: 4LHM) shown in cyan and orange color respectively. Dashed arrow indicates the N’-terminus of each enzyme. **(B)** Zoomed, overlaid α-helices located at the N’-terminus of HsTP (cyan) and EcTP (orange). Q35 residue shown in red sticks is the first amino acid residue that is structurally-resolved in case of HsTP, with F2 and L3 (shown in orange sticks) occupying the respective structurally-equivalent positions of EcTP. **(C)** N’-terminal domain of HsTP. Unstructured region without electron density is shown as circles, where each circle represents the respective amino acid residue. Pro-peptide corresponds to the first ten residues and are shown in yellow. Residues A11, S19 and K34 are shown in orange, green and dark pink and represent the positions at which the HsTP constructs were truncated (constructs were truncated at the residue preceding A11, S19 and K34). **(D)** Cartoon representation of all the HsTP and EcTP constructs tested in the present study. The position of His^6^-tag as well as the truncation positions are also shown. Starting methionine and a glycine residue (result of the cloning process as described in the Methods section) are preceding the starting position (A11, S19, K34, F2) of all constructs shown in this panel. Structural representations were prepared using PyMol 2.4.1 (Schrödinger, LLC).

Successful recombinant expression of HsTP in *E. coli* has been previously described by other groups with soluble expression levels reported in the range of ∼3 mg/L of culture (Schwartz et al., 2010). Available information deposited in public research databases (e.g., NCBI and Uniprot) suggests that HsTP harbors a N’-terminal 10-residue long pro-peptide (Uniprot entry P19971) which is cleaved during a post-translational modification and processing step, thereby yielding the final mature polypeptide chain. While the biological role of this pro-peptide, as well as its impact on the enzyme’s biochemical features remain largely unclear, all prior studies focusing on the biochemical characterization of HsTP used the putatively mature version of the protein with the first ten residues at the N’-terminal site truncated (Schwartz et al., 2010; Deves et al., 2014). Accordingly, we designed a HsTP gene carrying a His^6^-tag at the N’-terminus, codon-optimized for *E. coli* expression, and devoid of the first ten residues corresponding to the pro-peptide sequence, cloned into pET28-a plasmid (construct termed HsTP^199^, Figure 1D) and tested its recombinant expression in the *E. coli* BL21 (DE3) strain. Indeed, the levels of the recovered active enzyme were in the range of 3–5 mg/L of TB culture medium (yield is defined as the amount of purified protein obtained after two purification steps as described in the Methods section, Table 1), while a considerable amount of the produced protein was detected in an insoluble inclusion body fraction (∼50% more than the soluble) (Supplementary Figures S1, S2). Multiple attempts to increase the expression titers (both soluble and total amount) by 1) switching to C41 (DE3) *E. coli* expression strain which has been shown to benefit soluble expression levels (Kwon et al., 2015) (Supplementary Figures S1, S2), 2) altering expression conditions (e.g., Isopropyl-β-d-1-thiogalactopyranoside; IPTG concentrations, temperature, OD^600^ at the induction, culture media) (Rosano and Ceccarelli, 2014; Gutiérrez-González et al., 2019), and 3) testing a high-copy-number plasmid (pJC-20) (Clos and Brandau, 1994) (Supplementary Figure S3) failed to improve expression levels of HsTP^199^. Taken together, these data showed that achieving high levels of HsTP bacterial expression would necessitate a more in-depth analysis and additional extensive experimental efforts.

**TABLE 1:** Summary of the HsTP and EcTP constructs and their expression yields^a^.

Construct	His^6^-tag	Range of expression yield/L	Plasmid	Medium	Temperature (°C)	IPTG (mM)
**HsTP** ^**199**^	N’	3–5 mg	pET28a	TB	30	0.5
**HsTP** ^**215**^	C’	0.5–1 mg	pET28a	TB	30	0.5
**HsTP** ^**216**^	C’	1–2 mg	pET28a	TB	30	0.5
**HsTP** ^**217**^	C’	4–5 mg	pET28a	TB	30	0.5
**HsTP** ^**218**^	C’	2.5–3 mg	pET28a	TB	30	0.5
**HsTP** ^**218**^	C’	50–70 mg	pET28a	TB	16	0.1
**EcTP**	N’	100–120 mg	pET28a	TB	30	0.5

a Expression yield refers to the soluble protein that was recovered after employing the standard 2-step purification scheme described in the Methods section.

### HsTP and EcTP Have Distinct N’-Terminal Domains and Recombinant Expression Profiles

Considering the fact that we used a synthetic, codon-optimized gene for optimal expression in *E. coli*, it was curious as to why all our efforts failed to yield large quantities of HsTP. High level expression of recombinant human proteins in *E. coli* can be very challenging to optimize as multiple molecular factors such as mRNA stability and structure (Simonetti et al., 2009; de Smit and van Duin, 1994), translation initiation rates (Espah Borujeni et al., 2014) and structural features of the protein (particularly at the N’-terminus) (Wruck et al., 2017) can play a pivotal role. The latter evidence motivated us to inspect the crystal structure of HsTP (PDB entry 2WK6) (Mitsiki et al., 2009) and compare it to the respective structure of the bacterial ortholog EcTP (PDB entry 4LHM) (Timofeev et al., 2014). The two enzymes belong to the family II of nucleoside phosphorylases, they are relatively close structural homologs (C_α_-RMSD ∼5.2 Å) (Figure 1A) and they share 38.4% amino acid sequence identity. Interestingly, we found that none of the available HsTP crystal structures deposited in the protein data bank (PDB) (2WK6, 1UOU, 2J0F) show a well-resolved secondary structure of the N’-terminal 10-residue pro-peptide. In fact, a longer sequence consisting of the first thirty-three residues of HsTP is structurally missing, possibly due to its large intrinsic flexibility which can result in low electron density as has been shown in other studies (Palamini et al., 2016). The first, structurally-visible residue of HsTP is Q35 which is located at the top of an uncapped α-helix (Figures 1A,B), whereas EcTP has a leucine (L3) at the respective structurally equivalent position and is lacking an unstructured domain at its N’-terminus (Figure 1B). Amino acid sequence alignments of family II eukaryotic and prokaryotic nucleoside phosphorylases revealed that, while eukaryotic species harbor a N’-terminus sequence of variable length which is very poorly conserved (part of which corresponds to the pro-peptide), the prokaryotic orthologs totally lack such a sequence (Supplementary Figure S4).

Thus, we reasoned that a direct expression comparison of HsTP against its *E. coli* ortholog EcTP could shed more light on the expression difficulties encountered with HsTP. The EcTP construct was designed and cloned in a manner similar to HsTP (i.e. codon optimized gene was cloned in pET28a with a His^6^-tag at the N’-terminus) (Figure 1D) using the predicted amino acid sequence encoded by the *deoA* gene from the K-12 strain as has been deposited in the EcoGene 3.0 database (Zhou and Rudd, 2013). Prior to the experimental expression assessment of EcTP, analysis of the translation initiation rate (TIR) for both constructs (HsTP^199^ & EcTP) using the algorithm developed by Salis lab (Salis et al., 2009; Salis, 2011) yielded identical TIR values (11,643 arbitrary units, Supplementary Table S1). This algorithm uses a statistical-thermodynamics-derived model to calculate the free energy of different interactions between the mRNA and the ribosome and the sum of all those binding free energies (shown in Supplementary Table S1 as ΔG_total_) determines how likely it is for the ribosome to bind to an mRNA molecule and initiate translation (the higher the translation rate in Supplementary Table S1, the more likely for the ribosome to initiate translation). Particularly, calculations have shown that the secondary structure of the mRNA plays a determining role in those interactions and, in turn, in the translation rate (Espah Borujeni et al., 2017). The modelled mRNA structures of HsTP^199^ and EcTP including the 5’-UTR and the first ten codons of each construct were predicted to be identical as both were cloned into the same plasmid (identical ribosome binding sites) harboring a His^6^-tag at the N’-terminus along with the same linkers. Thus, based on the TIR analysis very similar expression levels of HsTP^199^ and EcTP were anticipated. However, surprisingly, expression of EcTP in BL21 (DE3) under the same conditions used for HsTP^199^ yielded ∼100–120 mg of protein per L of culture medium (entire amount in the soluble fraction) which is a ∼28-fold larger quantity relative to HsTP^199^ (3–5 mg/L, Table 1). These findings led us to hypothesize that the primary and secondary structure of HsTP’s N’-terminal domain, could possibly impede efficient protein expression.

### N’-Terminal Truncation of HsTP Significantly Improves Its Expression in *E. Coli*

Primary and secondary protein structure can have a profound effect on the translation, as well as protein solubility and toxicity. For example, if the N-terminal region contains many prolines or positively charged amino acids, it can slow down ribosome translocation and elongation (lower protein synthesis and higher ribosome sequestration) (Wruck et al., 2017; Charneski and Hurst, 2013; Lu and Deutsch, 2014). Indeed, close inspection of the HsTP^199^ construct revealed that the first five residues at the N’-terminus include three proline residues i.e. M1-G2-A3-P4-P5-A6-P7 (Figure 1C, A11-P12-P13-A14-P15 based on the full-length numbering; G2 is a scar residue as a result of the cloning process using NcoI site for the cloning of all constructs in the present study). This could negatively affect co-translational folding and in turn, explain the presence of a considerable amount of HsTP in the insoluble fraction. In addition, the N’-terminal highly flexible and unstructured polypeptide fragment of HsTP^199^ spanning the range A11-K34 could further impede co-translational folding (Waudby et al., 2019; Ciryam et al., 2013).

**FIGURE 2:**
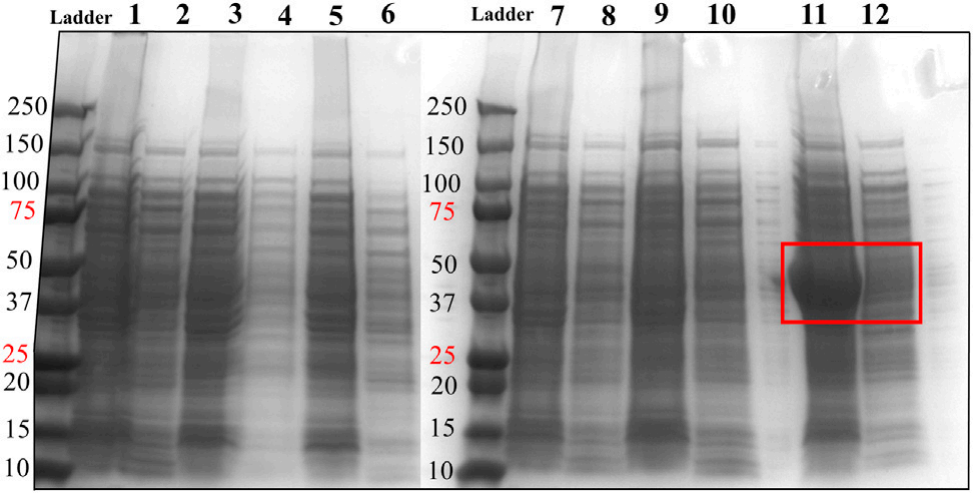
SDS-PAGE analysis of HsTP constructs. Lane 1: pET28a-HsTP^199^-whole cell lysate; Lane 2: pET28a-HsTP^199^-soluble fraction; lane 3: pJC20-HsTP^199^-whole cell lysate; lane 4: pJC20-HsTP^199^-soluble fraction; lane 5: pET28a-HsTP^215^-whole cell lysate; lane 6: pET28a-HsTP^215^-soluble fraction; lane 7: pET28a-HsTP^216^-whole cell lysate; lane 8: pET28a-HsTP^216^-soluble fraction; lane 9: pET28a-HsTP^217^-whole cell lysate; lane 10: pET28a-HsTP^217^-soluble fraction; lane 11: pET28a-HsTP^218^-whole cell lysate; lane 12: pET28a-HsTP^218^-soluble fraction. Red square in lanes 11 and 12 shows the overexpression of pET28a-HsTP^218^ which is predominantly detected in the insoluble fraction. Expression of all constructs was induced with 0.5 mM IPTG in TB medium (at OD^600^∼ 1) followed by overnight incubation at 30°C. For more details about the expression conditions, see Methods section.

**FIGURE 3:**
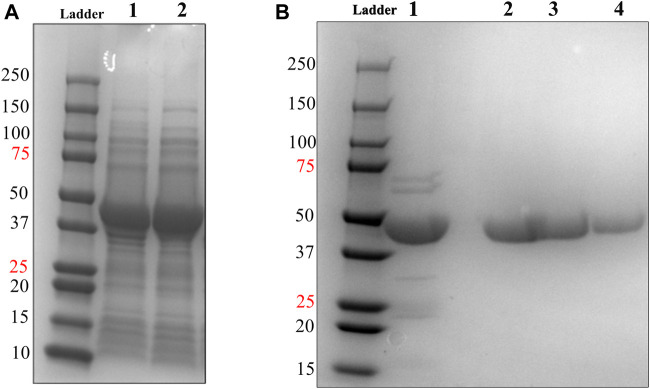
Soluble expression & 2-step purification of HsTP^218^. **(A)** Lanes 1 and 2 show the pET28a-HsTP^218^-whole cell lysate and pET28a-HsTP^218^-soluble fractions respectively expressed at 16°C **(B)** Lane 1 shows HsTP^218^ eluted from Ni^2+^ agarose beads and lanes 2, 3 and 4 display representative elutions from the Q-sepharose anion exchange chromatography step at 100, 125 and 150 mM NaCl respectively. ∼10 μg of protein was loaded in each lane.

We designed and generated HsTP truncated constructs (Figure 1D) within the first 33 residues (Figure 1C) which are not visible in its crystal structure (Figures 1A,B). Among the four new constructs tested, one (HsTP^218^) yielded large amounts of expressed protein, yet the protein was almost exclusively observed in the insoluble fraction when expressed at 30°C (Figure 2). Expression of HsTP^218^ under low temperatures (16°C and 22°C) and low IPTG conditions (Figure 3A; Table 1, Supplementary Figure S5) allowed the protein to fold properly and achieve soluble expression levels in a range more similar to those of EcTP (up to 70 mg/L, Table 1). The HsTP^218^ construct is missing the entire unstructured N’-terminal region comprising the first thirty-three amino acid residues of the full-length HsTP and structurally, it resembles the respective N’-terminus of EcTP. That is, downstream of the first K34 residue of HsTP^218^ the enzyme’s secondary structure forms an α-helix similar to EcTP (Figure 1B). Worth highlighting, the steady-state kinetic analysis of purified HsTP^218^ (Figure 3B) and HsTP^199^ (mature form of HsTP) showed very similar kinetic properties against dThd but a 2-fold increase in *k*_cat_/K_M_^dUrd^ in favor of HsTP^218^ (Table 2). In addition, analytical size exclusion chromatography (aSEC) confirmed the oligomeric state of HsTP^218^ exhibiting a dimer conformation (Supplementary Figure S6), which is in agreement with the active form of the enzyme as suggested by its crystal structure and previous studies (Norman et al., 2004).

**TABLE 2:** Steady-state kinetic parameters of HsTP^199^, HsTP^218^ and EcTP against dThd and dUrd. Assays were performed in assay buffer consisting of 20 mM Hepes, 50 mM KH_2_PO_4_, pH 7.5 at the indicated temperature. Parameters are shown as the best fit value ±standard error (Johnson, 2019) upon fitting the experimental data to Michaelis-Menten model (equation 1 in Methods section).

Enzyme species	dThd			dUrd			Conditions of steady-state kinetics
	*k*_cat_ (s^−1^)	K_M_ (μM)	*k*_cat_/K_M_ (M^−1^s^−1^)	*k*_cat_ (s^−1^)	K_M_ (μM)	*k*_cat_/K_M_ (M^−1^s^−1^)	
**HsTP** ^**199**^	5 ± 0.3	25 ± 5	(2 ± 0.4) × 10^5^	7.75 ± 0.3	180 ± 18	(4.3 ± 0.46) × 10^4^	Assay buffer, 25°C
**HsTP** ^**218**^	4.65 ± 0.1	28 ± 2.5	(1.66 ± 0.15) × 10^5^	4.4 ± 0.2	50 ± 8	(8.8 ± 1.4) × 10^4^	Assay buffer, 25°C
**EcTP**	402 ± 20	390 ± 50	(1 ± 0.15) × 10^6^	575 ± 24​	390 ± 36	(1.5 ± 0.15) × 10^6^	Assay buffer, 25°C

Interestingly, TIR analysis of HsTP^218^ revealed that this construct is characterized by the highest TIR (18,179 arbitrary units) among all the constructs tested in the present study (Supplementary Table S1). This high value of TIR is indicative of a high propensity for translation initiation as a result of the strong interactions between the HsTP^218^ mRNA’s secondary structure and the respective ribosomal binding sites essential for proper translation. Although the interactions between the mRNA and ribosomal RNA (that are governed and mediated by the mRNA’s structure) affect the TIR via a totally different mechanism than the protein’s primary and secondary structure at the N’-terminus, which was our initial hypothesis, HsTP^218^ expression may overall have been benefited by both mechanisms. That is, from one side, the first ten codons of the new HsTP^218^ truncated construct (His^6^-tag present at the C’-terminus) altered the secondary structure of its mRNA in a way that enhanced its binding to the ribosome and consequently the translation as evidenced by the higher TIR relative to the initial construct HsTP^199^. On the other hand, the lack of multiple successive proline residues in the new N’-terminus likely facilitated the co-translational folding and improved soluble expression. Of note, an analysis of 200 highly expressed endogenous *E. coli* proteins showed that the most abundant amino acid residue at the 3rd position of the polypeptide chain is lysine (Bivona et al., 2010), which is in agreement with the HsTP^218^’s sequence (M1-G2-**K3**-Q4---). HsTP^216^ and HsTP^217^ constructs appeared to have very similar TIRs, which is in-line with their low expression levels, but HsTP^216^ showed ∼4-fold lower soluble protein, possibly attributed to the successive proline residues at its N’-terminus that could stall translation (Starosta et al., 2014; Peil et al., 2013; Huter et al., 2017).

### PEGylation Optimization of HsTP

**FIGURE 4:**
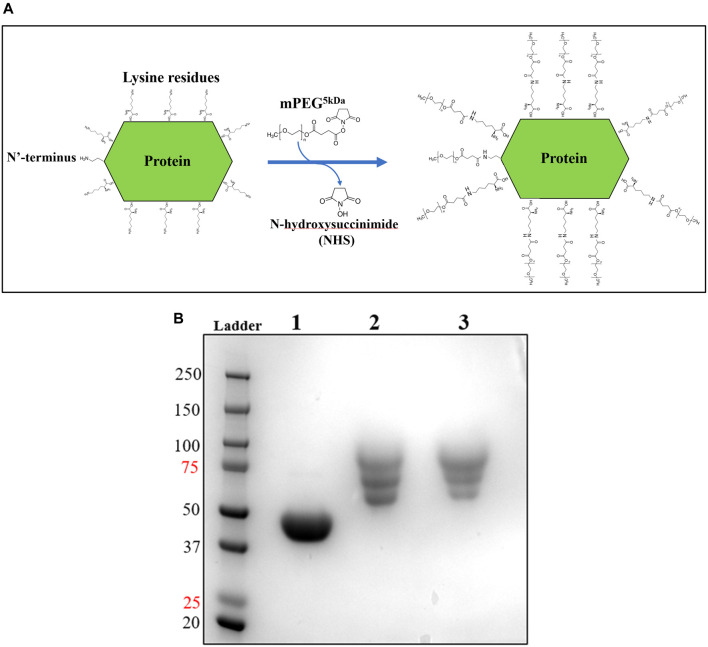
mPEG^5kDa^-conjugation reaction with HsTP^199^. **(A)** Scheme of the conjugation reaction of HsTP199 with mPEG^5kDa^. The N-hydroxysuccinimide ester (NHS ester) reactive group of mPEG^5kDa^ reacts with primary amines (lysine side group and N’-terminus amino-group) leading to the final conjugation adduct with methoxy-PEG and the simultaneous liberation of NHS. **(B)** Lane 1: Purified HsTP^199^ (2-step purification including IMAC and anion exchange as described in Methods section) used for PEGylation; lanes 2 and 3 show mPEG^5kDa^-conjugated HsTP^218^ at 1:20 and 1:30 protein:mPEG^5kDa^ ratio respectively. Each lane contains ∼15 μg of protein.

**FIGURE 5:**
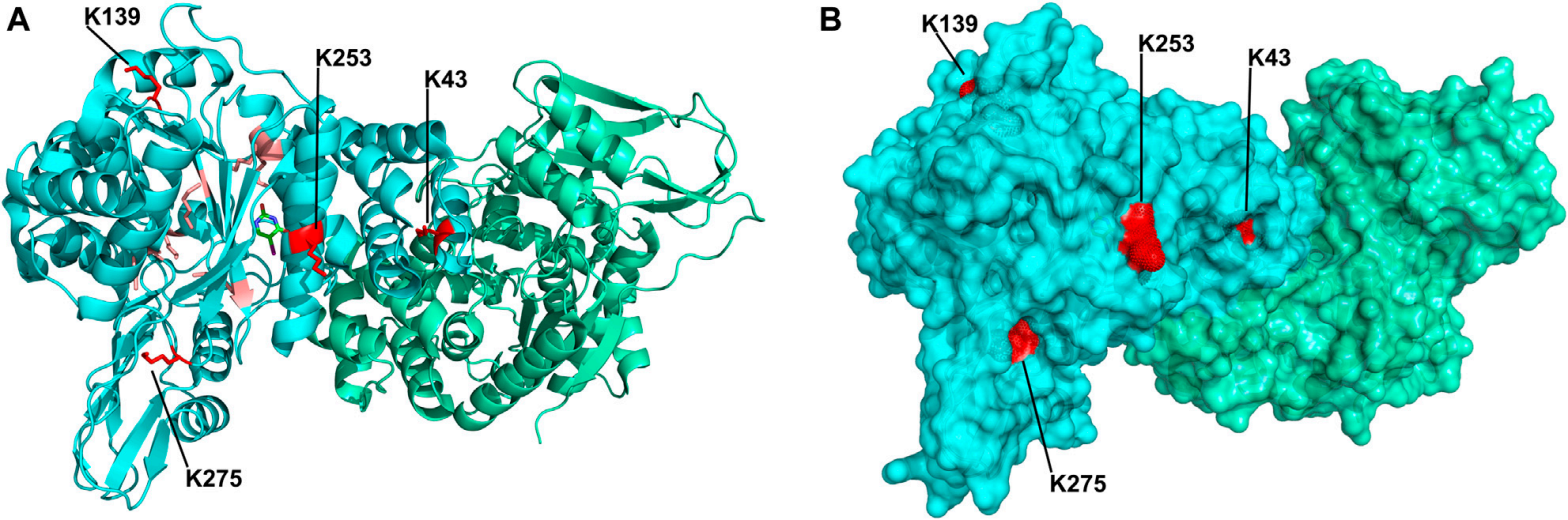
Crystal structure of HsTP homodimer (PDB: 2WK6). **(A)** Subunits A and B are shown in cyan and green respectively. All lysine residues of subunit A are shown as sticks. Buried lysine residues characterized by very low probability for PEGylation are colored as salmon red whereas four, more surface exposed lysine residues (K43, K139, K253, K275) with higher likelihood for conjugation are shown in red. **(B)** Surface representation of HsTP’s crystal structure. Monomers and lysine residues are colored as in panel A. K43, K139, K253 and K275 are labeled and their exposed molecular surface is shown in red dots. Structural representations were prepared using PyMol 2.4.1 (Schrödinger, LLC).

Upon improvement of the recombinant expression of HsTP, we undertook efforts to optimize its PEGylation. PEG has been increasingly used in the field of biologics during the recent decades as a time extension strategy aiming at the improvement of the thermodynamic stability (Santos et al., 2019; Moreno-Pérez et al., 2016; immunogenicity (Gefen et al., 2013) and the pharmacokinetic (PK) properties of therapeutic proteins (Harris et al., 2001; Hamidi et al., 2006). Initial efforts to conjugate HsTP^199^ with methoxy-5-kDa-PEG-succinimidyl-succinate (mPEG^5kDa^) targeting primary amines on the surface (primarily lysines and N’-terminus-amino group) resulted in an apparent heterogeneous mixture of three distinct PEGylated enzyme species (Figures 4A,B) indicative of an inefficient PEGylation reaction. Indeed, HsTP contains eleven lysine residues per monomer (Figure 5A), however analysis of its crystal structure revealed that only four (K43, K139, K253, K275) are adequately surface-exposed and thus accessible to react with mPEG^5kDa^ (Figure 5B). In fact, as shown in Figure 5B, perhaps the only lysine residue that can react with mPEG^5kDa^ with high certainty and efficiency due to its high solvent accessibility is K253. In contrast, K43, K139 and K275 are less exposed, thereby likely yielding a heterogeneous mixture of PEGylated species. Indeed, based on the PEGylation pattern shown in Figure 4B, up to five sites appear to be modified with mPEG^5kDa^ as this would result in a ∼25 kDa increase in the molecular weight (MW) yielding a total of ∼75 kDa per monomer (upper band in the gel). It appears reasonable to assume that those five sites consist of the four lysine residues K43, K139, K253 and K275 in addition to the N’-terminus. Further, upon PEGylation, HsTP^199^ exhibits a 2.8- and 2.2-fold decrease in *k*_cat_^dThd^ and *k*_cat_/K_M_^dThd^ respectively (Table 3) suggesting that chemical modification with mPEG^5kDa^ of one or more lysine residues, negatively affects the enzyme’s catalytic activity.

**TABLE 3:** dThd steady-state kinetic parameters of HsTP^199^, HsTP^218^, HsTP^240^ and HsTP^241^ prior and post-PEGylation. Conjugation reactions took place at either 1:10, 1:20 or 1:30 protein:PEG molar ratio as described in the Methods section. Parameters are shown as the best fit value ± standard error (Johnson, 2019) upon fitting the experimental data to Michaelis-Menten model (equation 1 in Methods section).

Enzyme species	dThd			Conditions of steady-state kinetics
	*k*_cat_ (s^−1^)	K_M_ (μM)	*k*_cat_/K_M_ (M^−1^s^−1^)	
HsTP^199^	5 ± 0.3	25 ± 5	(2 ± 0.4) × 10^5^	Assay buffer, 25°C
PEGylated HsTP^199^ (1:20)	3.75 ± 0.25	33 ± 9	(1.1 ± 0.3) × 10^5^	Assay buffer, 25°C
PEGylated HsTP^199^ (1:30)	1.8 ± 0.1	20 ± 8	(0.9 ± 0.36) × 10^5^	Assay buffer, 25°C
HsTP^218^	11.5 ± 0.5	65 ± 8	(1.7 ± 0.23) × 10^5^	Assay buffer, 32°C
PEGylated HsTP^218^ (1:10)	6.3 ± 0.35	52 ± 5	(1.2 ± 0.13) × 10^5^	Assay buffer, 32°C
PEGylated HsTP^218^ (1:20)	6 ± 0.2	65 ± 9	(0.9 ± 0.13) × 10^5^	Assay buffer, 32°C
HsTP^240^	11.5 ± 0.4	59 ± 7	(2 ± 0.25) × 10^5^	Assay buffer, 32°C
PEGylated HsTP^240^ (1:10)	9.3 ± 0.3	65 ± 6	(1.43 ± 0.14) × 10^5^	Assay buffer, 32°C
PEGylated HsTP^240^ (1:20)	10 ± 0.5	90 ± 10	(1 ± 0.135) × 10^5^	Assay buffer, 32°C
HsTP^241^	13 ± 0.35	74 ± 9	(1.75 ± 0.22) × 10^5^	Assay buffer, 32°C
PEGylated HsTP^241^ (1:10)	11.7 ± 0.55	95 ± 8	(1.23 ± 0.12) × 10^5^	Assay buffer, 32°C
PEGylated HsTP^241^ (1:20)	10 ± 0.3	67 ± 7	(1.5 ± 0.16) × 10^5^	Assay buffer, 32°C

**SCHEME 1:**
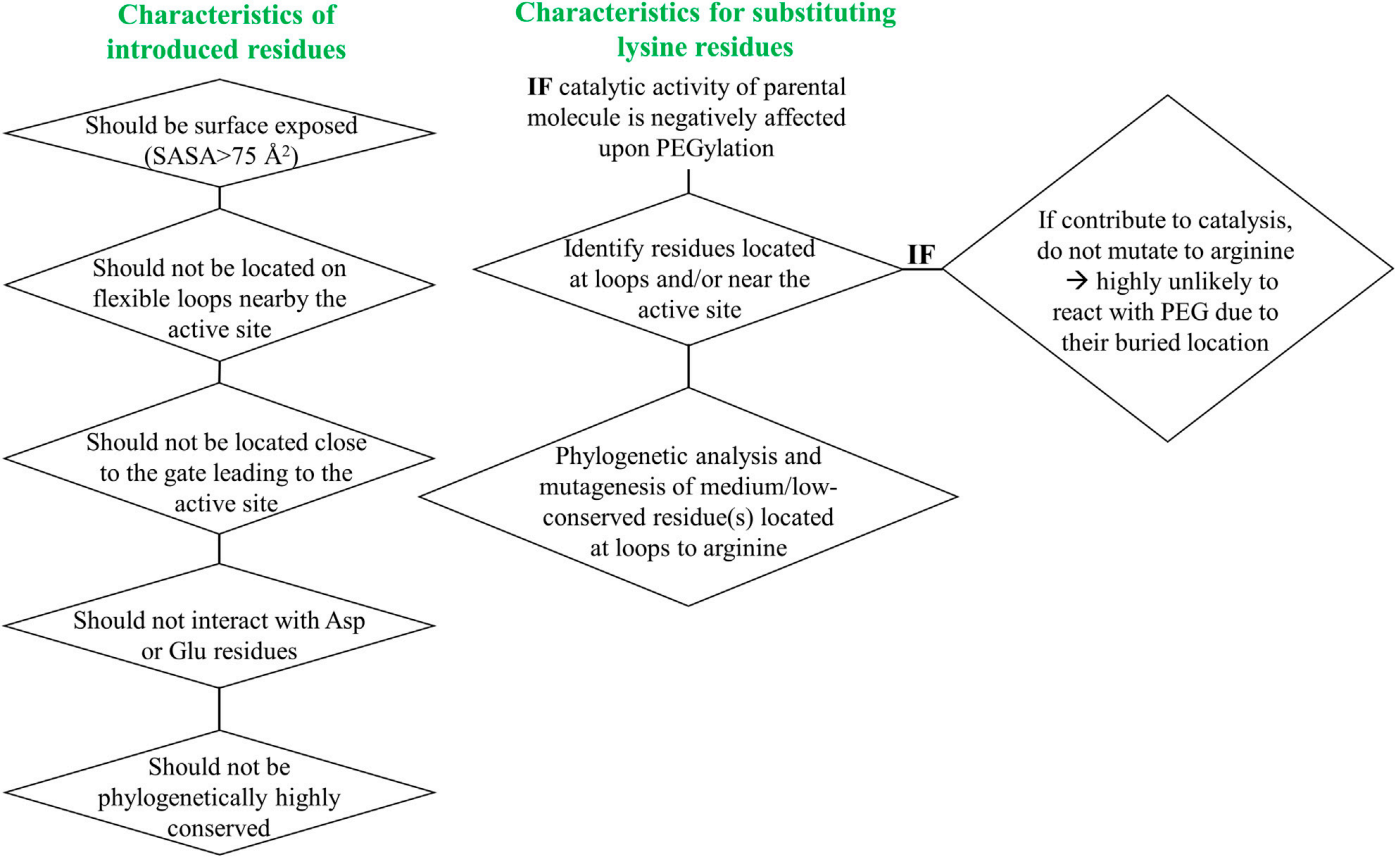
Engineer-in and engineer-out selection criteria for arginine and lysine residues respectively. SASA: solvent-accessible surface area.

**FIGURE 6:**
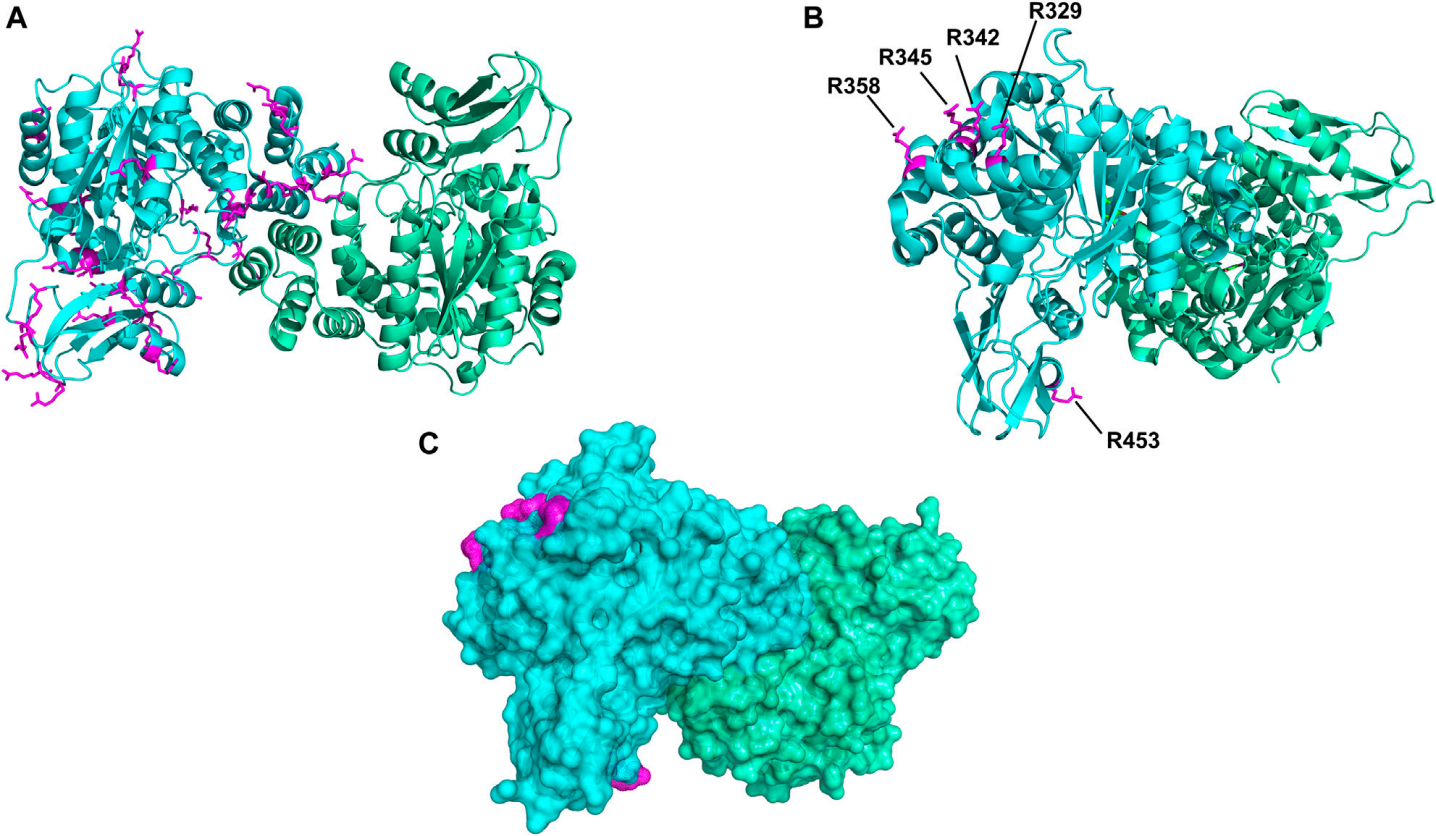
Spatial distribution of arginine residues mapped on the crystal structure of HsTP (PDB: 2WK6). **(A)** All arginine residues from subunit A (cyan color) are shown in magenta sticks. **(B)** Positions of arginine residues R329, R342, R345, R358 and R453 which satisfied our selection criteria and were substituted with lysine. **(C)** Surface representation of HsTP (at 20% transparency) highlighting the exposed molecular surface of the five arginine residues (magenta dots) shown in previous panel B. Structural representations were prepared using PyMol 2.4.1 (Schrödinger, LLC).

To address the limited PEGylation efficiency of HsTP and decrease in catalytic activity, we employed a rational surface engineering approach where arginine residues, located at surface-exposed positions suitable for PEGylation, were identified and were mutated to lysine. Conversely, lysine residues whose modification with mPEG^5kDa^ could hamper the catalytic activity were engineered-out and mutated to arginine. Relevant enzymology and enzyme-PEGylation literature was thoroughly reviewed, and the respective information was integrated into certain criteria (summarized in Scheme 1) that guided our engineering efforts. HsTP has thirty-two arginine residues widely distributed around its surface (Figure 6A). By analyzing and evaluating all arginine residues, we identified five residues (R329, R342, R345, R358, R453) that satisfied all our criteria and are shown in Figures 6B,C. These arginine residues 1) are not highly conserved among thymidine phosphorylases that belong to family II (Supplementary Figure S7A) and thus, are less likely to negatively impact the enzyme’s stability and catalytic activity, 2) they have very large solvent-accessible surface area (SASA) with the only exception being R342 which barely met our SASA criterion (>75 Å^2^, Supplementary Figure S7B), 3) they are not located on loops whose dynamics may be altered upon PEGylation and, in turn, negatively impact catalytic activity (rigidification issue (Rodríguez-Martínez et al., 2009)), 4) do not form salt bridges with aspartate or glutamate residues as this could alter the pKa of the newly introduced lysine residue, requiring the PEGylation reaction to be performed at highly alkaline pH and 5) they are not located nearby the active site and therefore, they do not prevent the substrate molecules from diffusing into it. Furthermore, analysis of the four lysine residues (K43, K139, K253, K275) based on our “engineer-out” criteria (Scheme 1) led us to mutate K139 and K275 to arginine and thus, prevent their PEGylation. Both lysine residues are located on flexible loops (flexibility was assessed by the B-factor values of those domains) which connect 2^nd^-shell domains nearby the active site (Figure 5A). Previous studies have shown that PEGylation of enzymes correlates with reduced structural dynamics and this in turn, may impair catalytic activity which can be directly dependent on conformational dynamics (Rodríguez-Martínez et al., 2008; Hsieh and Lin, 2015; Morgenstern et al., 2017; Santos et al., 2019). Of note, PEGylation of EcTP whose 8/22 lysine residues are located on surface-exposed flexible loops led to even greater reduction of its *k*_cat_^dThd^ relative to HsTP (400 s^−1^ vs 90 s^−1^ for un-PEGylated and PEGylated EcTP species respectively) (Supplementary Figure S8).

**FIGURE 7:**
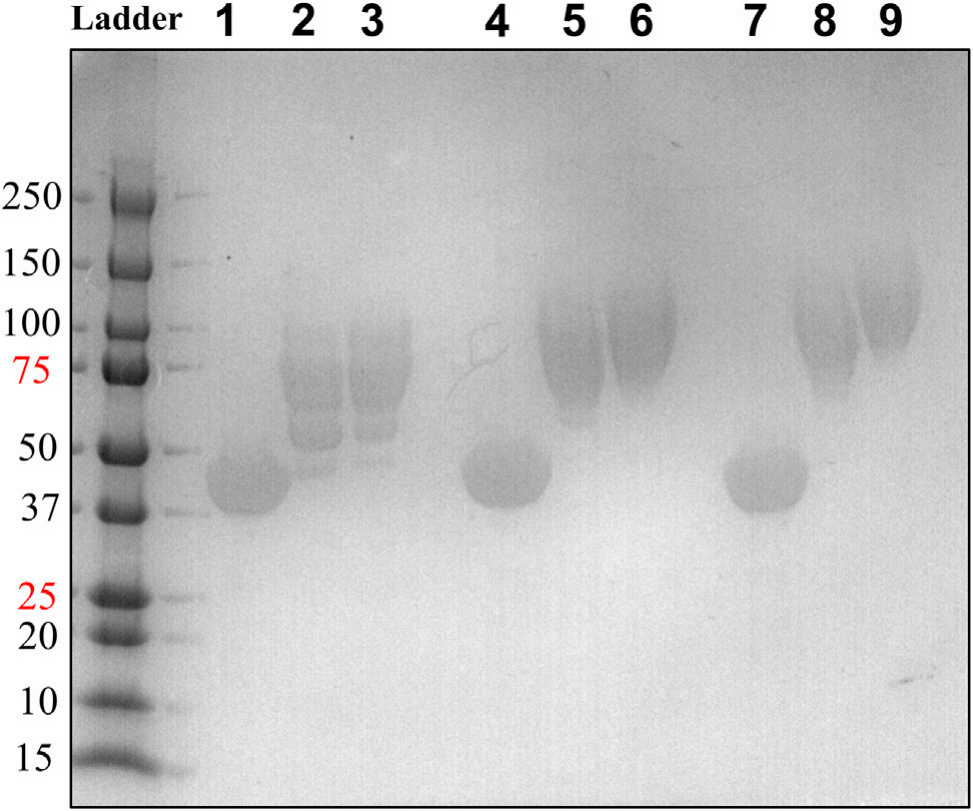
PEGylation efficiency assessment by SDS-PAGE of HsTP^218^, HsTP^240^ and HsTP^241^. Lanes 1-2-3, 4-5-6 and 7-8-9 show the HsTP^218^, HsTP^240^ and HsTP^241^ respectively prior to and after mPEG^5kDa^-conjugation at 1:10 and 1:20 protein:PEG molar ratio respectively. Approximately 15 μg of protein was loaded in each lane.

We generated two HsTP variants (HsTP^240^ and HsTP^241^) containing lysine-to-arginine and arginine-to-lysine substitutions using as a template the expression-optimized HsTP^218^ enzyme. HsTP^240^ contains K139R-K275R-R342K-R345K-R358K while HsTP^241^ has all seven identified mutations, K139R-K275R-R329K-R342K-R345K-R358K-R453K. PEGylation efficiency as well as steady-state kinetics were subsequently determined. Indeed, both HsTP^240^ and HsTP^241^ variants showed a significant improvement in PEGylation homogeneity relative to their parental HsTP^218^, with HsTP^241^ performing the best and clearly showing only one visible band around 100 kDa (Figure 7) when 1:20 (protein:PEG) molar ratio conjugation reaction conditions were used. In contrast, HsTP^218^ showed an almost identical profile for both PEGylation treatments tested (1:10 and 1:20 M ratio) which consists of five distinct bands on the SDS-PAGE (Figure 7) suggesting considerably lower conjugation efficiency. Very importantly, the steady-state kinetic characterization of HsTP^241^ revealed that the PEGylated enzyme retained it *k*_cat_/K_M_^dThd^ at the same levels as prior to PEGylation while PEGylation of HsTP^218^ caused a 2-fold decrease similar to what observed for HsTP^199^ (Table 3). Interestingly, upon PEGylation, the HsTP^240^ variant, which is missing the R329K and R453K substitutions, exhibited a decreased *k*_cat_/K_M_^dThd^. However, this decrease was due to an effect on K_M_ rather than on *k*_cat_ which remained almost unaltered relative to the unPEGylated species (Table 3). Taken together, our data demonstrate that by rational selection of suitable arginine and lysine residues for mutagenesis, PEGylation efficiency and consistency can be significantly improved while preserving the catalytic activity post-PEGylation. Our experimentally-validated rational PEGylation design may be applicable and benefit other protein and enzyme therapeutic molecules including asparaginase (Karamitros and Konrad, 2016; Meneguetti et al., 2019), cysteinase (Cramer et al., 2017) and kynureninase (Triplett et al., 2018; Karamitros et al., 2020).

## Discussion

Efficient recombinant production of biologics plays a crucial role in the viability of the drug development process. Availability of large quantities of a protein of interest facilitates the activities associated with all the stages of the drug development trajectory including biochemical and biophysical characterization, downstream processing, manufacturing and formulation optimizations, as well as pharmacological and toxicity assessments. Despite the immense progress in the field of molecular biology with the development of engineered *E. coli* strains and improved plasmids capable of expressing complex proteins of mammalian origins, the efficient expression of certain proteins can still be very challenging, and in turn, have a profound impact on cost and time. Thus, achieving high expression levels of high-quality protein that retains its biological activity (e.g., binding or catalytic properties) represents an important early focus in the field of biologics.

Similarly, the PEGylation of therapeutic proteins receives equal attention during the early phase of drug candidate selection and optimization. The conjugation of PEG to proteins has emerged as a powerful strategy to overcome significant limitations of protein pharmaceuticals such as sub-optimal *in-vivo* half-life and efficacy (Cheng et al., 2019), thermodynamic and serum instability (Lawrence and Price, 2016), and immunogenicity (Gefen et al., 2013). For example, there have been reported cases where protein PEGylation improved its pharmacokinetic properties by 4,200% (4 h vs 7 days for unPEGylated and PEGylated species respectively) (Holtsberg et al., 2002). Although alternative time-extension approaches have been recently developed (Despanie et al., 2016; Haeckel et al., 2016; Ding et al., 2014) and hold high promise, their wide clinical efficacy with multiple large biomacromolecules such as proteins has not yet been demonstrated. However, it needs to be stressed that as it has been demonstrated in some cases, PEG can be immunogenic leading to rapid blood clearance of the therapeutic protein due to the generation of anti-PEG antibodies (Yang and Lai, 2015). In those cases, further optimization of the PEGylation process (e.g., different type of PEG, sites of PEGylation) or alternative time extension strategies must be explored and evaluated. In addition, Nevertheless, the FDA has approved twenty-four PEGylated macromolecular drugs as of 2020, including six enzymes (adenosine deaminase, asparaginase, uricase, phenylalanine ammonia lyase). Therefore, PEGylation still represents a very attractive and potentially safe approach for the enhancement of the pharmacological features of therapeutic proteins, and particularly enzymes. However, the covalent attachment of PEG to the surface of enzymes (either targeting N-terminus, lysine or cysteine residues) often suffers by two main limitations: 1) poor PEGylation efficiency due to the low number of surface exposed residues, thereby resulting in a polydisperse population of species with distinct biochemical properties attributed to the different degrees of PEGylation that complicates the biochemical and biophysical characterization, and 2) conjugation of PEG can negatively impact the enzyme’s catalytic activity and thus, higher doses of a drug (i.e. higher cost) may be required to achieve a desirable pharmacological effect.

In the present study, we employed protein engineering strategies to optimize the bacterial expression and PEGylation of the human enzyme thymidine phosphorylase, an enzyme that could be used as a scaffold for the treatment of MNGIE, an inborn error of metabolism with high unmet medical need. While the mature HsTP displays very promising catalytic activity against the toxic metabolites which drive the MNGIE disease, its recombinant expression in *E. coli* was very poor, yielding ∼5 mg/L of culture medium. Multiple attempts to improve the expression of the enzyme by scouting different conditions, *E. coli* strains and plasmids failed to benefit protein production. Literature suggests that protein expression in *E. coli* can be governed by several underlying mechanisms with protein’s primary and secondary structure at the N’-terminus and mRNA’s secondary structure being the most important factors (Wruck et al., 2017; de Smit and van Duin, 1994). Careful inspection of the HsTP’s crystal structure revealed that the first thirty-four amino acid residues were missing from the crystal structure strongly suggesting that this domain is highly flexible and disordered. Of note, this domain includes a 10-residue pro-peptide (Figures 1C,D) with unknown function in the case of HsTP. While such pro-peptides have well-established inhibitory functions in other human enzymes like proteases (Boon et al., 2020), this did not appear to be the case for HsTP as the full-length species was active when assayed against dThd. In addition, the initially-tested HsTP^199^ (mature form of HsTP without the pro-peptide at the N’-terminus) carries three proline residues within the first five residues showing a APPAP motif which could have a detrimental effect on ribosomal translation causing strong stalling (Peil et al., 2013; Huter et al., 2017). Phylogenetic analysis of thymidine phosphorylase enzymes and direct comparison of HsTP against its bacterial ortholog EcTP, which expresses at very high levels, led to the design and generation of the HsTP^218^ construct that structurally resembles EcTP at the N-terminus and improved expression up to ∼14-fold. Interestingly, translation initiation rate (TIR) analysis suggested that the mRNA of HsTP^218^ binds more strongly to the ribosome than the other HsTP truncated constructs tested. Our findings suggest that sequence and structural differences among orthologs from different organisms can provide resourceful information that aid the rational engineering of desirable biochemical proteins including recombinant expression levels.

In this study we also proposed and experimentally validated a surface engineering strategy that enabled PEGylation improvement of HsTP at two different levels. Through rationally-designed arginine-to-lysine substitutions we managed to significantly improve PEGylation efficiency and homogeneity using a protein:PEG molar ratio as low as 1:20. This can be very advantageous on multiple levels: 1) additional extensive ion exchange purification steps are not required to resolve and isolate the different enzyme PEG-mers which are generated upon partial PEGylation, 2) the more homogeneous PEGylated enzyme species allow for a more accurate biochemical assessment, 3) low protein:PEG molar ratios can have a hugely beneficial impact on the costly stage of manufacturing process and equally importantly 4) a homogeneously-PEGylated protein is likely to have more consistent pharmacodynamics effects as well as a safer profile. On a second level, by mutating lysine-to-arginine residues at specific locations, the catalytic activity of HsTP was not negatively impacted by PEGylation. Enzyme PEGylation can be accompanied by a loss in catalytic activity as it has been shown in several studies (Zaghmi et al., 2019; Harris and Chess, 2003; Rodríguez-Martínez et al., 2009). While it is a very challenging and complex task to determine the exact underlying molecular mechanisms for this activity loss, increasing experimental (Rodríguez-Martínez et al., 2008) and computational (Yang et al., 2011; Wilding et al., 2018) evidence suggest that the loss can be largely attributed to increased conformational rigidity and restricted dynamics upon PEGylation. Based on these observations, we hypothesized and showed that PEGylation of lysine residues located on loops can be responsible for such activity loss. Enzyme loops are usually characterized by increased conformational plasticity which can be critical for catalytic activity (Malabanan et al., 2010; Yu et al., 2017). By mutating two loop-located lysine to arginine residues, the catalytic activity of HsTP^218^ was retained at the same levels observed prior to PEGylation, thus further supporting our initial hypothesis. Nevertheless, PEG conjugation at highly flexible and unstructured loops may be associated with increased stability (Dozier and Distefano, 2015; Wilding et al., 2018; Lawrence and Price, 2016) and therefore, the PEGylation goals must be assessed and defined on a case-specific manner. Taken together, our proposed strategies provide an effective way to optimize enzyme PEGylation and *E. coli* recombinant expression and are likely applicable for other proteins and enzymes for both therapeutic and industrial applications.

## Data Availability

The original contributions presented in the study are included in the article/Supplementary Material, further inquiries can be directed to the corresponding authors.
