# Erratum: Engineering of the Recombinant Expression and PEGylation Efficiency of the Therapeutic Enzyme Human Thymidine Phosphorylase

**DOI:** 10.3389/fbioe.2022.878674

**Published:** 2022-03-11

**Authors:** Frontiers Production Office

**Affiliations:** Frontiers Media SA, Lausanne, Switzerland

**Keywords:** enzyme engineering, catalytic activity, recombinant expression, PEGylation, thymidine phosphorylase

Due to a production error, the **Materials and Methods** section of the article was erroneously removed. The section appears below.

## Materials and Methods

### Cloning of HsTP and EcTP Genes

Amino acid sequences for wild-type HsTP and EcTP were codon-optimized for *E. coli* using the online tool provided by Integrated DNA Technologies (https://www.idtdna.com/pages/tools/codon-optimization-tool), and gene blocks with the resultant sequences were used as a template for polymerase chain reaction (PCR) amplification. Primers were designed to amplify the HsTP and EcTP gene fragments as follows: the 5′ primer introduced a His^6^-tag at the N-terminus and an NcoI restriction site; the 3′ primer introduced an EcoRI restriction site. PCR amplification was carried out following the Kapa HiFi protocol (Roche) with an annealing temperature of 60°C and a 2-min extension at 72°C for a total of 25 cycles. The amplicons were digested with NcoI and EcoRI, gel-purified, ligated into pET28a (Novagen) with T4 DNA ligase, and transformed into MC1061 cells (Lucigen). Primers used to generate the variants in this study are provided in **Supplementary Table S2**. Positive clones were screened by colony PCR using as the forward primer, one specific primer for the insert and as the reverse primer, one specific primer for the plasmid backbone.

### Expression and Purification of HsTP and EcTP Constructs

#### Expression

HsTP and EcTP constructs either in BL21 (DE3) or C41 (DE3) cells were cultured in terrific broth (TB) media supplemented with 50 μg/ml of kanamycin at 37°C until OD at 600 nm reached between and 0.8 and 1 units when standard shake flasks were used, or OD of 5 when ultra-yield flasks (Thompson) were used. Then, for HsTP^199^ and EcTP, 0.5 mM IPTG was added, temperature was reduced to 30°C, and expression was allowed to proceed for 20–22 h. Expression-optimized variants, including HsTP^218^, were induced with 0.1 mM IPTG and expressed at 16°C for 40 h.

#### Purification by Immobilized Metal Affinity Chromatography

Cells were pelleted by centrifugation at 4°C and resuspended in lysis buffer consisting of 50 mM Na_2_HPO_4_, 300 mM NaCl, 10 mM imidazole, 1 mM PMSF protease inhibitors, and 1 μl of 1 mg/ml DNase I for each ml of cell suspension, at pH 8. Resuspended samples were kept on ice and lysed by sonication. After sonication, the samples were centrifuged at 12,000 ×g for 1 h at 4°C, the supernatant was decanted, filtered through a 0.2 μm filter, and the pellet discarded. Lysate was mixed with Ni-NTA resin (Qiagen) (Ni-NTA resin volume was in the range of 4–8 ml of slurry mixture depending on the expected protein amount in the cell lysate) which had been previously equilibrated with 20 bed volumes of lysis buffer (50 mM Na_2_HPO_4_, 300 mM NaCl, 10 mM Imidazole, pH 8). The lysate-resin mixture was incubated at 4°C for 2 h (batch purification) and subsequently was applied by gravity flow to a polypropylene column. The column was washed with 20 bed volumes of washing buffer (50 mM Na_2_HPO_4_, 300 mM NaCl, 20 mM Imidazole, pH 8) followed by elution with three bed volumes of elution buffer (50 mM Na_2_HPO_4_, 300 mM NaCl, 300 mM Imidazole, pH 8). The eluted protein was buffer-exchanged against 20 mM Tris-Cl and 20 mM NaCl, pH 7.5 using 10 kDa MWCO protein concentrator tubes (Amicon) and was subjected to ion-exchange chromatography described below.

#### Q-Sepharose Purification

Following buffer exchange, the IMAC-eluted protein was applied by gravity flow to a polypropylene column loaded with 2 ml of Q-FF resin which had been previously equilibrated with 20 bed volumes of binding and washing buffer (20 mM Tris-Cl, 20 mM NaCl, pH 7.5). The column was washed with 20 bed volumes of binding and washing buffer, then eluted with a 50–500 mM NaCl gradient. Purity of the protein elutions was assessed by SDS-PAGE, and the fractions with the highest concentration and purity were pooled. HsTP was observed to elute between 100 and 200 mM NaCl. Purified HsTP was aliquoted, mixed with 15% (v/v) final glycerol concentration, flash-frozen with liquid nitrogen, and stored at −80°C for future use.

#### Immunoblotting

Immunoblotting for the detection of expressed HsTP was carried out according to standard protocols (Slieman and Leheste, 2020). Briefly, protein concentration was calculated using NanoDrop (One microvolume UV-Vis spectrophotometer from Thermo Scientific) by recording the A^280^. For crude extracts, A^280^ of 1 was assumed to be equivalent to 1 mg/ml whereas for HsTP and EcTP, the respective theoretical molar extinction coefficient values (ε^HsTP^ = 23,490 M^−1^ cm^−1^and ε^EcTP^ = 24,410 M^−1^ cm^−1^ as calculated from Expasy’s protparam online tool based on the primary amino acid sequence of the proteins) were used for the conversion of absorbance to molar concentration. Typical protein amount loaded per well was in the range of 10–15 μg. The protein samples were analyzed by SDS-PAGE and subsequently, the separated proteins were transferred onto a nitrocellulose membrane in transfer buffer (25 mM Tris-Cl, 190 mM glycine, 0.1% SDS) at a fixed current of 10 mA overnight at 4°C. Membranes were blocked with 5% skim milk dissolved in TBST buffer for 2 h at room temperature, followed by incubation with monoclonal anti-His^6^ antibodies (Sigma-Aldrich, SAB2702218). Incubation with secondary goat anti-mouse HRP-linked antibodies (ThermoFisher Scientific, G-21040) was carried out for 1 h at room temperature. Upon immunoblotting, bands were detected using an enhanced chemiluminescence (ECL) kit (SuperSignal West Pico PLUS from ThermoFisher Scientific, 34580).

#### Steady-State Kinetic Analysis of HsTP and EcTP

Steady-state kinetic characterization of HsTP and EcTP against dThd and dUrd was performed by continuously monitoring the decrease in absorbance of dThd and dUrd upon depyrimidination at 290 and 282 nm, respectively. Enzyme concentration in the range of 10–20 nM was used for all the steady-state kinetic measurements. Reactions took place in assay buffer consisting of 20 mM Hepes and 50 mM KH_2_PO_4_, pH 7.5 in a final volume of 1 ml placed in UV cuvettes with a pathlength of 1 cm. Prior to the addition of the enzyme, cuvettes were equilibrated at 37°C for 20 min using a heating block (VWR). The reaction progress was typically monitored for 2 min using a Jasco V750 spectrophotometer with a temperature-controlled cuvette holder and the absorbance was converted to concentration using the extinction coefficient of dThd and dUrd (Δε^290^ = 1,000 M^−1^ cm^−1^ and Δε^282^ = 1,370 M^−1^ cm^−1^) (Krenitsky et al., 1976). The obtained v/[E] (initial velocity/total enzyme concentration) values from the linear region of the reaction progress curves with <10% of substrate conversion were plotted against the respective substrate concentrations, and the steady-state kinetic parameters *k*_cat_ and *k*_cat_/K_M_ were calculated by nonlinear regression using the Michaelis−Menten model (Eq. 1 below) analyzed by the SoftZymics software (Igor Pro, Wavemetrics).

$$v=\left(kcat\;\times \left[S\right]\right)/\left(K_{M}+S\right)$$ (1)

#### Analytical Size-Exclusion Chromatography of HsTP

The purified protein samples were diluted in PBS buffer to a final concentration of approximately 2 mg/ml. A total of 2 µl of diluted samples was injected on a size exclusion column (TSKgel G4000SWxl, 7.8 × 300 mm, 8 µ particle size, from Tosoh). Isocratic elution was performed at a flow rate of 0.3 ml/min, using a Thermo ultimate HPLC system. The mobile phase contained 90% of phosphate buffer (50 mM sodium phosphate, 200 mM sodium chloride, adjusted to pH 7.2) and 10% ethanol. The column eluent was monitored by UV detection at 214 nm. The BioRad gel filtration standard was used as molecular weight markers.

#### Phylogenetic Analysis of Thymidine Phosphorylase Enzymes

Phylogenetic analysis was performed by using the program Geneious prime. The full-length amino acid sequences of HsTP and EcTP deposited in the Uniprot database (P19971 and P07650) were used as query sequences to perform massive BLAST alignments and thus, determine conserved residues. Geneious is directly connected to the NCBI database and searches for homologous sequences to the query entry. For restricting the search exclusively for either eukaryotic or prokaryotic thymidine phosphorylase enzymes, the commands “Eukaryotes [organism]” or “Prokaryotes [organism]” were used in the “entrez query” option respectively. The maximum number of thymidine phosphorylase hits to be searched for from the NCBI database was set to 1,000.

#### Translation Initiation Rate *In-Silico* Analysis

Translation initiation rates (TIR) were calculated using the online webserver De Novo DNA at the following URL: https://salislab.net/software/. The mRNA of each construct starting from the transcriptional start site and ending at the transcriptional terminator sequence was subjected to analysis based on the ribosome binding site (RBS) predict-mode algorithm using *E. coli* as the host organism for recombinant expression. The RBS calculator in the predict-mode identifies all the starting codons in the mRNA sequence and calculates a translation initiation rate (TIR) in arbitrary units (a.u.; the larger the value, the higher the TIR) for each of them according to its statistical thermodynamics algorithm as described elsewhere (Salis et al., 2009). The TIR calculations are accompanied by ribosome binding Gibbs free energy calculations that represent the quantification of the interaction between the ribosome and the mRNA that, in turn, affects the TIR. The reported ΔG_total_ is defined as follows: ΔG_total_ = ΔG_mRNA-rRNA_ + ΔG_spacing_ + ΔG_start_ − ΔG_standby_ − ΔG_mRNA,_ where ΔG_mRNA-rRNA_ is the energy released when the last nine nucleotides of the *E. coli* 16S rRNA hybridizes and co-folds to the mRNA sub-sequence. ΔG_start_ is the energy released when the start codon hybridizes to the initiating tRNA anticodon loop. ΔG_spacing_ is the free energy cost caused by a non-optimal physical distance between the 16S rRNA binding site and the start codon. ΔG_mRNA_ is the work required to unfold the mRNA sub-sequence when it folds to its most stable secondary structure and ΔG_standby_ is the work required to unfold any secondary structures sequestering the standby site after the 30S complex assembly.

#### Site-Directed Mutagenesis and Generation of PEGylation Variants

For the generation of the PEGylation variants HsTP^240^ and HsTP^241^ site-directed mutagenesis was performed following the overlap extension PCR methodology (Nelson and Fitch, 2011). Briefly, this method comprises three successive amplification steps and involves four primers as follows: two external ones that cover the 5′- and 3′- ends of the parental sequence and two additional ones which carry the desired mutations to be incorporated in the final sequence. Two independent PCR reactions (PCR1: forward external primer covering the 5′ combined with the reverse carrying the mutations and PCR2: reverse external at the 3′ combined with forward carrying the mutations) were performed to amplify two fragments which overlap at the regions flanking the mismatches. The two amplified fragments were agarose gel-purified and in a final third step, they were combined in equal molar quantities with the initial external primers and were subjected to the last PCR reaction resulting in the final amplicon that carries the desired point mutations. The PCR amplicons along with the pET28a plasmid were digested overnight at 37°C with NcoI/EcoRI and ultimately were cloned using T4 DNA ligase. The incorporated mutations were sequence-verified by Sanger sequencing.

#### Preparation of PEGylated HsTP and EcTP

Conjugation reactions with methoxy-5-kDa-PEG-succinimidyl-succinate (mPEG^5 kDa^, NOF Corporation) were performed in 100 mM Na_2_HPO_4_, pH 8.5 using purified HsTP^199^ and EcTP as described above in the respective section. Final enzyme concentration in the reaction was ∼115 μM and depending on the protein: PEG ratio, the respective concentration of PEG was adjusted accordingly: 1,150, 2,300, and 3,450 μM for 1:10, 1:20, and 1:30 M ratio, respectively. The lyophilized PEG was weighed and was added directly into a 2 ml tube containing either purified HsTP^199^ or EcTP at a volume of 1 ml. Immediately after the addition of the PEG, the tube was vortexed continuously for 30 s, followed by incubation at room temperature for 30 min under rotating conditions. Subsequently, the mixture was exhaustively buffer-exchanged to remove excess of unreactive PEG using protein concentrators with a 50-kDa MWCO (Thermo Scientific). HsTP^240^ and HsTP^241^ PEGylation variants and HsTP^218^ were conjugated following the same process except for the buffer, which was 50 mM Na_2_HPO_4_ and 50 mM NaCl, pH 8.0, and the final enzyme concentration was 200 μM. Following the final buffer exchange step, PEGylation homogeneity was assessed by SDS-PAGE and steady-state kinetics was performed using dThd as the substrate. PEGylated enzymes were buffer-exchanged against 50 mM Na_2_HPO_4_ and 50 mM NaCl, pH 7.0, mixed with a 15% final concentration of glycerol, flash-frozen in liquid nitrogen, and stored at −80°C.

The publisher apologizes for this mistake. The original article has been updated.

