# Supplementary figures and images for: Engineering of the Recombinant Expression and PEGylation Efficiency of the Therapeutic Enzyme Human Thymidine Phosphorylase
Source: Front Bioeng Biotechnol. 2021 Dec 17;9:793985. doi: 10.3389/fbioe.2021.793985 (PMC8718881; doi:10.3389/fbioe.2021.793985)

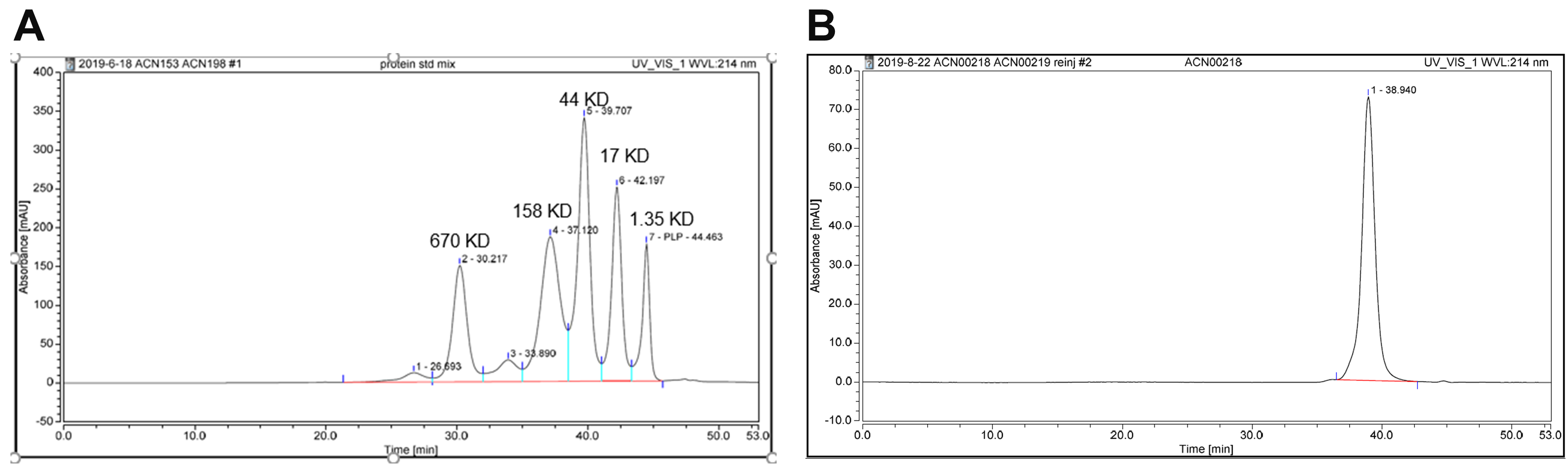

Supplement: Supplementary file 1 [file Image6.tif]

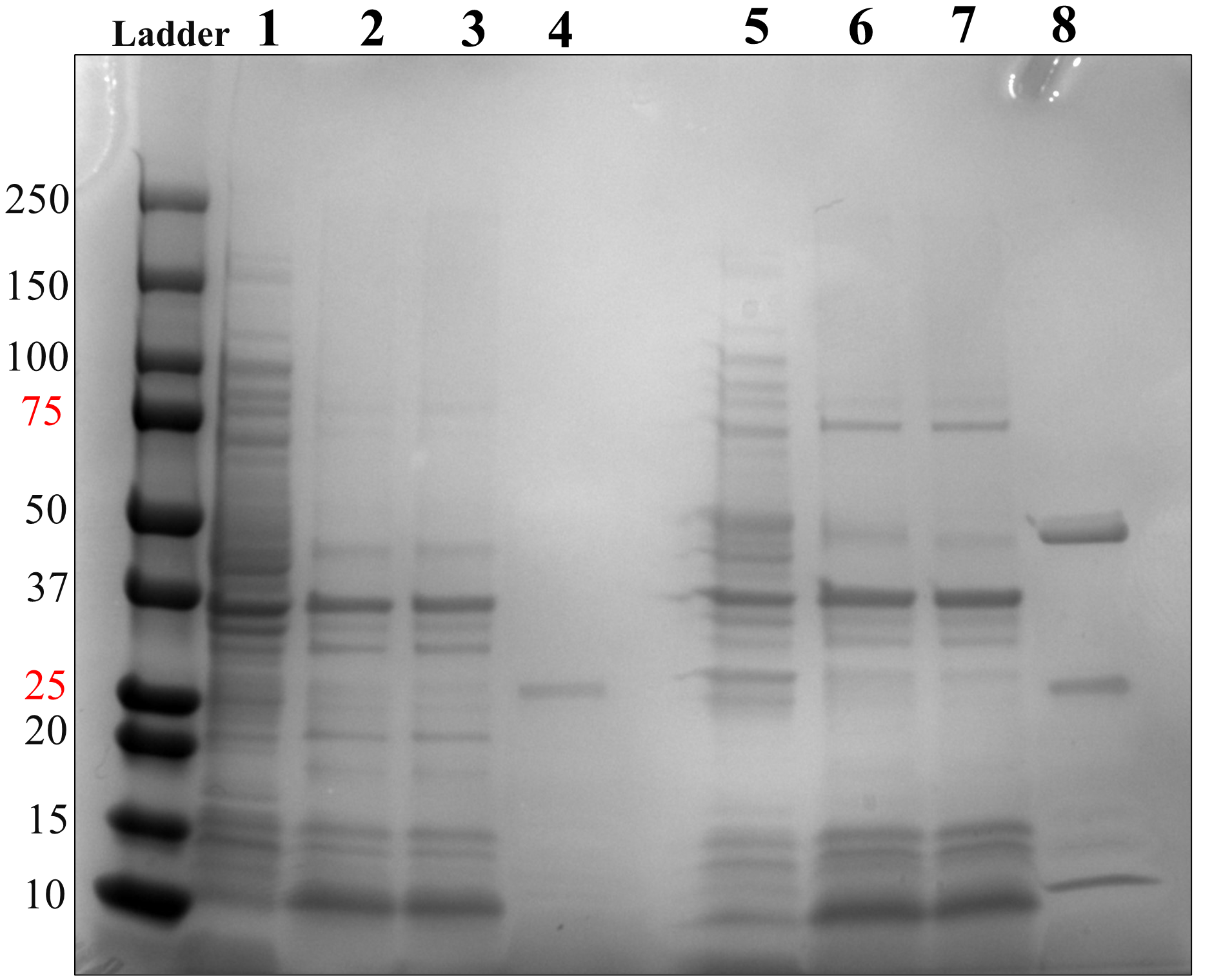

Supplement: Supplementary file 4 [file Image3.tif]

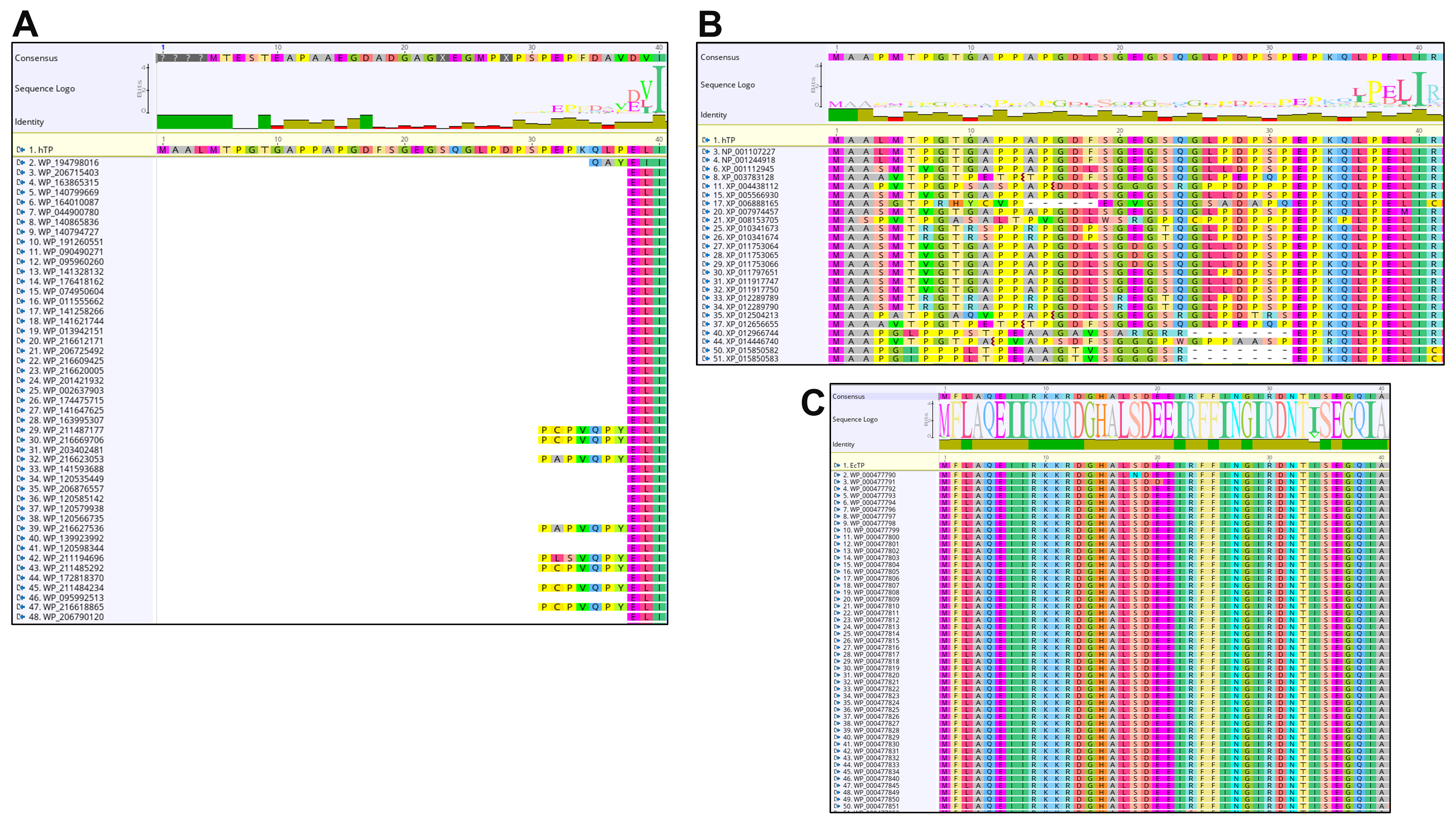

Supplement: Supplementary file 5 [file Image4.tif]

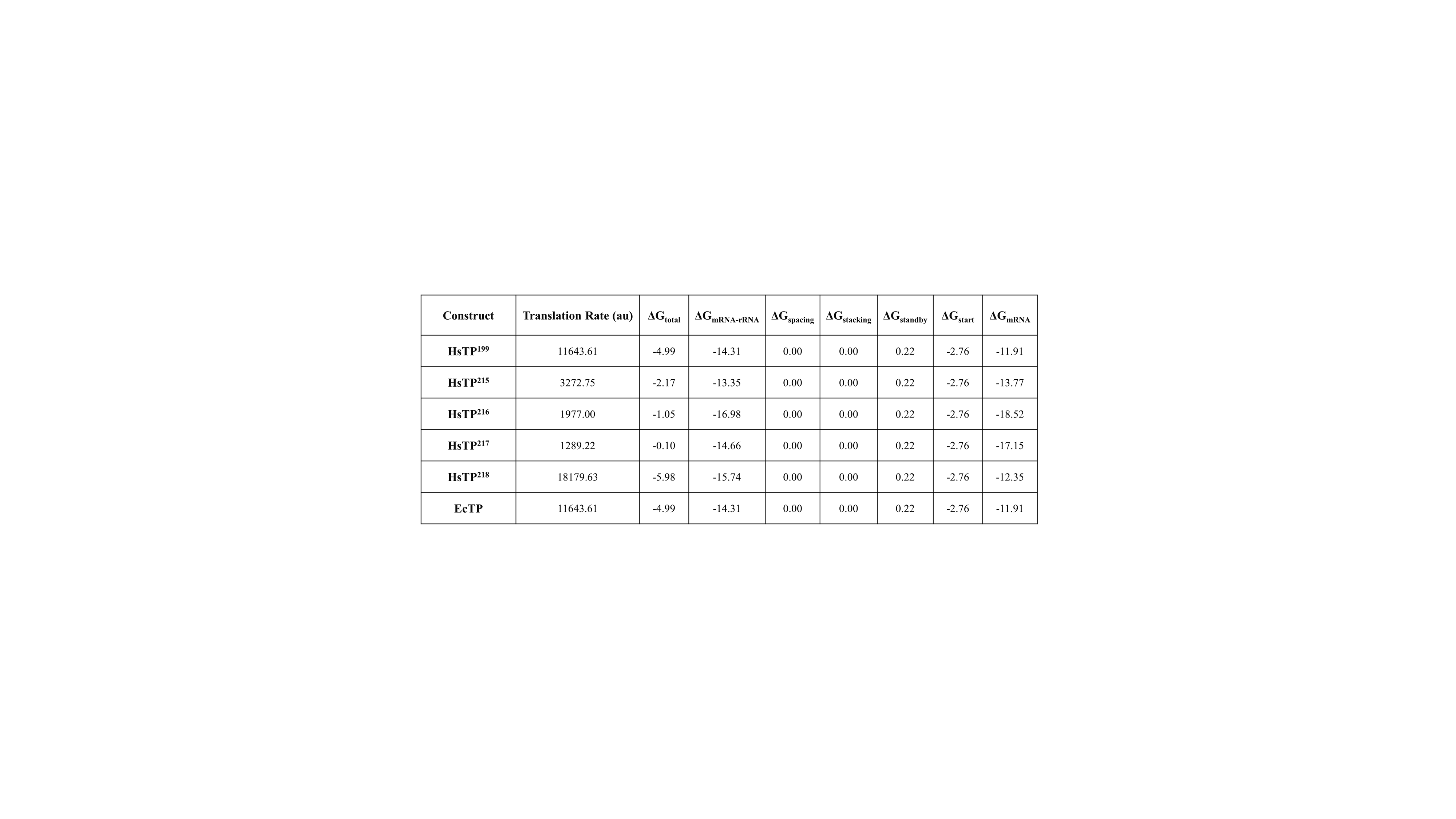

Supplement: Supplementary file 6 [file Image9.TIF]

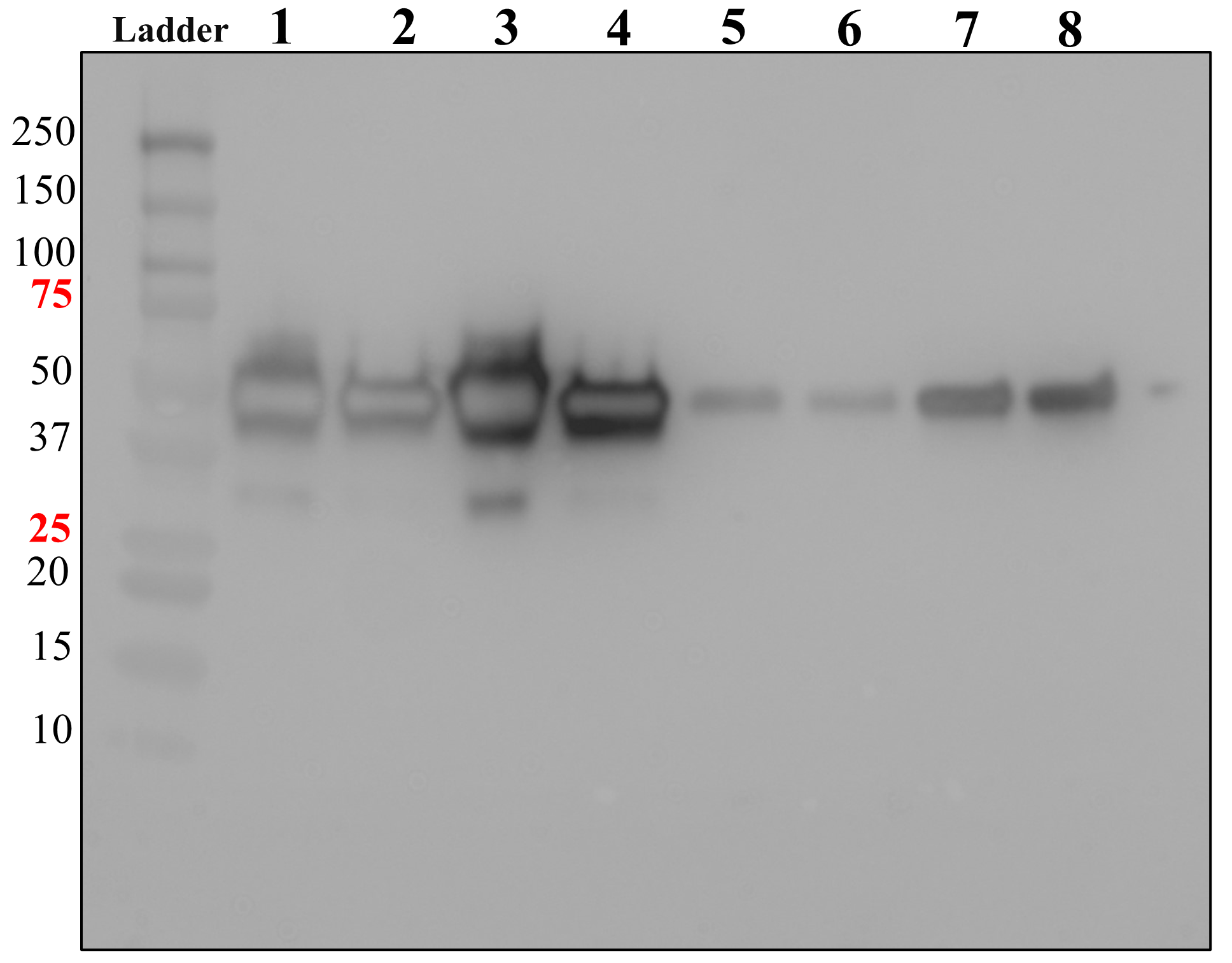

Supplement: Supplementary file 7 [file Image2.tif]

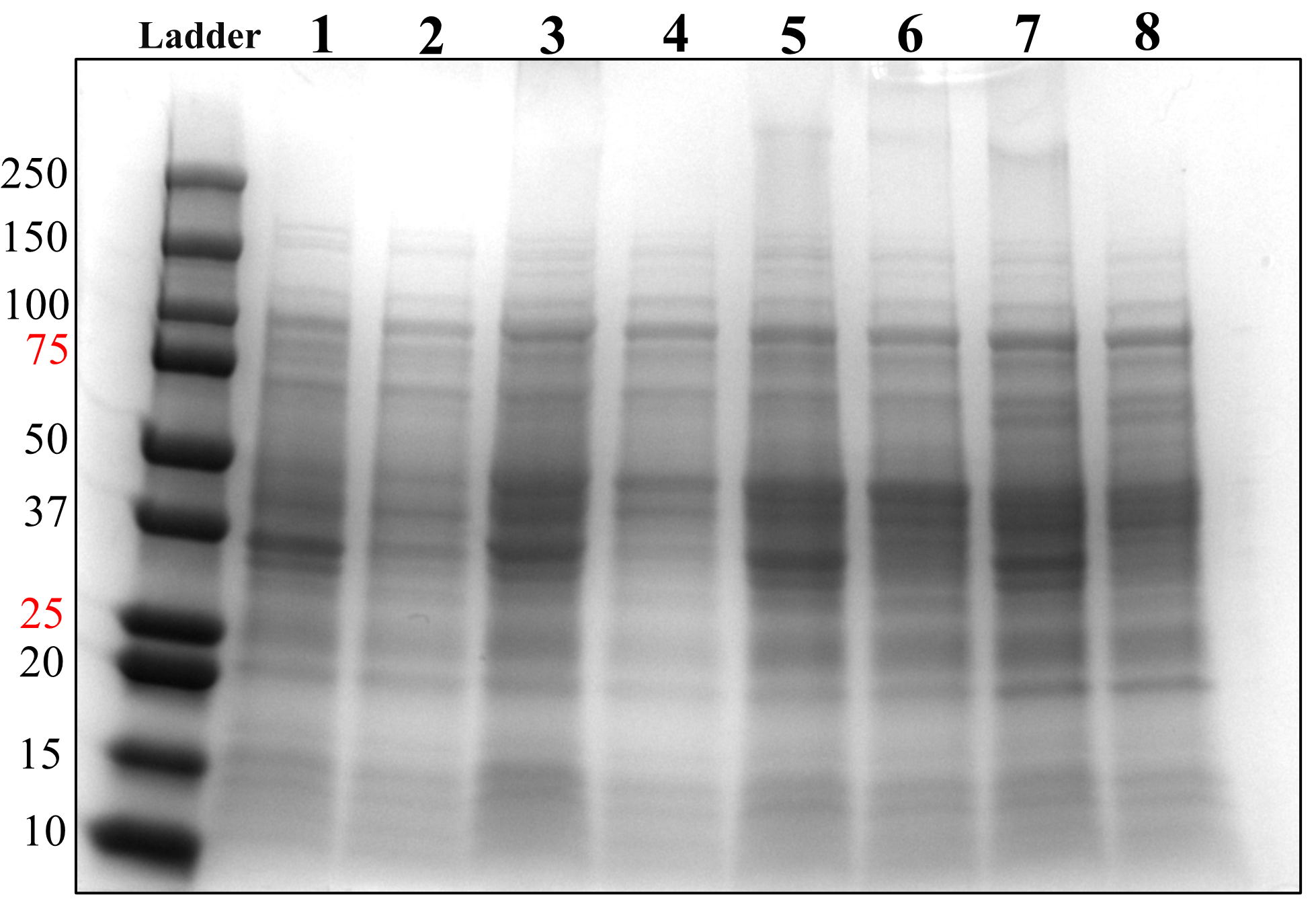

Supplement: Supplementary file 8 [file Image1.tif]

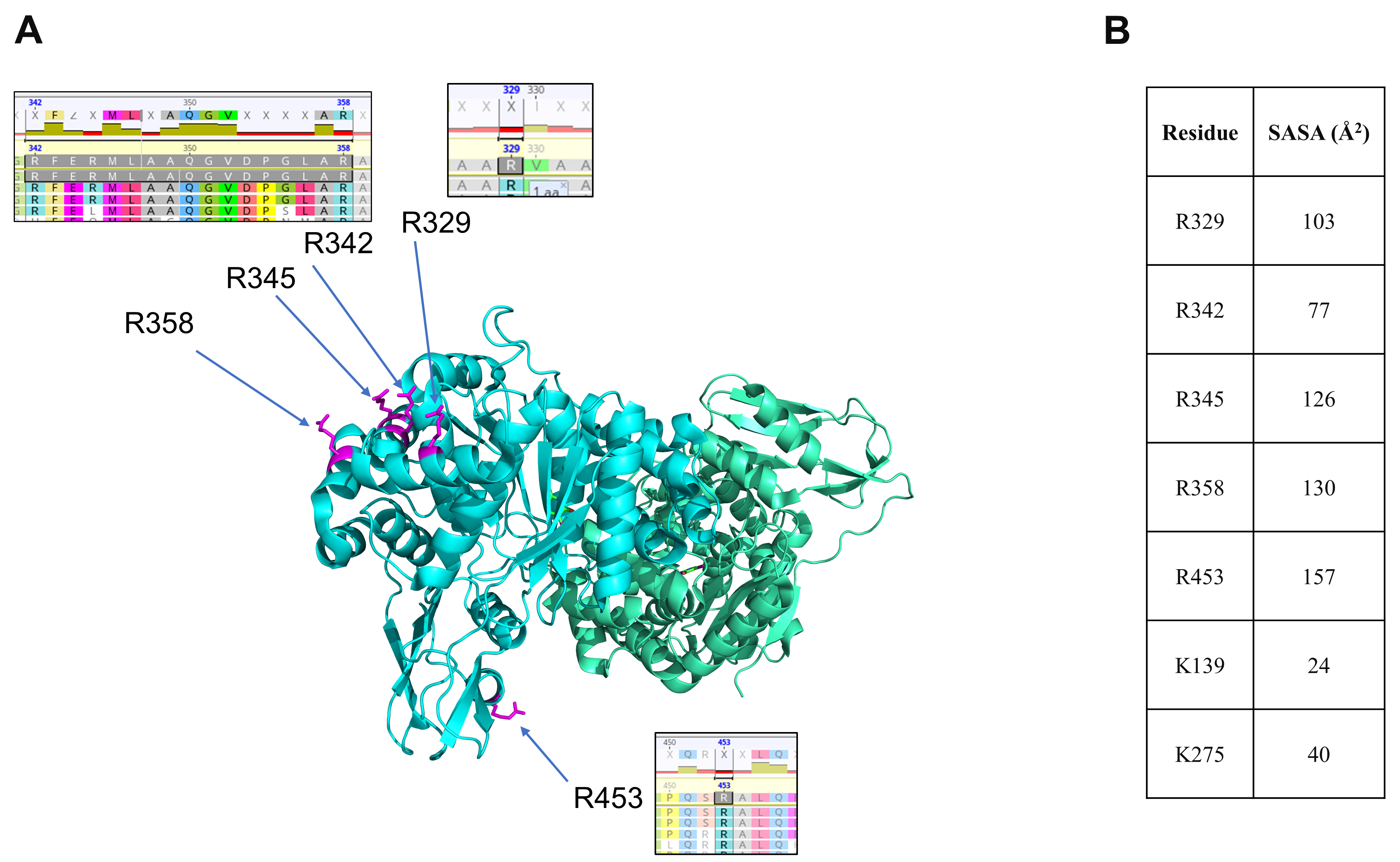

Supplement: Supplementary file 9 [file Image7.tif]

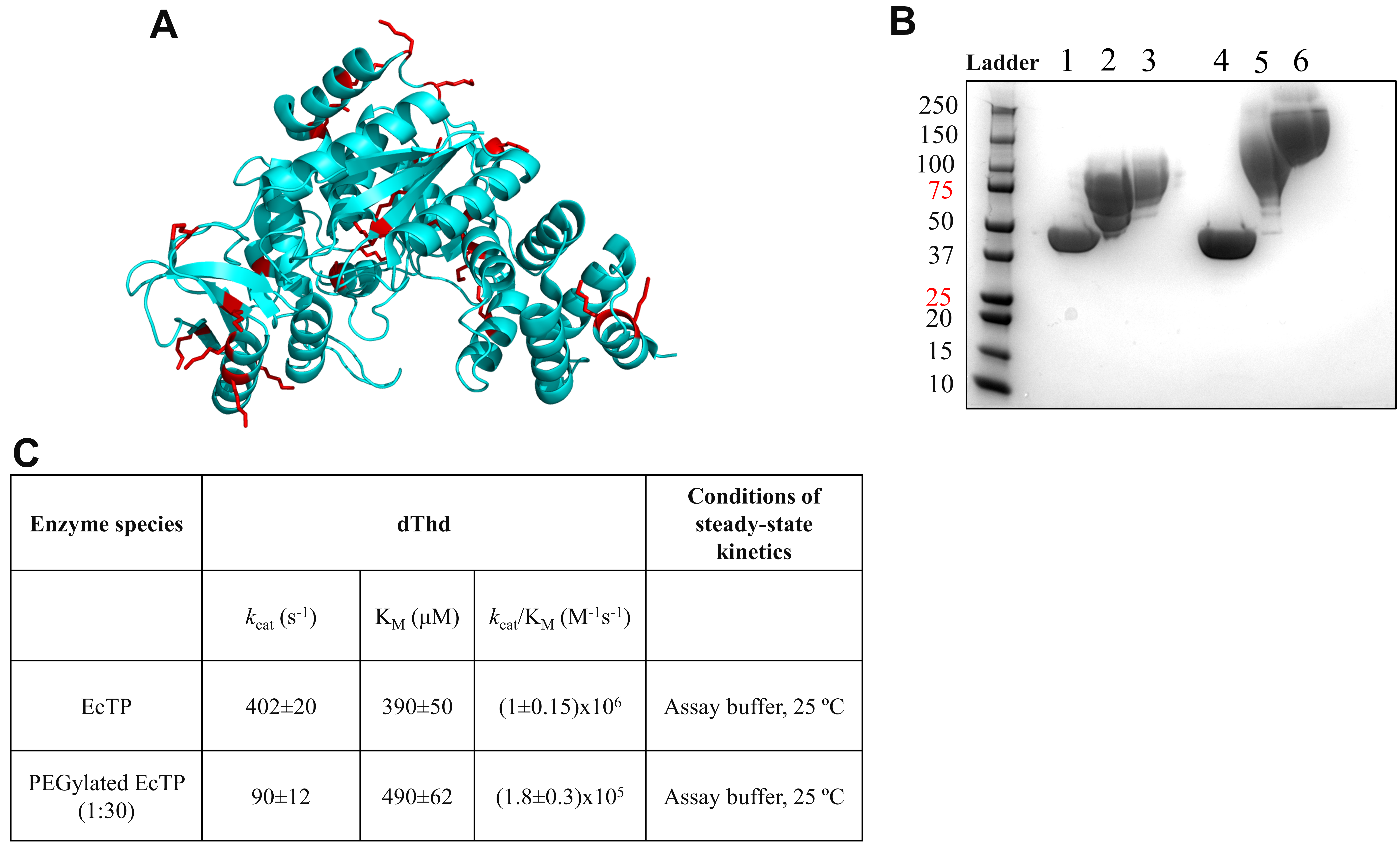

Supplement: Supplementary file 11 [file Image8.tif]

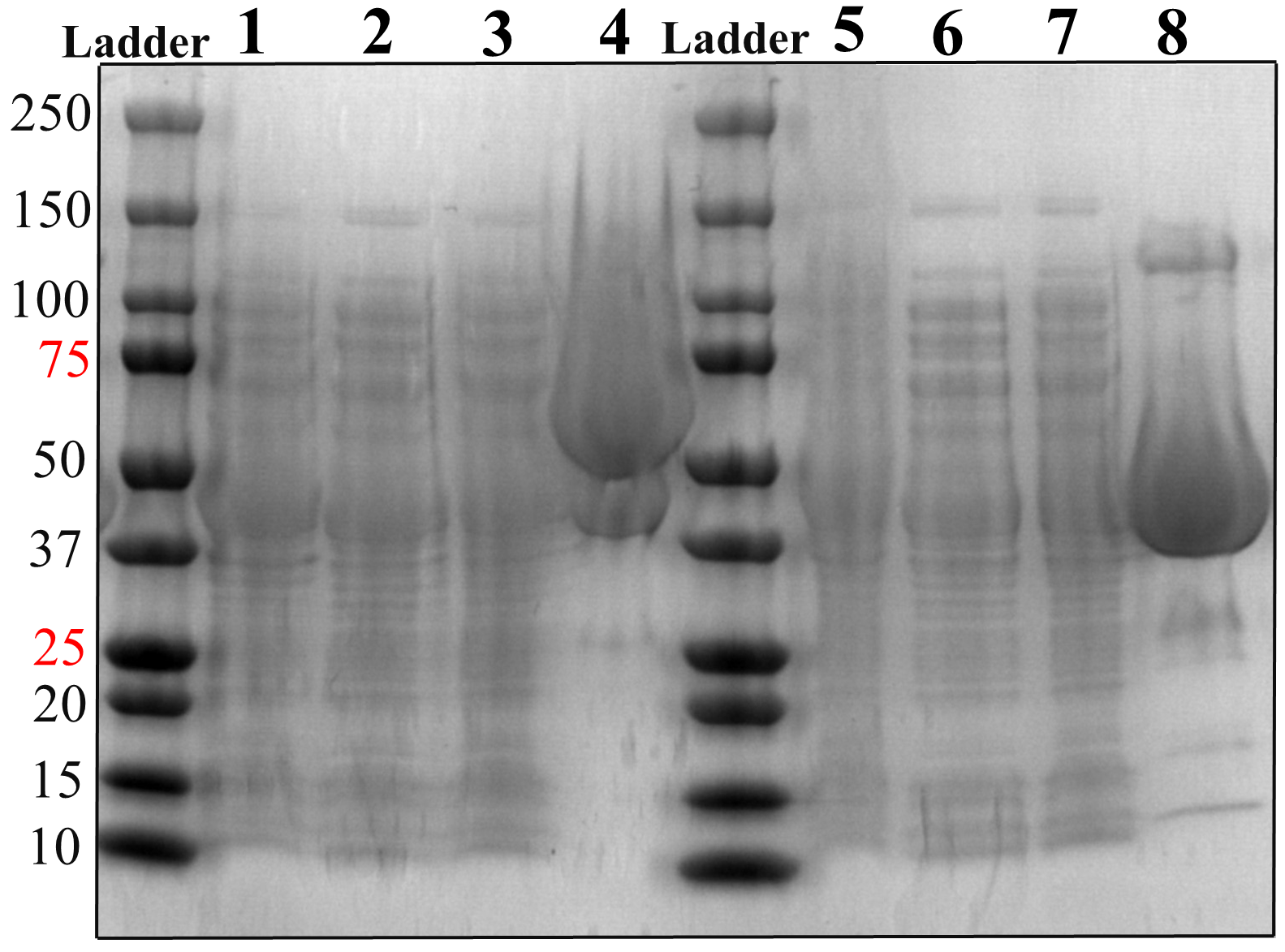

Supplement: Supplementary file 12 [file Image5.tif]
