# Supplementary material for: Engineering of the Recombinant Expression and PEGylation Efficiency of the Therapeutic Enzyme Human Thymidine Phosphorylase
Source: Front Bioeng Biotechnol. 2021 Dec 17;9:793985. doi: 10.3389/fbioe.2021.793985 (PMC8718881; doi:10.3389/fbioe.2021.793985)
Supplement: Supplementary file 2 [file Table1.docx]

| **Construct** | **Translation Rate (au)** | **ΔG_total_** | **ΔG_mRNA-rRNA_** | **ΔG_spacing_** | **ΔG_stacking_** | **ΔG_standby_** | **ΔG_start_** | **ΔG_mRNA_** |
| --- | --- | --- | --- | --- | --- | --- | --- | --- |
| **HsTP^199^** | 11643.61 | -4.99 | -14.31 | 0.00 | 0.00 | 0.22 | -2.76 | -11.91 |
| **HsTP^215^** | 3272.75 | -2.17 | -13.35 | 0.00 | 0.00 | 0.22 | -2.76 | -13.77 |
| **HsTP^216^** | 1977.00 | -1.05 | -16.98 | 0.00 | 0.00 | 0.22 | -2.76 | -18.52 |
| **HsTP^217^** | 1289.22 | -0.10 | -14.66 | 0.00 | 0.00 | 0.22 | -2.76 | -17.15 |
| **HsTP^218^** | 18179.63 | -5.98 | -15.74 | 0.00 | 0.00 | 0.22 | -2.76 | -12.35 |
| **EcTP** | 11643.61 | -4.99 | -14.31 | 0.00 | 0.00 | 0.22 | -2.76 | -11.91 |
