# Supplementary material for: Engineering of the Recombinant Expression and PEGylation Efficiency of the Therapeutic Enzyme Human Thymidine Phosphorylase
Source: Front Bioeng Biotechnol. 2021 Dec 17;9:793985. doi: 10.3389/fbioe.2021.793985 (PMC8718881; doi:10.3389/fbioe.2021.793985)
Supplement: Supplementary file 3 [file Presentation1.PPTX]

## Slide 1
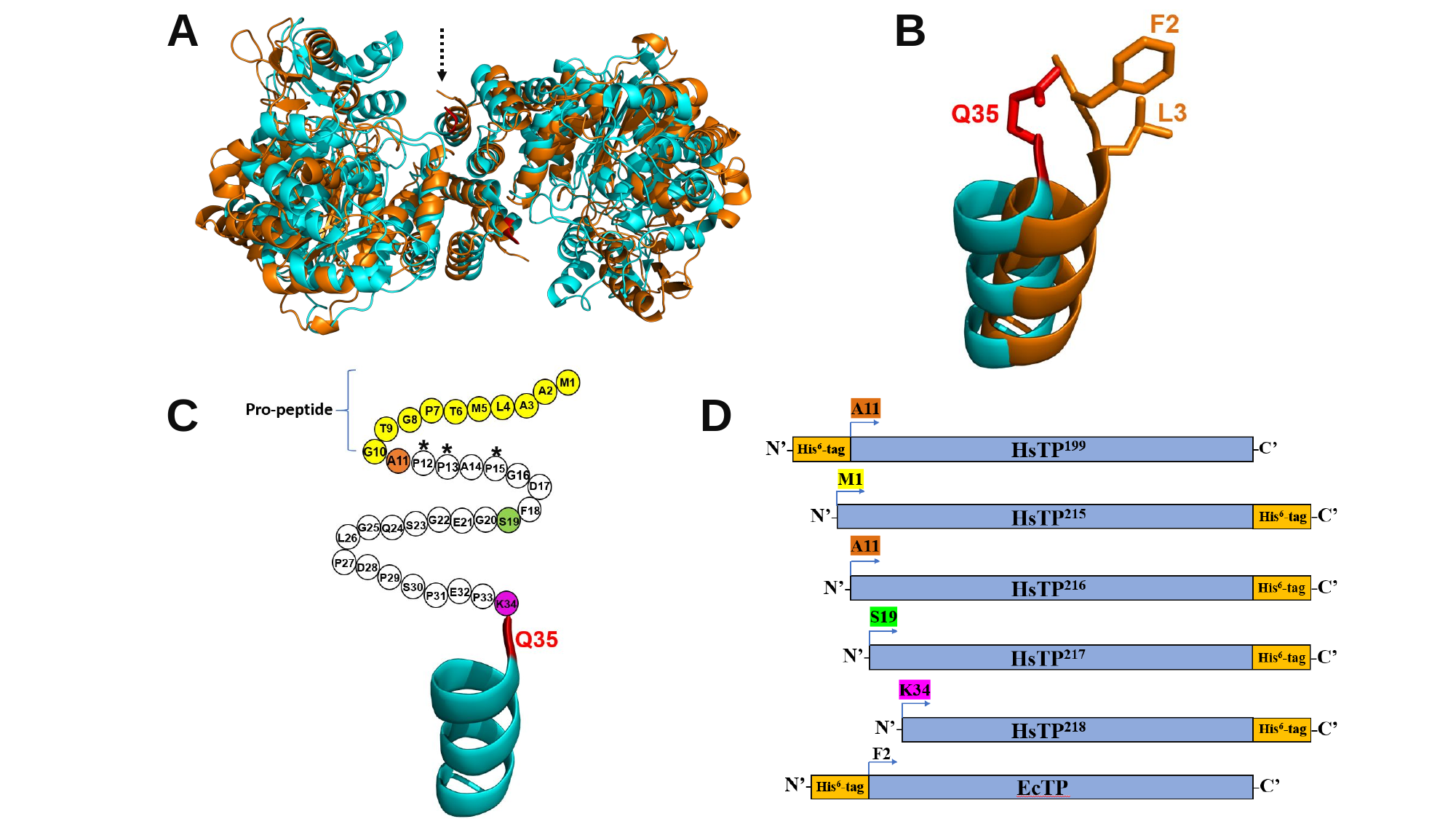

A
B
C
D
*
*
*
*
*
*

## Slide 2
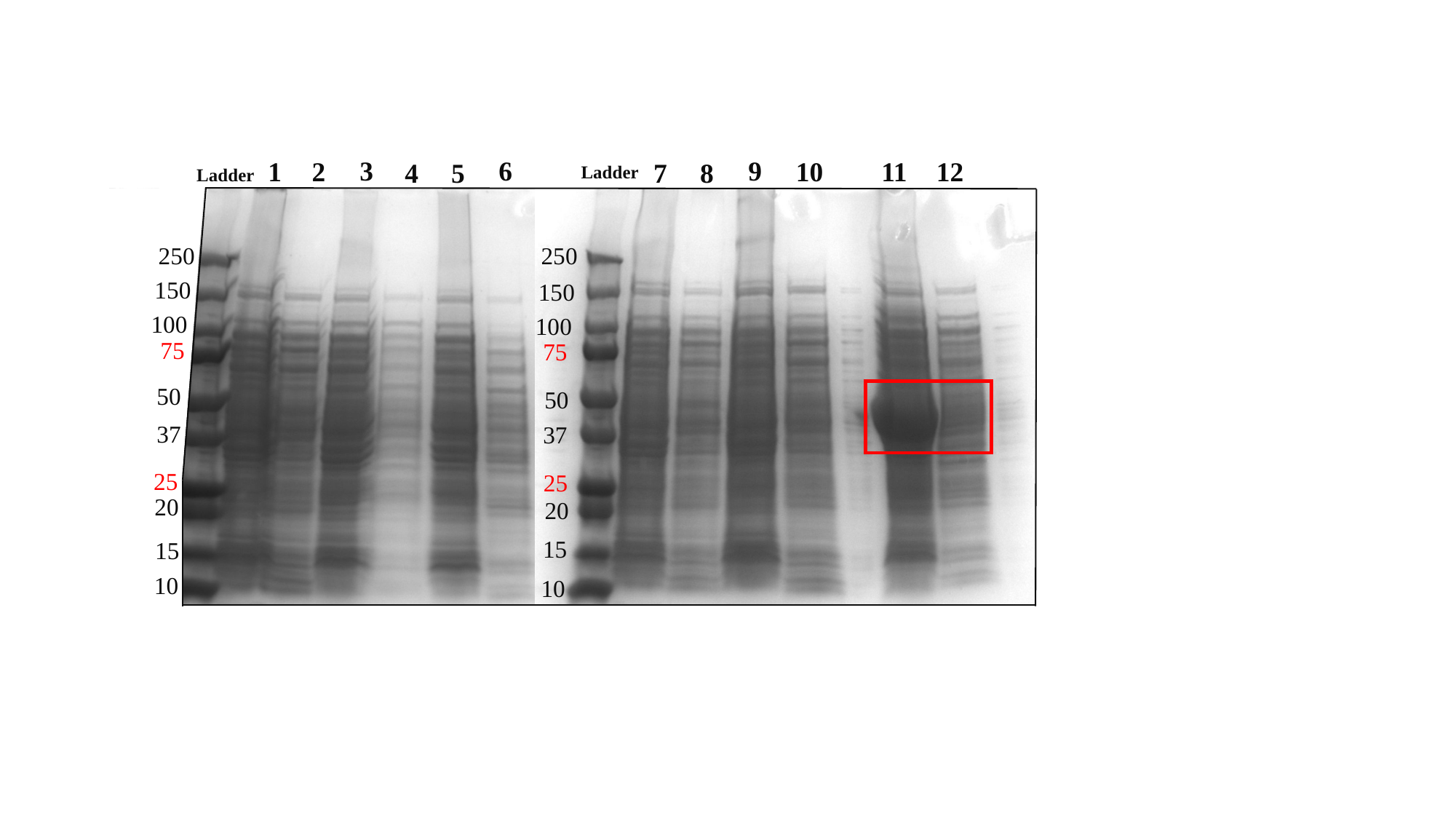

3
6
9
1
2
10
11
12
5
7
8
4
Ladder
Ladder
250
250
150
150
100
100
50
75
75
37
50
50
37
37
25
25
20
20
15
15
10
10

## Slide 3
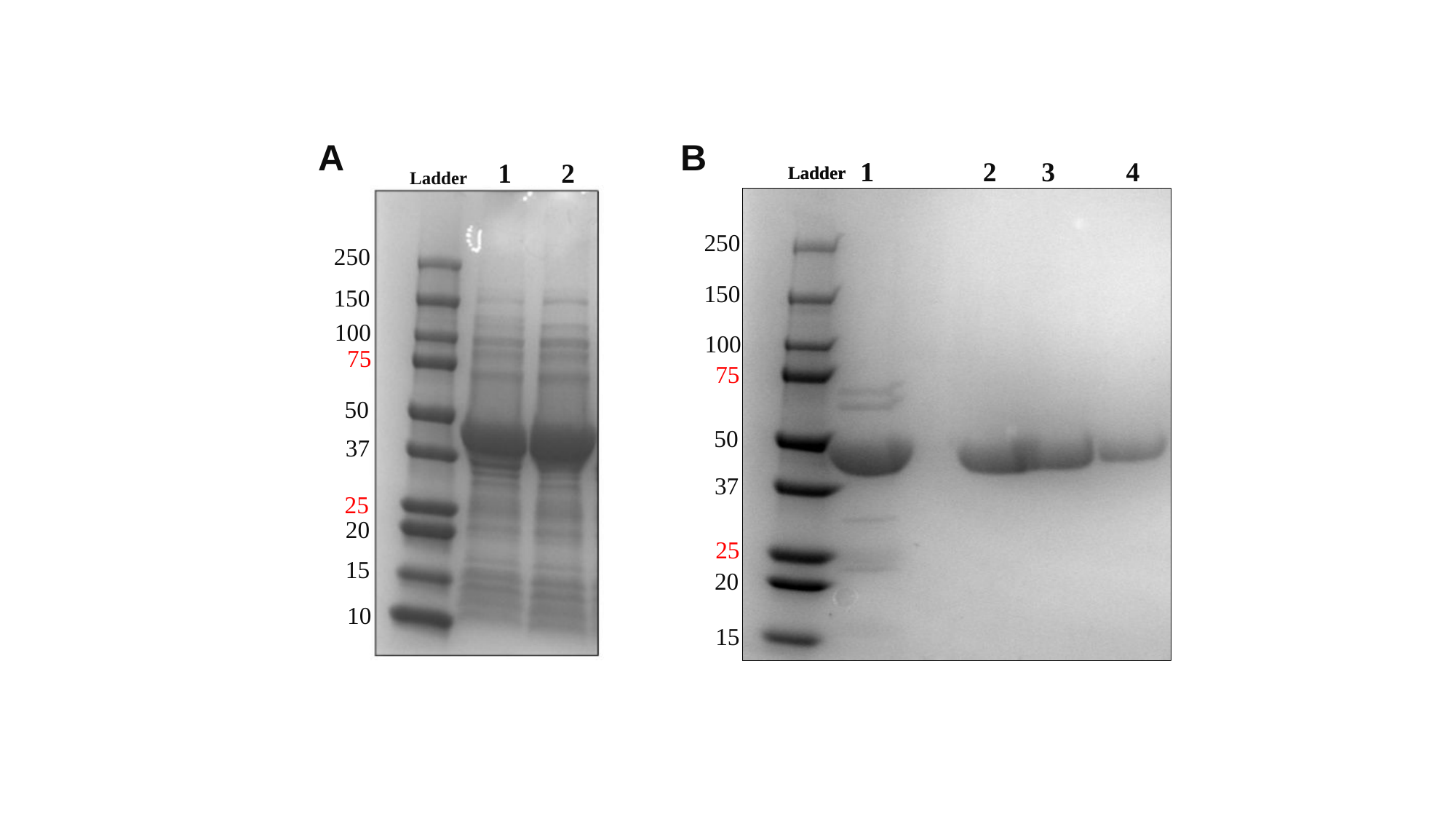

B
A
1
4
3
2
1
2
1
Ladder
Ladder
Ladder
250
250
150
150
100
100
75
75
50
50
37
37
25
20
25
15
20
10
15

## Slide 4
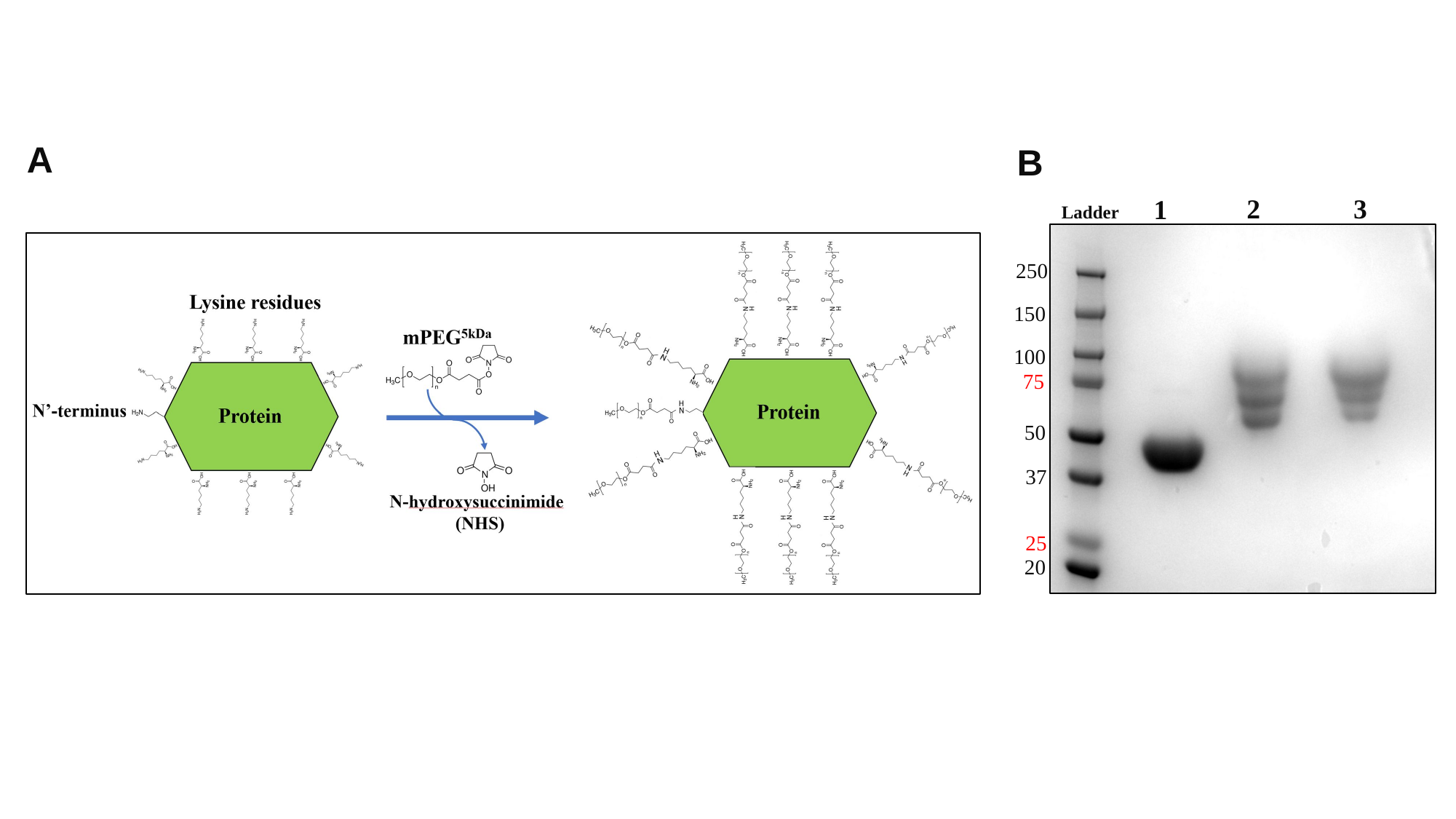

A
B
2
3
1
Ladder
250
150
100
75
50
37
25
20

## Slide 5
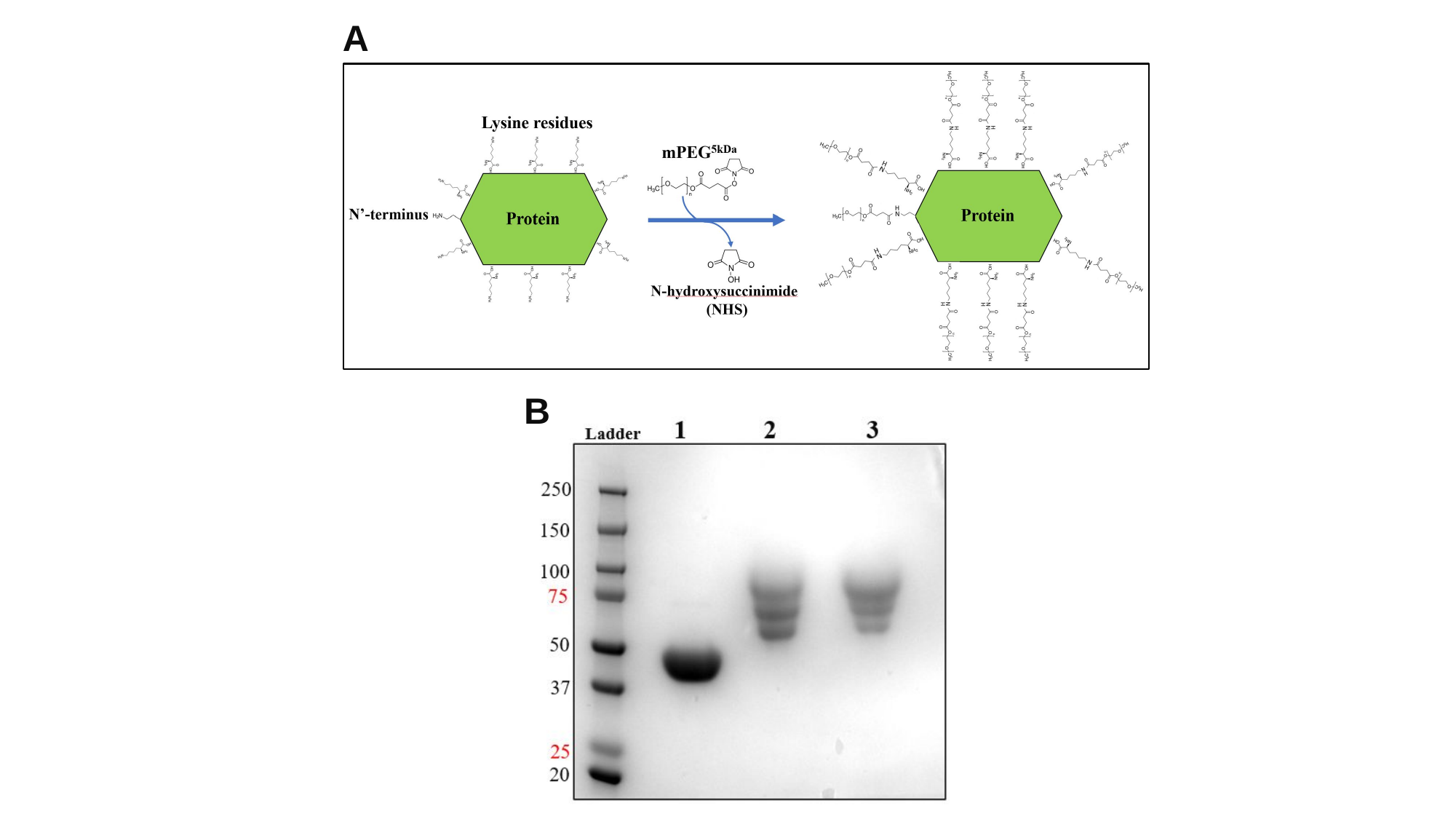

A
B

## Slide 6
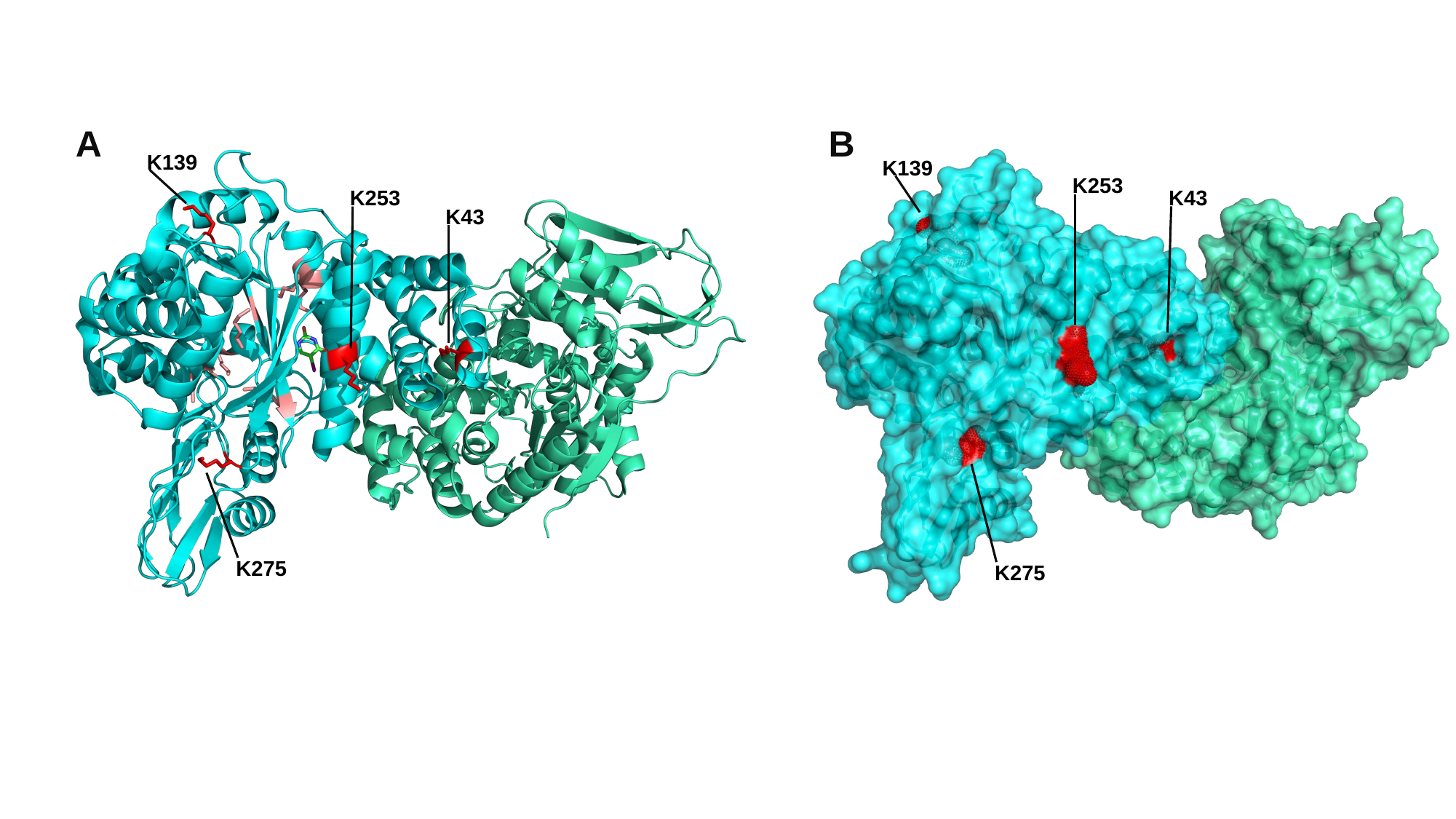

A
B
K139
K139
K253
K43
K253
K43
K275
K275

## Slide 7
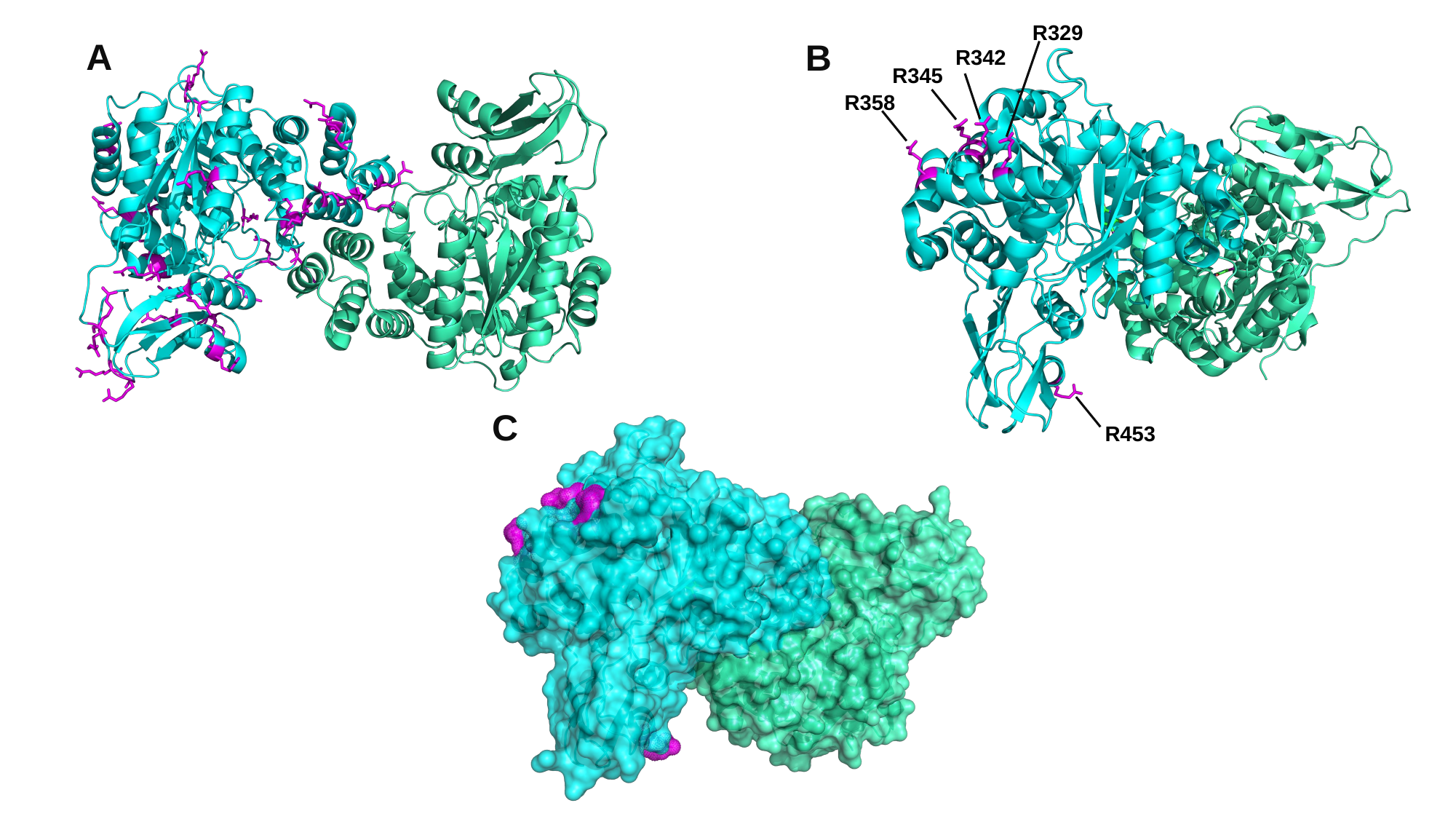

R329
A
B
R342
R345
R358
C
R453

## Slide 8
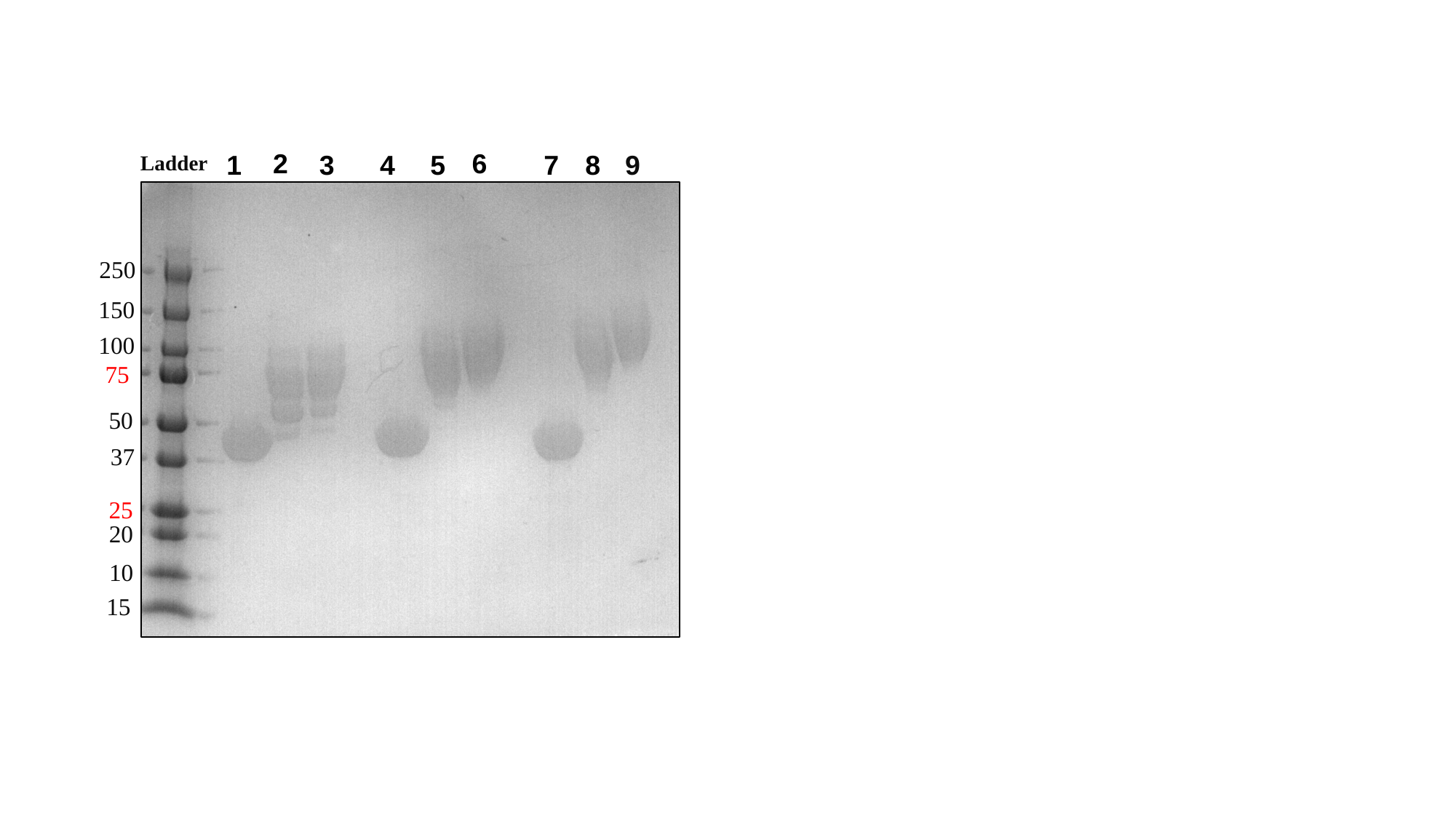

2
6
1
3
5
7
8
9
4
Ladder
250
150
100
75
50
37
25
20
10
15

## Slide 9
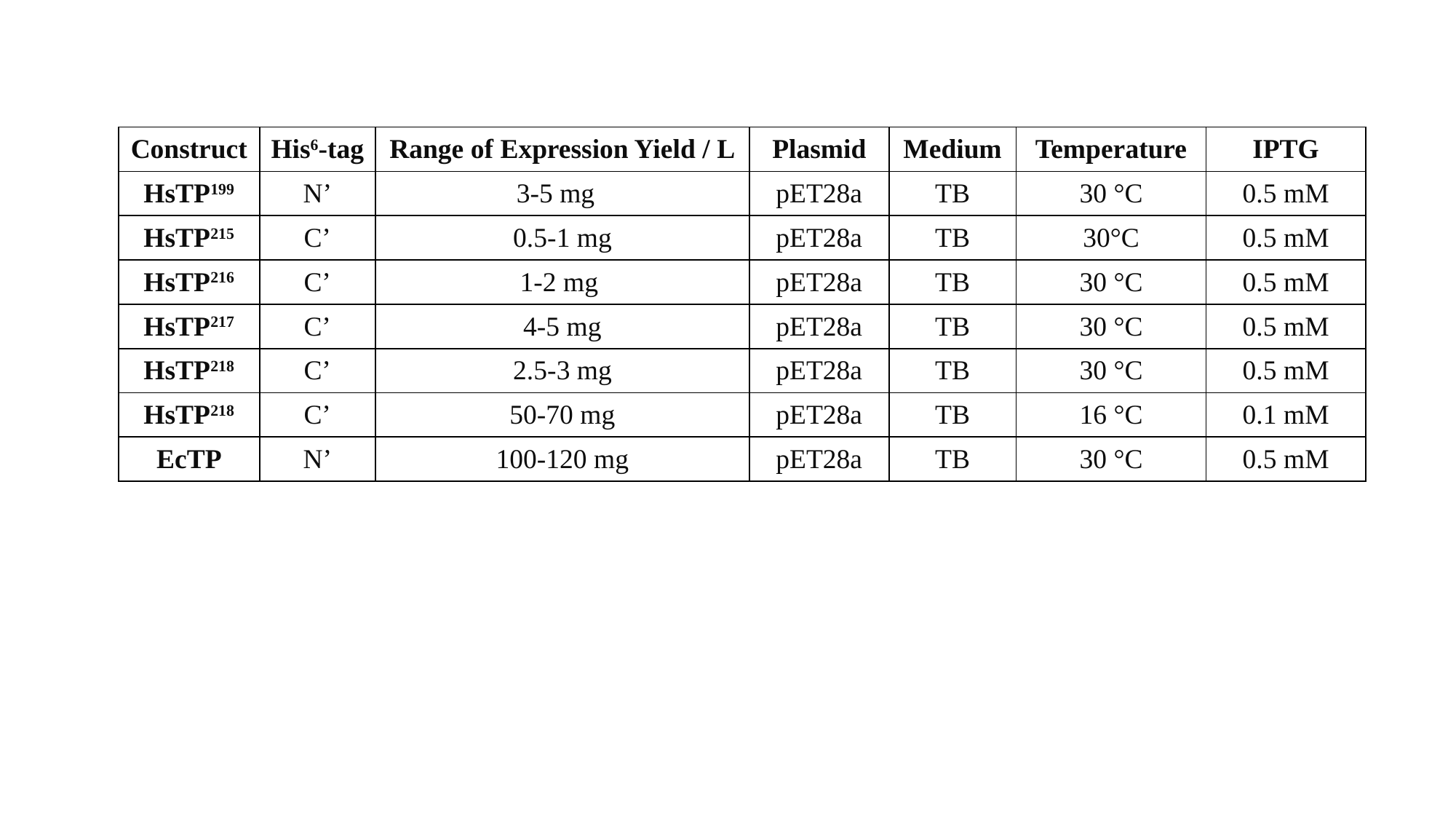

| Construct | His6-tag | Range of Expression Yield / L | Plasmid | Medium | Temperature | IPTG |
| --- | --- | --- | --- | --- | --- | --- |
| HsTP199 | N’ | 3-5 mg | pET28a | TB | 30 °C | 0.5 mM |
| HsTP215 | C’ | 0.5-1 mg | pET28a | TB | 30°C | 0.5 mM |
| HsTP216 | C’ | 1-2 mg | pET28a | TB | 30 °C | 0.5 mM |
| HsTP217 | C’ | 4-5 mg | pET28a | TB | 30 °C | 0.5 mM |
| HsTP218 | C’ | 2.5-3 mg | pET28a | TB | 30 °C | 0.5 mM |
| HsTP218 | C’ | 50-70 mg | pET28a | TB | 16 °C | 0.1 mM |
| EcTP | N’ | 100-120 mg | pET28a | TB | 30 °C | 0.5 mM |

## Slide 10
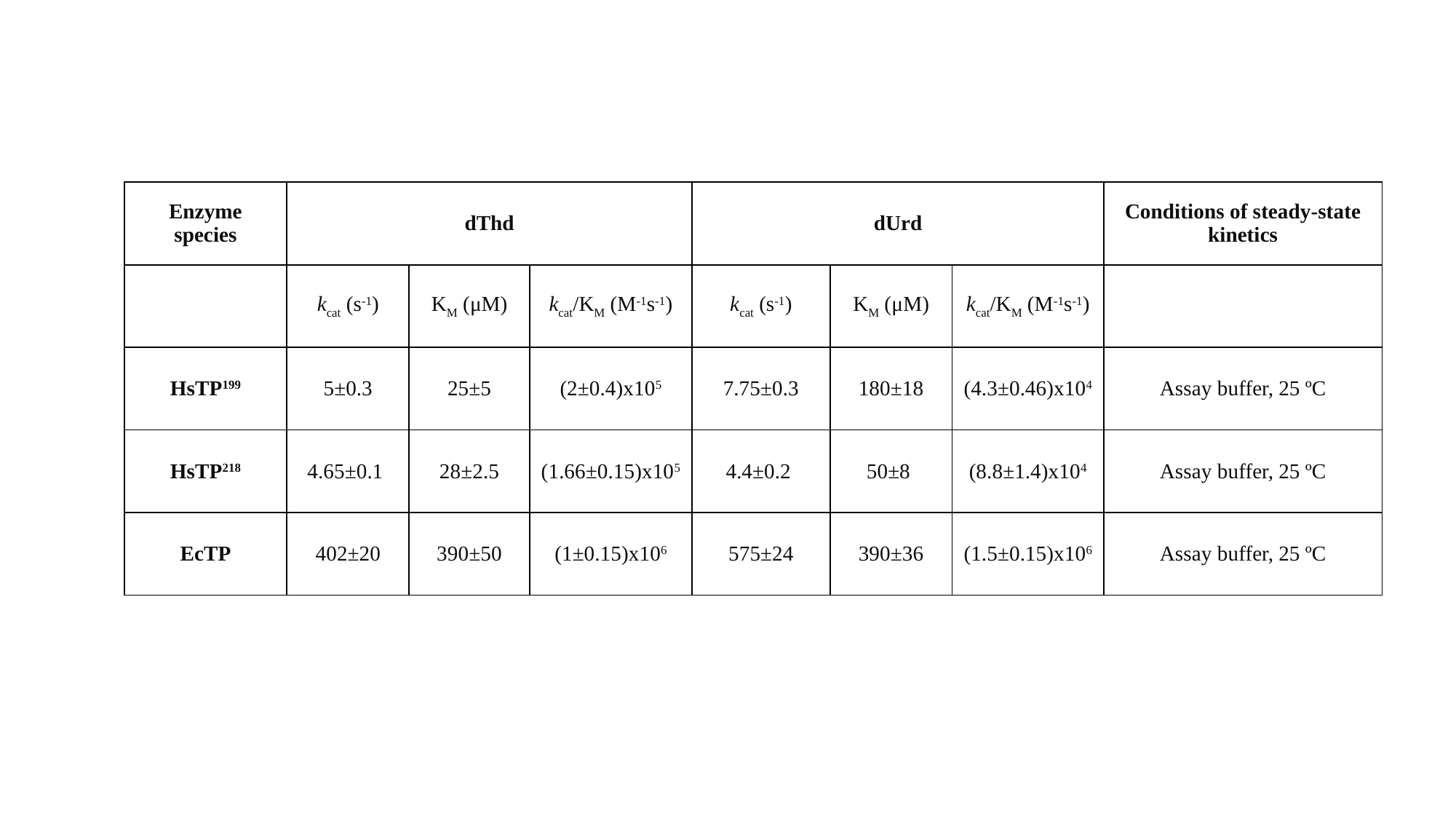

| Enzyme species | dThd | | | dUrd | | | Conditions of steady-state kinetics |
| --- | --- | --- | --- | --- | --- | --- | --- |
| | kcat (s-1) | KM (μM) | kcat/KM (M-1s-1) | kcat (s-1) | KM (μM) | kcat/KM (M-1s-1) | |
| HsTP199 | 5±0.3 | 25±5 | (2±0.4)x105 | 7.75±0.3 | 180±18 | (4.3±0.46)x104 | Assay buffer, 25 ºC |
| HsTP218 | 4.65±0.1 | 28±2.5 | (1.66±0.15)x105 | 4.4±0.2 | 50±8 | (8.8±1.4)x104 | Assay buffer, 25 ºC |
| EcTP | 402±20​ | 390±50​ | (1±0.15)x106​ | 575±24​ | 390±36​ | (1.5±0.15)x106​ | Assay buffer, 25 ºC |

## Slide 11
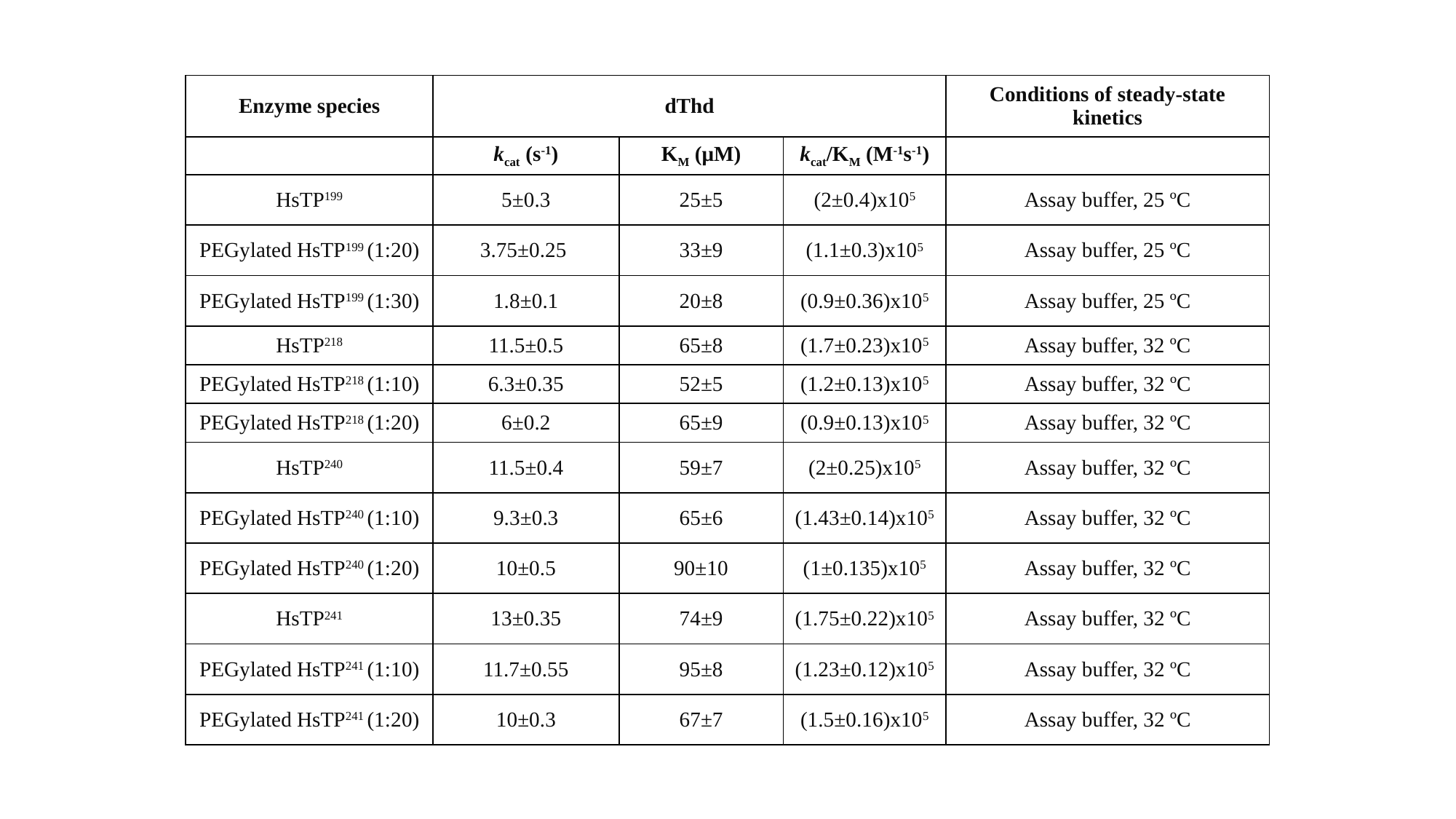

| Enzyme species | dThd | | | Conditions of steady-state kinetics |
| --- | --- | --- | --- | --- |
| | kcat (s-1) | KM (μM) | kcat/KM (M-1s-1) | |
| HsTP199 | 5±0.3 | 25±5 | (2±0.4)x105 | Assay buffer, 25 ºC |
| PEGylated HsTP199 (1:20) | 3.75±0.25 | 33±9 | (1.1±0.3)x105 | Assay buffer, 25 ºC |
| PEGylated HsTP199 (1:30) | 1.8±0.1​ | 20±8​ | (0.9±0.36)x105​ | Assay buffer, 25 ºC |
| HsTP218 | 11.5±0.5 | 65±8 | (1.7±0.23)x105 | Assay buffer, 32 ºC |
| PEGylated HsTP218 (1:10) | 6.3±0.35 | 52±5 | (1.2±0.13)x105 | Assay buffer, 32 ºC |
| PEGylated HsTP218 (1:20) | 6±0.2 | 65±9 | (0.9±0.13)x105 | Assay buffer, 32 ºC |
| HsTP240 | 11.5±0.4 | 59±7 | (2±0.25)x105 | Assay buffer, 32 ºC |
| PEGylated HsTP240 (1:10) | 9.3±0.3 | 65±6 | (1.43±0.14)x105 | Assay buffer, 32 ºC |
| PEGylated HsTP240 (1:20) | 10±0.5 | 90±10 | (1±0.135)x105 | Assay buffer, 32 ºC |
| HsTP241 | 13±0.35 | 74±9 | (1.75±0.22)x105 | Assay buffer, 32 ºC |
| PEGylated HsTP241 (1:10) | 11.7±0.55 | 95±8 | (1.23±0.12)x105 | Assay buffer, 32 ºC |
| PEGylated HsTP241 (1:20) | 10±0.3 | 67±7 | (1.5±0.16)x105 | Assay buffer, 32 ºC |

## Slide 12
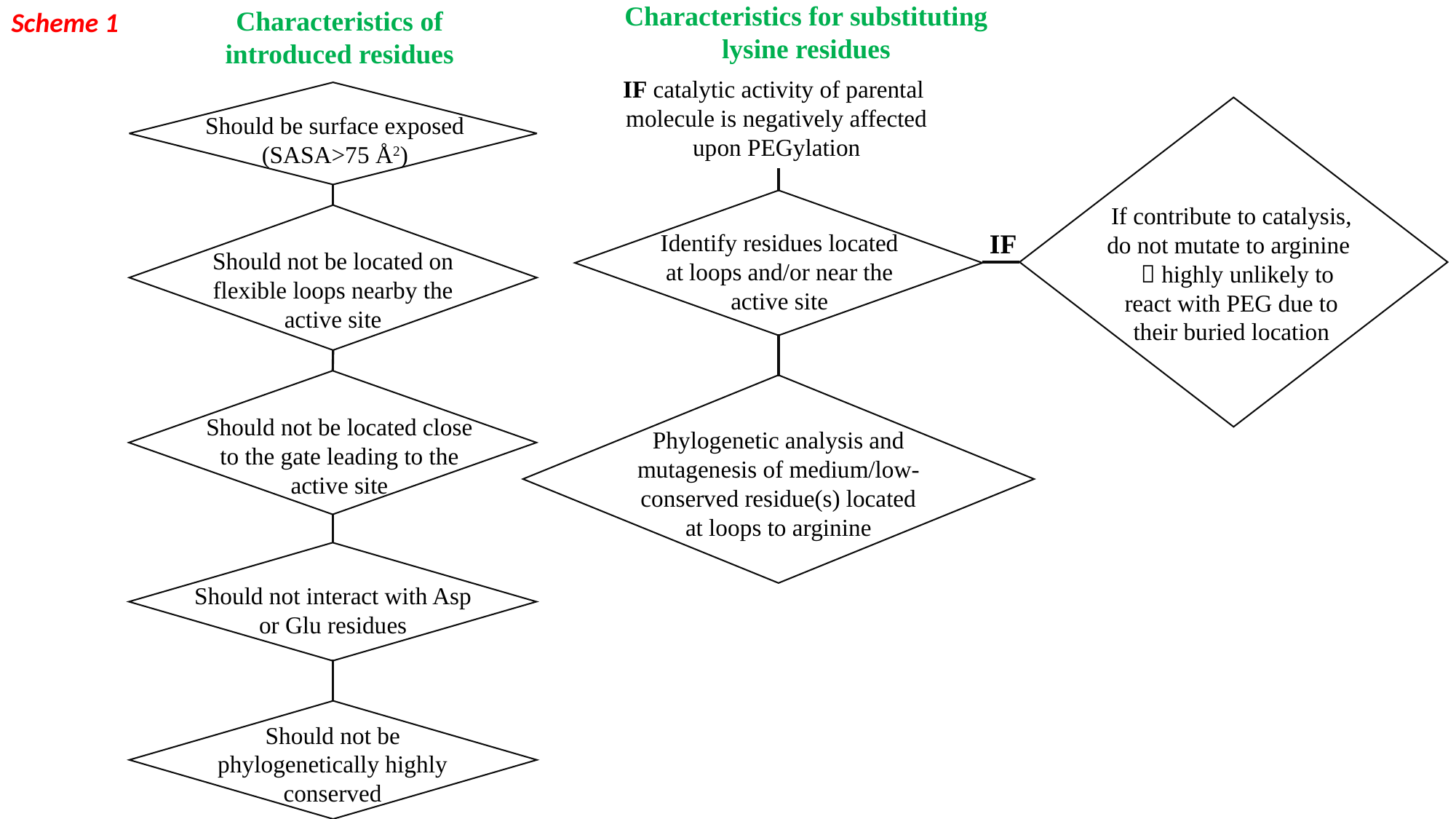

Scheme 1
Characteristics for substituting lysine residues
Characteristics of introduced residues
IF catalytic activity of parental molecule is negatively affected upon PEGylation
Should be surface exposed (SASA>75 Å2)
If contribute to catalysis, do not mutate to arginine
  highly unlikely to react with PEG due to their buried location
IF
Identify residues located at loops and/or near the active site
Should not be located on flexible loops nearby the active site
Should not be located close to the gate leading to the active site
Phylogenetic analysis and mutagenesis of medium/low-conserved residue(s) located at loops to arginine
Should not interact with Asp or Glu residues
Should not be phylogenetically highly conserved

## Slide 13
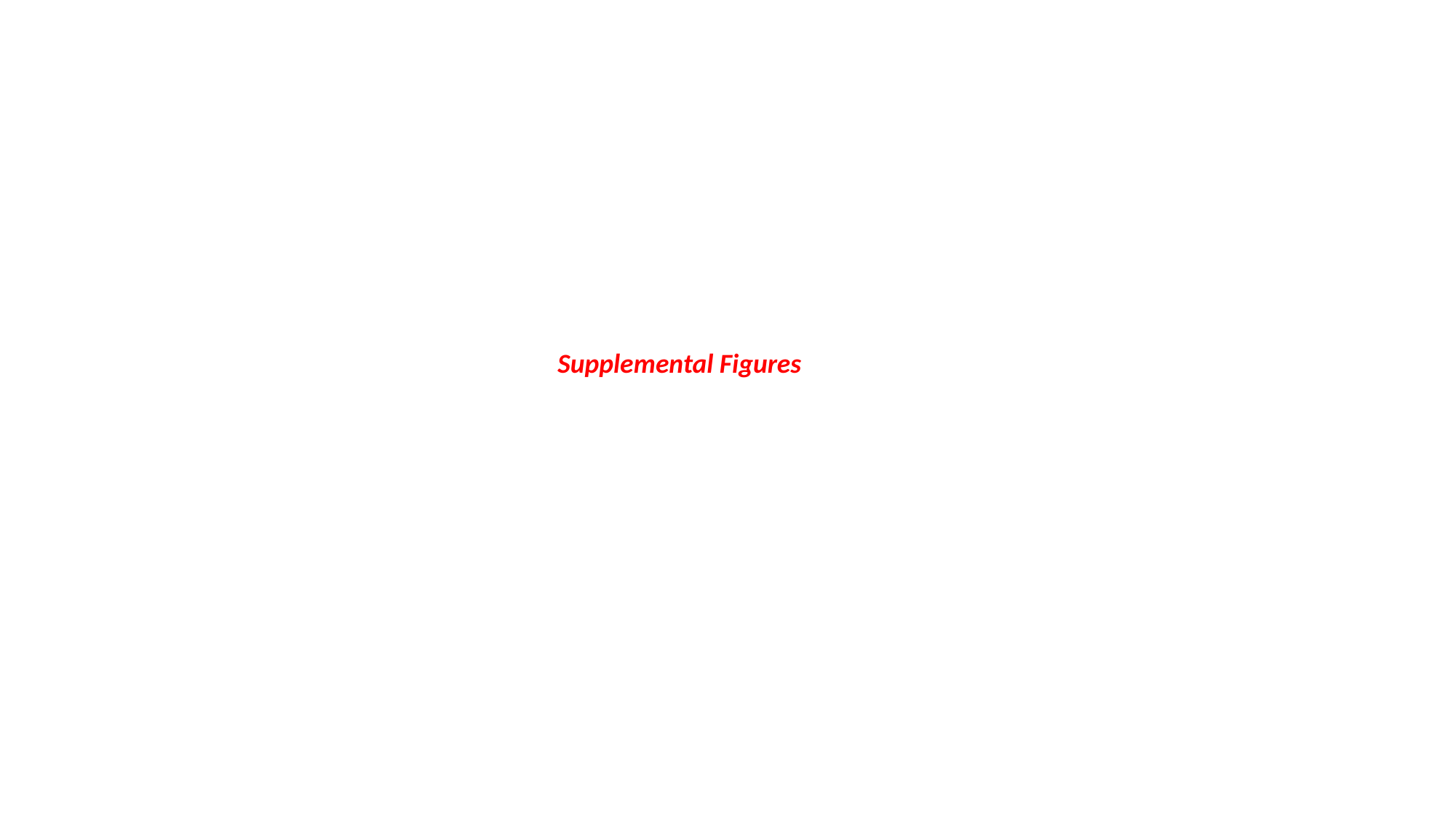

Supplemental Figures

## Slide 14
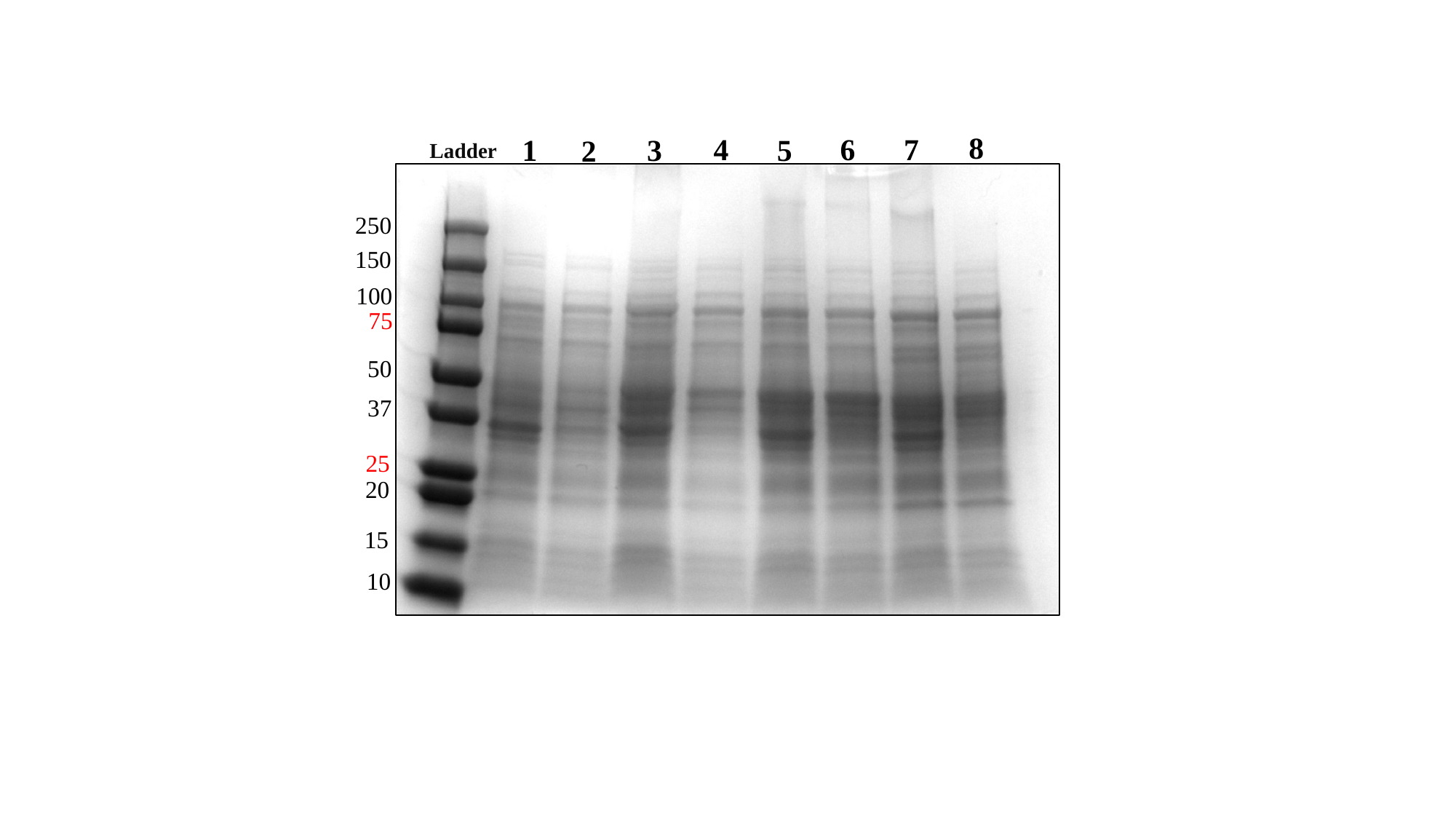

8
6
7
4
1
3
5
2
Ladder
250
150
100
75
50
37
25
20
15
10

## Slide 15
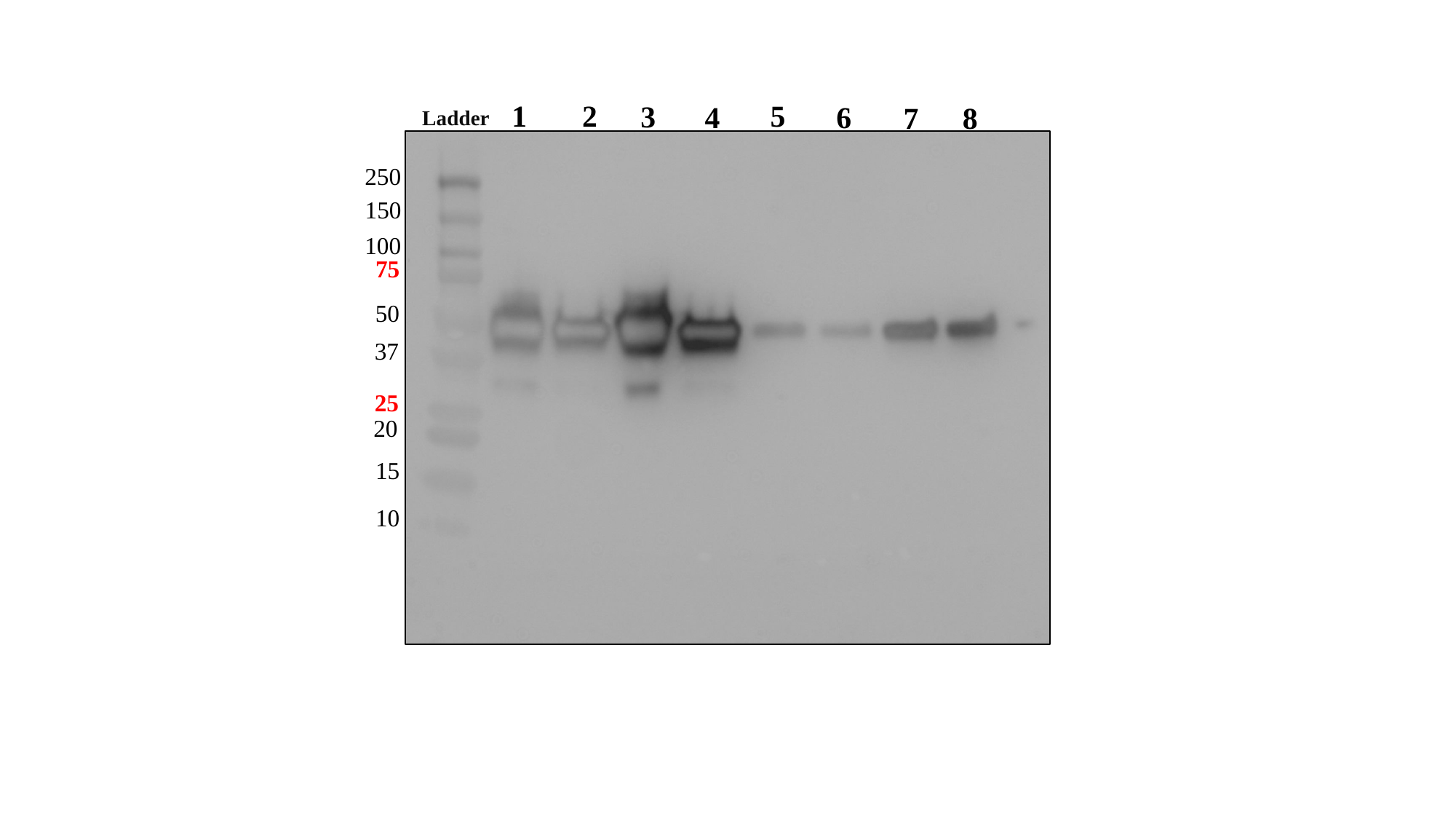

1
5
2
3
6
4
7
8
Ladder
250
150
100
75
50
37
25
20
15
10

## Slide 16
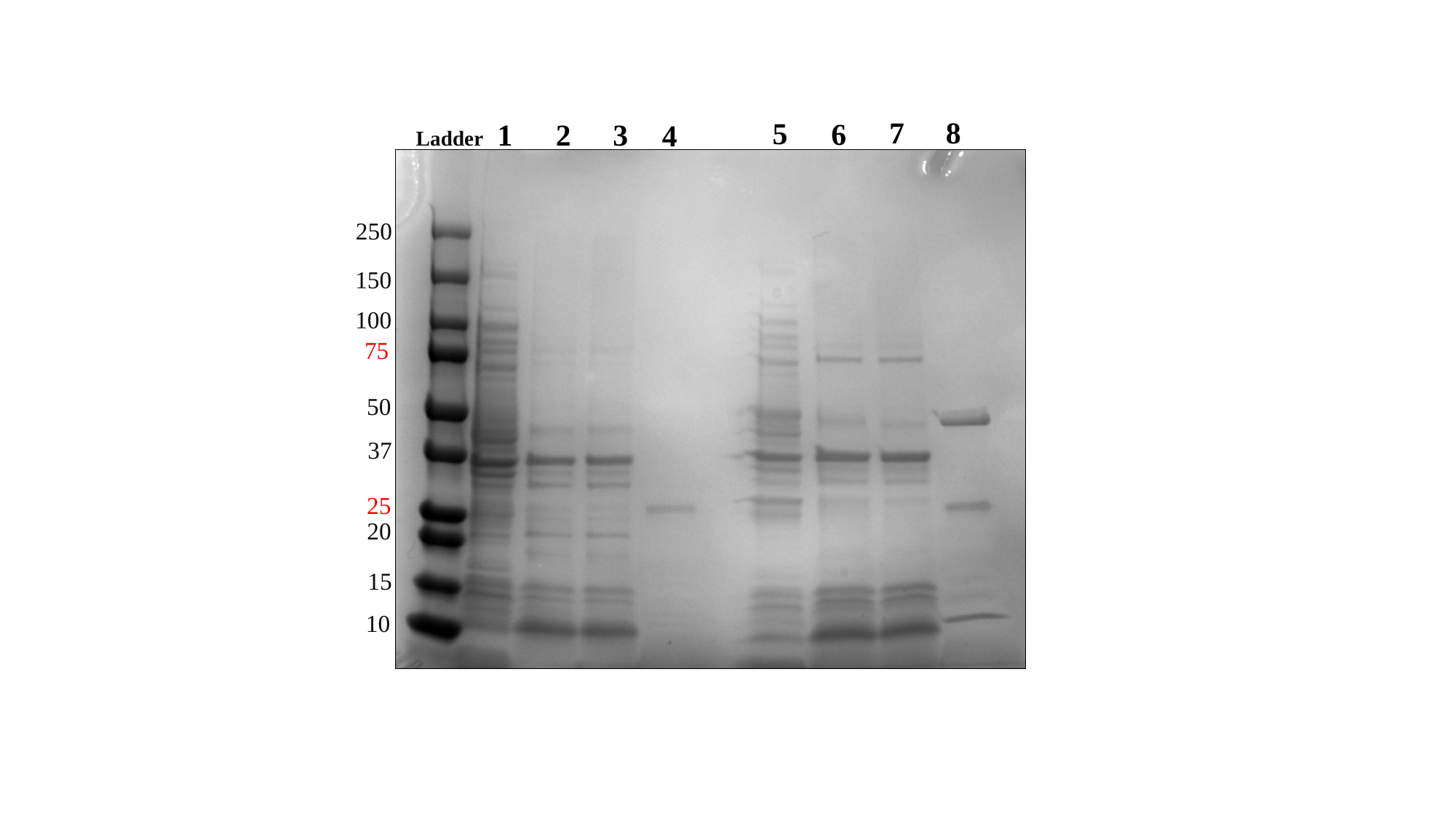

7
8
5
6
1
2
3
4
Ladder
250
150
100
75
50
37
25
20
15
10

## Slide 17
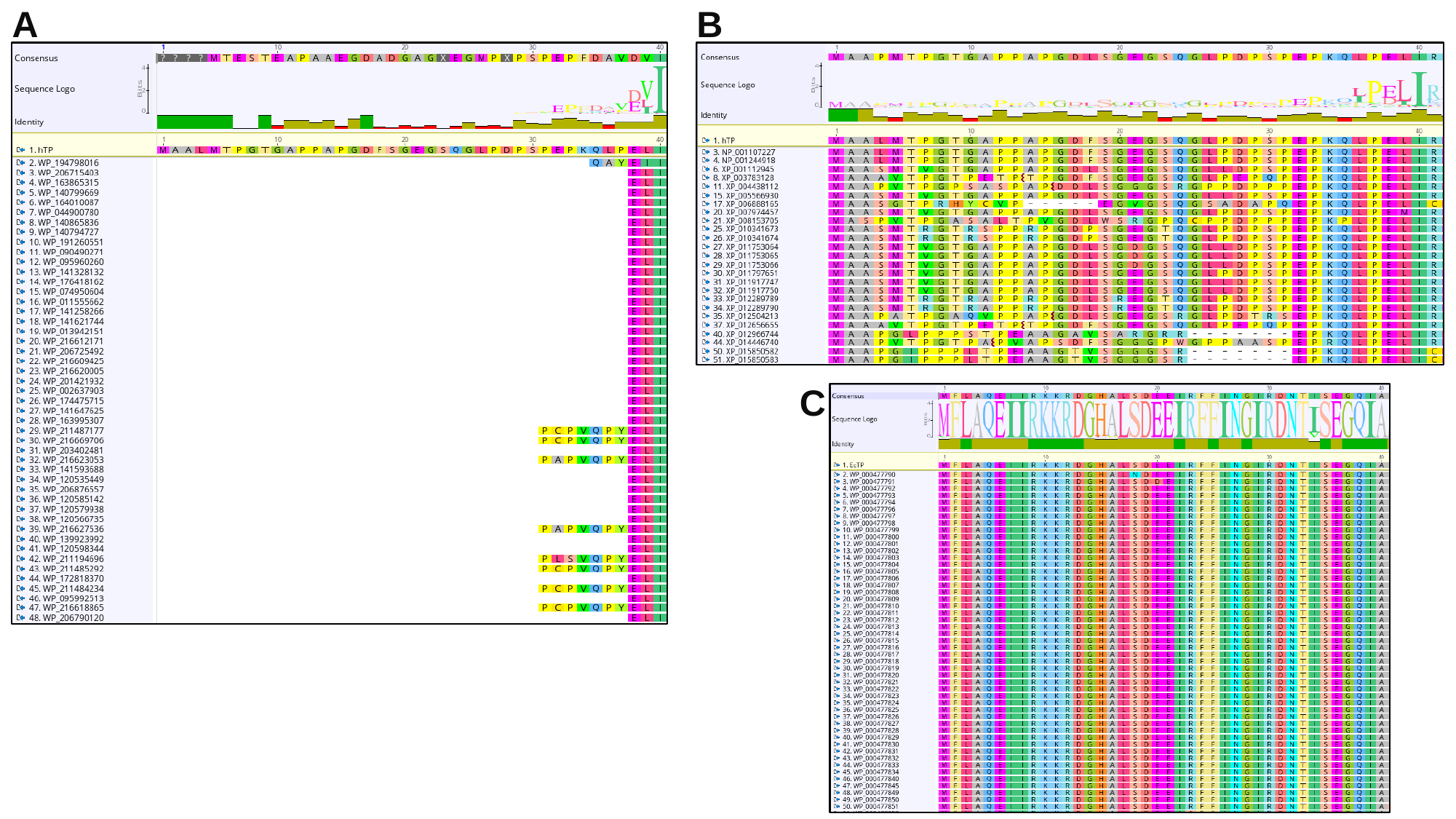

A
B
C

## Slide 18
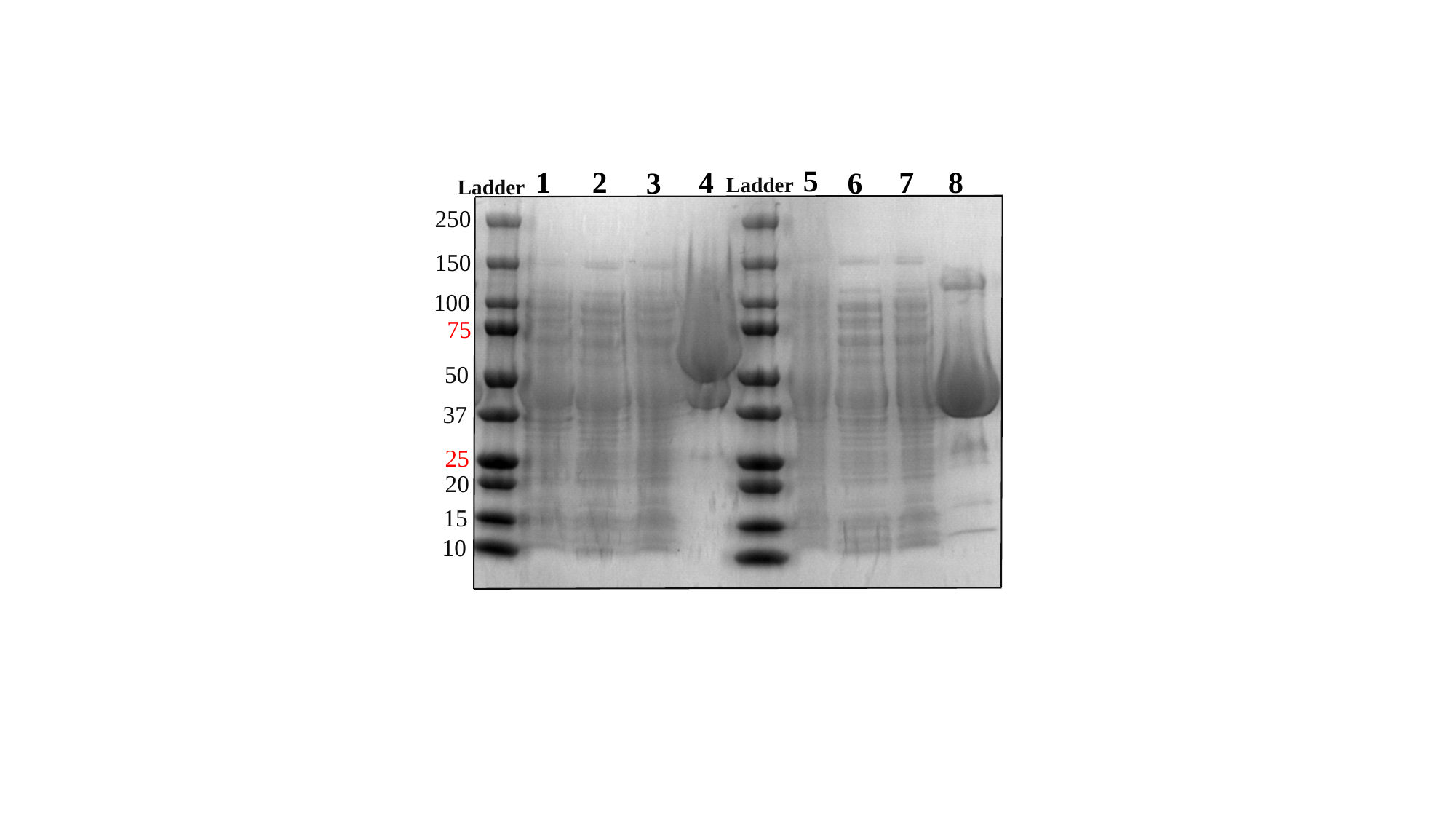

5
7
1
2
8
4
6
3
Ladder
Ladder
250
150
100
75
50
37
25
20
15
10

## Slide 19
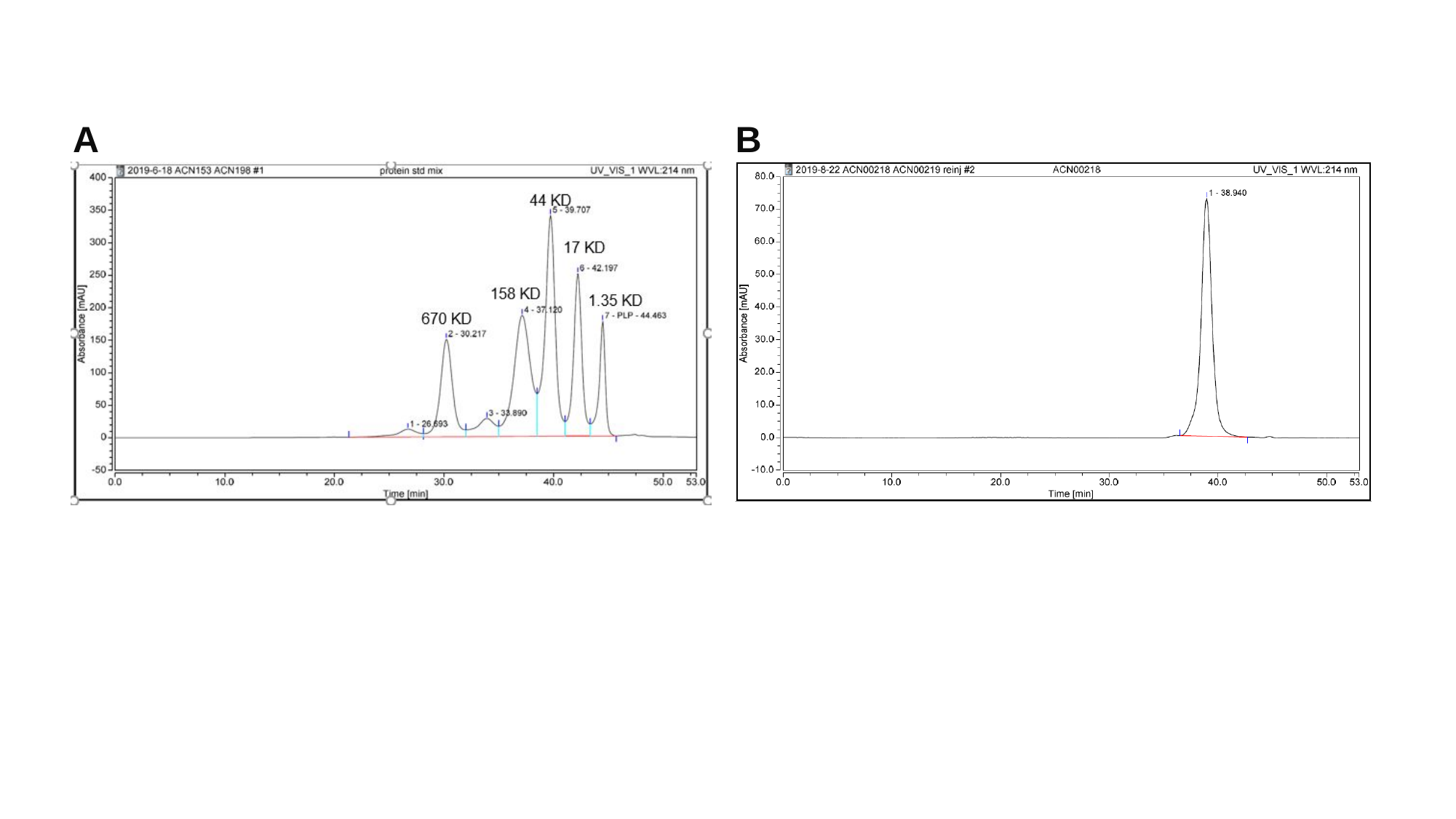

A
B

## Slide 20
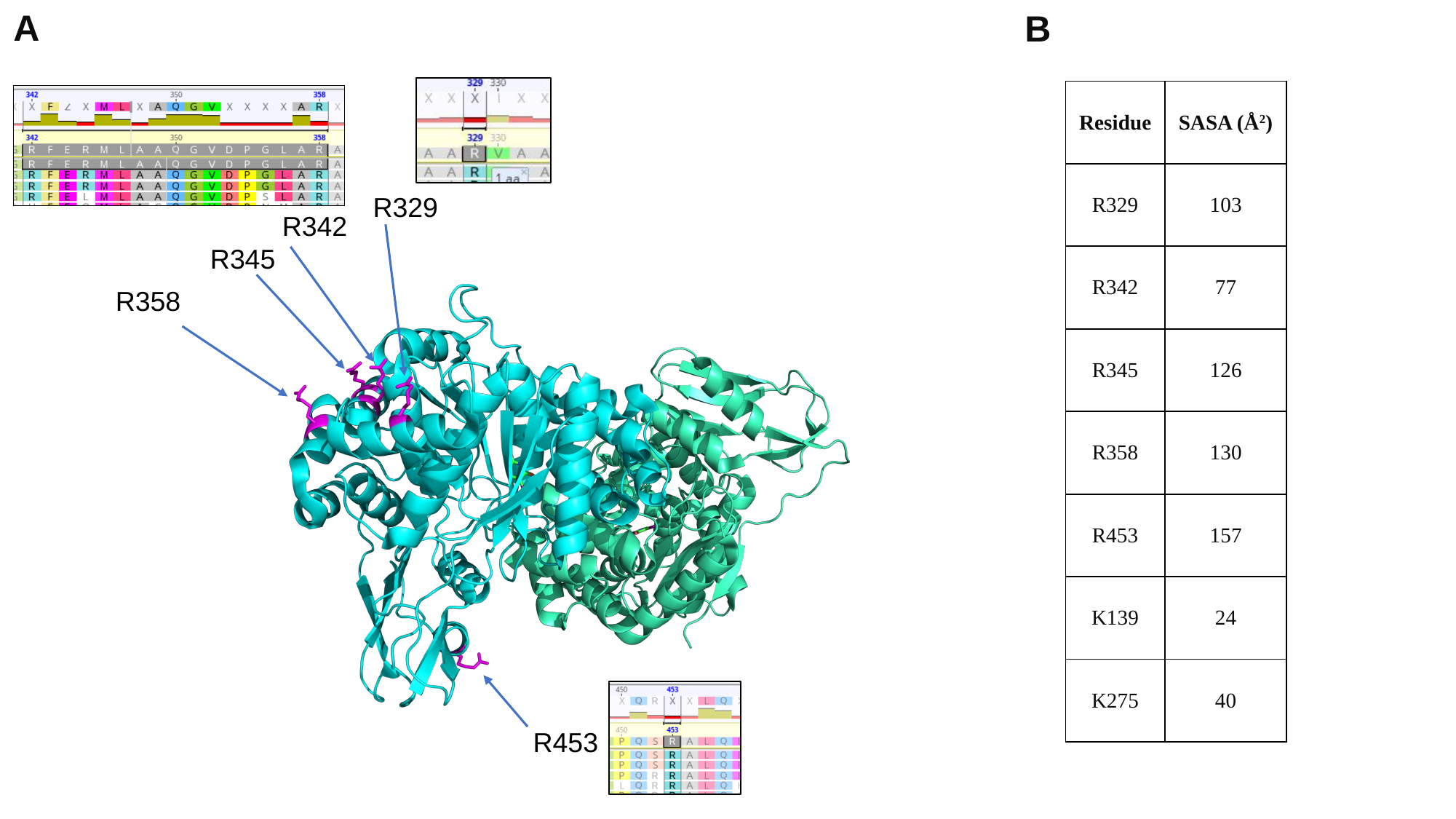

B
A
| Residue | SASA (Å2) |
| --- | --- |
| R329 | 103 |
| R342 | 77 |
| R345 | 126 |
| R358 | 130 |
| R453 | 157 |
| K139 | 24 |
| K275 | 40 |
R329
R342
R345
R358
R453

## Slide 21
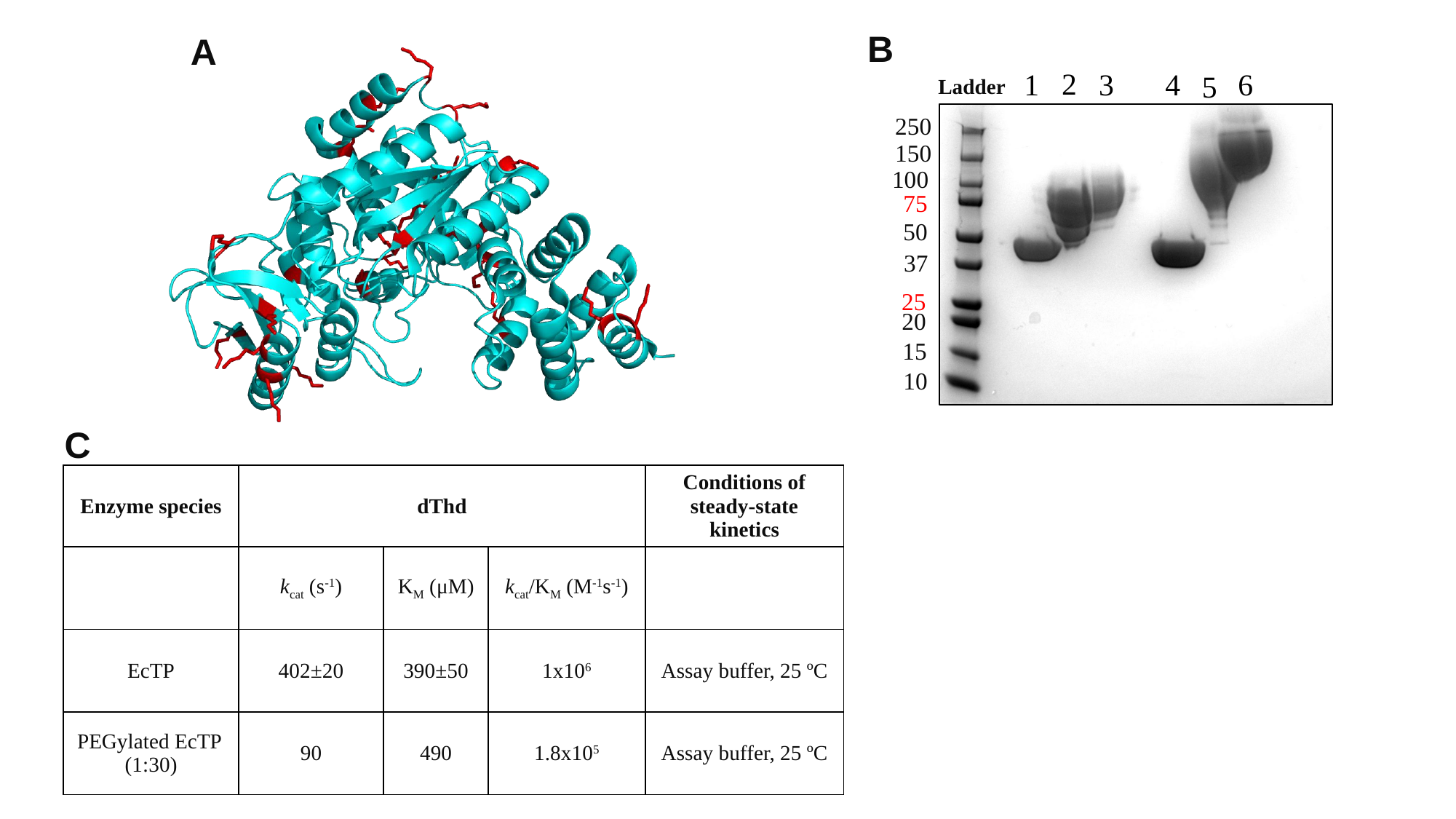

B
A
2
1
3
6
4
5
Ladder
250
150
100
75
50
37
25
20
15
10
C
| Enzyme species | dThd | | | Conditions of steady-state kinetics |
| --- | --- | --- | --- | --- |
| | kcat (s-1) | KM (μM) | kcat/KM (M-1s-1) | |
| EcTP | 402±20​ | 390±50​ | 1x10^6^​ | Assay buffer, 25 ºC |
| PEGylated EcTP (1:30) | 90 | 490 | 1.8x10^5^​ | Assay buffer, 25 ºC |

## Slide 22
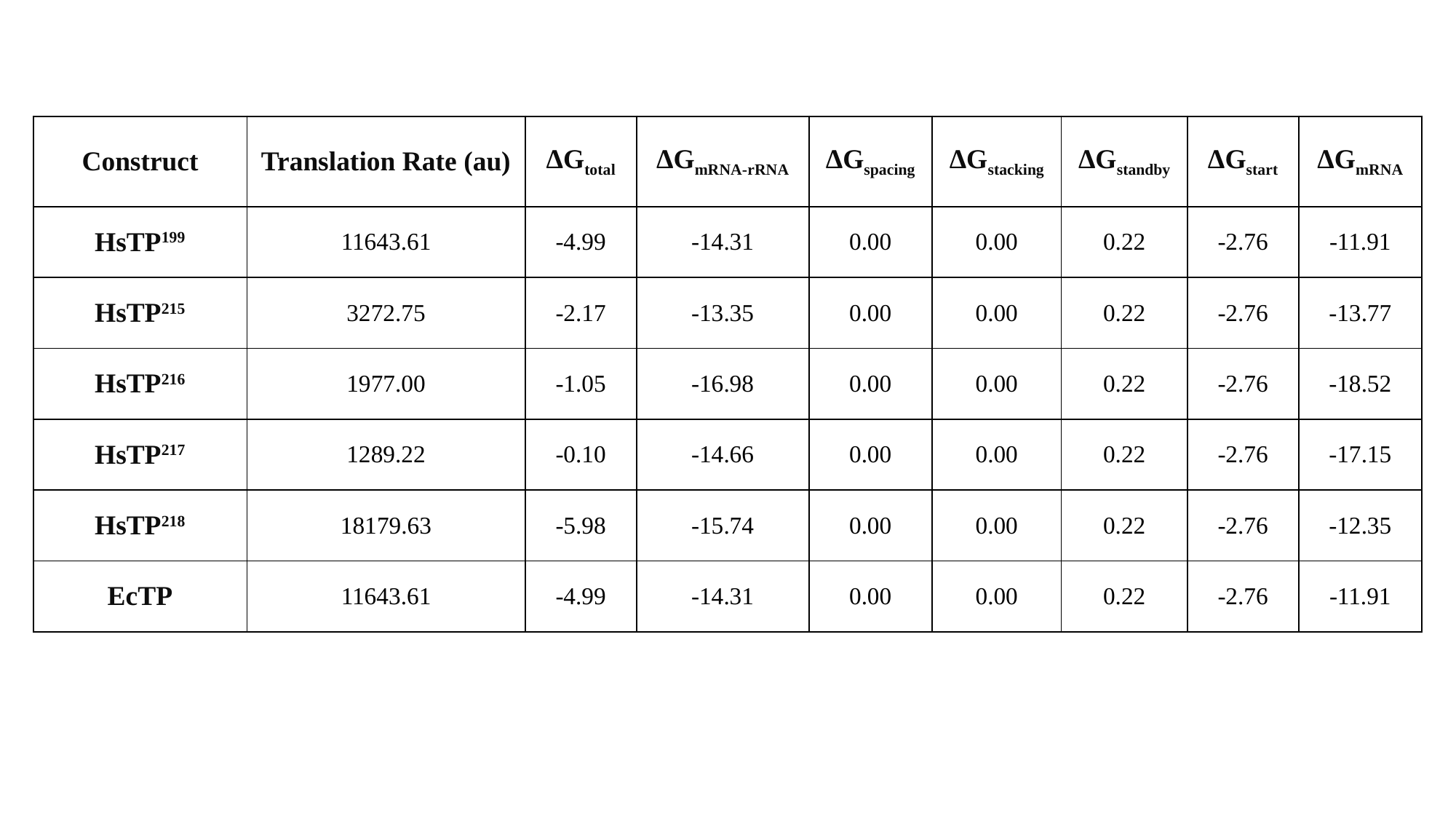

| Construct | Translation Rate (au) | ΔGtotal | ΔGmRNA-rRNA | ΔGspacing | ΔGstacking | ΔGstandby | ΔGstart | ΔGmRNA |
| --- | --- | --- | --- | --- | --- | --- | --- | --- |
| HsTP199 | 11643.61 | -4.99 | -14.31 | 0.00 | 0.00 | 0.22 | -2.76 | -11.91 |
| HsTP215 | 3272.75 | -2.17 | -13.35 | 0.00 | 0.00 | 0.22 | -2.76 | -13.77 |
| HsTP216 | 1977.00 | -1.05 | -16.98 | 0.00 | 0.00 | 0.22 | -2.76 | -18.52 |
| HsTP217 | 1289.22 | -0.10 | -14.66 | 0.00 | 0.00 | 0.22 | -2.76 | -17.15 |
| HsTP218 | 18179.63 | -5.98 | -15.74 | 0.00 | 0.00 | 0.22 | -2.76 | -12.35 |
| EcTP | 11643.61 | -4.99 | -14.31 | 0.00 | 0.00 | 0.22 | -2.76 | -11.91 |
