# Supplementary material for: Engineering of the Recombinant Expression and PEGylation Efficiency of the Therapeutic Enzyme Human Thymidine Phosphorylase
Source: Front Bioeng Biotechnol. 2021 Dec 17;9:793985. doi: 10.3389/fbioe.2021.793985 (PMC8718881; doi:10.3389/fbioe.2021.793985)
Supplement: Supplementary file 10 [file Table2.docx]

| **Construct** | **5’ Primer** | **3’ Primer** |
| --- | --- | --- |
| pET28a-HsTP^199^ | AGATATA***CCATGG***GCGGTCATCACCACCATCATCACGGTAGCGGTGCC | CCGCCG***GAATTC***TCATTGTTGCGGAGGGAGGACGAGTTCCGC |
| pJC20- HsTP^199^ | *GGGAATTC****CATATG***GGCGGTCATCACCACCATCATCACGGTAGCGGTGCC | CGC***GGATCC***TCATTGTTGCGGAGGGAGGACGAGTTCCGC |
| pET28a-EcTP | AGATATA***CCATGG***GCGGTCATCACCACCATCATCACGGTAGCGGTTT TCTCGCACAAGAAATTATTCG | CCGCCG***GAATTC***TTATTCGCTGATACGGCGATAGACAGTTGG |
| pET28a-HsTP^215^ | AGATATA***CCATGG***GCGCGGCGCTTATGACCCCTGGAACTGGTGCCCCGCCTGCGCCAGGTGATTTTTCTGG | CCGCCG***GAATTC***TCAGCCGTGATGATGGTGGTGATGACCTTGTTGCGGAGGGAGGACGAGTTCCGC |
| pET28a-HsTP^216^ | AGATATA***CCATGG***GCGCCCCGCCTGCGCCAGGTGATTTTTCTGGTGAGGG | CCGCCG***GAATTC***TCAGCCGTGATGATGGTGGTGATGACCTTGTTGCGGAGGGAGGACGAGTTCCGC |
| pET28a-HsTP^217^ | AGATATA***CCATGG***GCTCTGGTGAGGGTTCCCAGGGTCTGCCAGACCC | CCGCCG***GAATTC***TCAGCCGTGATGATGGTGGTGATGACCTTGTTGCGGAGGGAGGACGAGTTCCGC |
| pET28a-HsTP^218^ | AGATATA***CCATGG***GCAAACAGCTCCCGGAACTGATCCGTATGAAACGCGACGG | CCGCCG***GAATTC***TCAGCCGTGATGATGGTGGTGATGACCTTGTTGCGGAGGGAGGACGAGTTCCGC |
| pET28a-HsTP^240^  pET28a-HsTP^241^ | - **K139R**: CTCCTGCACTCGCTGCATGTGGTTGCCGTGTTCCGATGATTAGCGGTCG - **K275R**: GCAGCAGCACTCACCGCGATGGACCGCCCTCTGGGCCGCTGTGTTGG - **R329K**: CGCAAGCGCAAGGTGCAGCAAAAGTAGCTGCCGCGCTGGATG - **R342K-R345K-R358K**: GGTTCTGCACTGGGTAAATTCGAAAAGATGCTCGCAGCACAGGGCGTTGATCCGGGCCTGGCTAAAGCCCTCTGCTCTGGCTCTCCGGC - **R453K**: GCACTGAGCGGTCCGCAATCTAAAGCGCTGCAAGAAGCCCTGG | - **K139R**: CGACCGCTAATCATCGGAACACGGCAACCACATGCAGCGAGTGCAGGAG - **K275R**: CCAACACAGCGGCCCAGAGGGCGGTCCATCGCGGTGAGTGCTGCTGC - **R329K**: CATCCAGCGCGGCAGCTACTTTTGCTGCACCTTGCGCTTGCG - **R342K-R345K-R358K**: GCCGGAGAGCCAGAGCAGAGGGCTTTAGCCAGGCCCGGATCAACGCCCTGTGCTGCGAGCATCTTTTCGAATTTACCCAGTGCAGAACC - **R453K**: CCAGGGCTTCTTGCAGCGCTTTAGATTGCGGACCGCTCAGTGC |
